# Nutritional Prevention of Oxidative Stress-Induced Cardiotoxicity in Pediatric Cardio-Oncology: Molecular Mechanisms and Translational Perspectives—A Narrative Review

**DOI:** 10.3390/ijms27177863

**Published:** 2026-09-02

**Authors:** Karmen Stankov, Bojan Stanimirov, Aleksandar Ninković, Maja Đanić, Slavica Lazarević, Dragana Zaklan, Nebojša Pavlović

**Affiliations:** 1 Department of Biochemistry, Faculty of Medicine, University of Novi Sad, 21000 Novi Sad, Serbia; karmen.stankov@mf.uns.ac.rs; 2 Faculty of Medicine, University of Novi Sad, 21000 Novi Sad, Serbia; 902030d25@mf.uns.ac.rs; 3 Department of Pharmacology, Toxicology and Clinical Pharmacology, Faculty of Medicine, University of Novi Sad, 21000 Novi Sad, Serbia; maja.djanic@mf.uns.ac.rs (M.Đ.); slavica.lazarevic@mf.uns.ac.rs (S.L.); 4 Department of Pharmacy, Faculty of Medicine, University of Novi Sad, 21000 Novi Sad, Serbia; dragana.zaklan@mf.uns.ac.rs (D.Z.); nebojsa.pavlovic@mf.uns.ac.rs (N.P.)

**Keywords:** anthracyclines, doxorubicin, radiotherapy, cardio-oncology, mitochondrial dysfunction, polyphenols, nutraceuticals, childhood cancer survivors, epigenetics, gut microbiota

## Abstract

Cancer therapy-related cardiotoxicity has emerged as a major challenge in contemporary oncology, particularly in long-term survivors of childhood malignancies. Oxidative stress (OS)-induced cardiotoxicity represents a key mechanistic pathway underlying myocardial injury caused by numerous anti-neoplastic drugs, most notably anthracycline-based chemotherapy, while OS also critically contributes to radiotherapy-induced cardiotoxicity. Excessive reactive oxygen and nitrogen species generation promotes mitochondrial dysfunction, impaired calcium homeostasis, lipid peroxidation, endothelial injury, inflammatory activation, and cardiomyocyte apoptosis, ultimately leading to progressive cardiac remodeling and ventricular dysfunction. These processes are of particular concern in pediatric cancer survivors, especially children treated for leukemia, who face a substantially elevated lifetime risk of cardiovascular disease following treatment exposure. Increasing attention has, therefore, been directed toward nutritional strategies capable of modulating redox homeostasis and attenuating treatment-associated myocardial injury. Experimental and translational evidence suggests that selected dietary compounds and nutraceuticals, including polyphenols, omega-3 fatty acids, coenzyme Q10, selenium, antioxidant vitamins, nutrition-based epigenetic interventions and intestinal microbiota composition modulations may exert cardioprotective effects through preservation of mitochondrial integrity, enhancement of endogenous antioxidant defenses, and suppression of oxidative and inflammatory signaling pathways. Nevertheless, clinical implementation remains limited by insufficient standardization, heterogeneous study designs, and incomplete understanding of long-term efficacy and safety. This narrative review critically examines the molecular basis of the OS-induced cardiotoxicity and radiotherapy-induced cardiotoxicity and evaluates current evidence supporting nutrition-based cardioprotective interventions, with particular emphasis on pediatric leukemia survivors and the prevention of long-term cardiovascular complications following anticancer therapy.

## 1. Introduction

### 1.1. Burden of Cancer Therapy-Related Cardiotoxicity

Remarkable advances in cancer diagnosis and treatment have substantially improved survival rates across a broad spectrum of malignancies. Cancer survivorship has improved dramatically over the past decades, with 5-year survival rates exceeding 80% for many malignancies, including childhood acute lymphoblastic leukemia (ALL) [1]. However, this success has been accompanied by growing recognition of long-term treatment-related morbidity, particularly cardiovascular complications. Cancer therapy-related cardiotoxicity represents one of the most significant late effects, contributing substantially to morbidity and mortality in cancer survivors [2,3].

Anthracycline chemotherapeutic agents, including doxorubicin (DOX), daunorubicin, and epirubicin, remain cornerstone treatments for hematologic malignancies and solid tumors. Nevertheless, their clinical utility is limited by dose-dependent cardiotoxicity that may develop during treatment or manifest years to decades after exposure [4]. The incidence of anthracycline-induced cardiotoxicity (AIC) ranges from 5% to 48%, depending on cumulative dose, patient age, concurrent therapies, and cardiovascular risk factors [5]. Recent evidence indicates that nearly all major classes of anticancer therapies, including human epidermal growth factor receptor 2 (HER2)-targeted therapies, tyrosine kinase inhibitors, immune checkpoint inhibitors, possess varying degrees of cardiovascular toxicity potential [6]. Similarly, thoracic radiotherapy, particularly for breast cancer, lymphoma, and lung cancer, induces radiation-induced heart disease through endothelial injury, microvascular damage, and accelerated atherosclerosis [6,7].

### 1.2. Growing Population of Childhood Cancer Survivors

Over the past several decades, survival rates for childhood cancers have increased significantly owing to advances in multimodal treatment approaches, supportive care, and risk-adapted therapies. In many developed countries, more than eighty percent of children diagnosed with cancer now survive at least five years after diagnosis, resulting in a rapidly expanding population of childhood cancer survivors [8,9]. Cardiovascular disease has emerged as the leading cause of non-cancer morbidity and mortality in this population. Survivors exposed to anthracyclines and chest radiotherapy exhibit significantly increased risks of cardiomyopathy, heart failure, coronary artery disease, stroke, valvular disease, and cardiac mortality compared with age-matched controls [8,10]. Recent studies indicate that childhood cancer survivors experience an eight- to ten-fold higher rate of cardiac mortality than the general population and frequently develop cardiovascular complications decades earlier than expected [10,11]. Moreover, the cumulative burden of cardiovascular disease continues to increase throughout adulthood, reflecting the persistent and progressive nature of treatment-induced cardiac injury [9,12]. Childhood leukemia survivors represent a particularly important subgroup because anthracycline-based chemotherapy remains a cornerstone of treatment for ALL and other pediatric hematological malignancies [13]. Therefore, pediatric cancer survivors face particularly elevated lifetime cardiovascular risk, with cumulative anthracycline exposure during critical developmental periods predisposing to premature heart failure, arrhythmias, and coronary artery disease [1,10].

The growing population of cancer survivors has consequently transformed cardiotoxicity from a relatively uncommon adverse effect into a significant public health challenge. Cardiovascular disease is now recognized as one of the leading non-cancer causes of death among cancer survivors and frequently limits long-term quality of life and overall survival [3]. Accordingly, prevention of treatment-related cardiovascular injury has become a central objective of contemporary cardio-oncology [14]. The molecular pathogenesis of cancer therapy-related cardiotoxicity converges on oxidative stress (OS) as a central mechanism. Excessive generation of reactive oxygen species (ROS) and reactive nitrogen species (RNS) overwhelms endogenous antioxidant defenses, triggering mitochondrial dysfunction, lipid peroxidation, DNA damage, inflammatory activation, and ultimately cardiomyocyte apoptosis and necrosis [4,15].

Current strategies for preventing cancer therapy-related cardiotoxicity primarily focus on dose optimization, cardiac surveillance, and pharmacological interventions such as dexrazoxane (an iron-chelating agent and topoisomerase IIβ (TOP2β) inhibitor that is the only FDA-approved cardioprotectant for anthracycline therapy), angiotensin-converting enzyme inhibitors, angiotensin receptor blockers, beta-blockers, and sodium–glucose cotransporter-2 inhibitors. Although these approaches have demonstrated varying degrees of efficacy, substantial residual cardiovascular risk remains, particularly among long-term survivors [16,17,18,19].

### 1.3. Rationale for Nutritional Prevention

**Figure 1:**
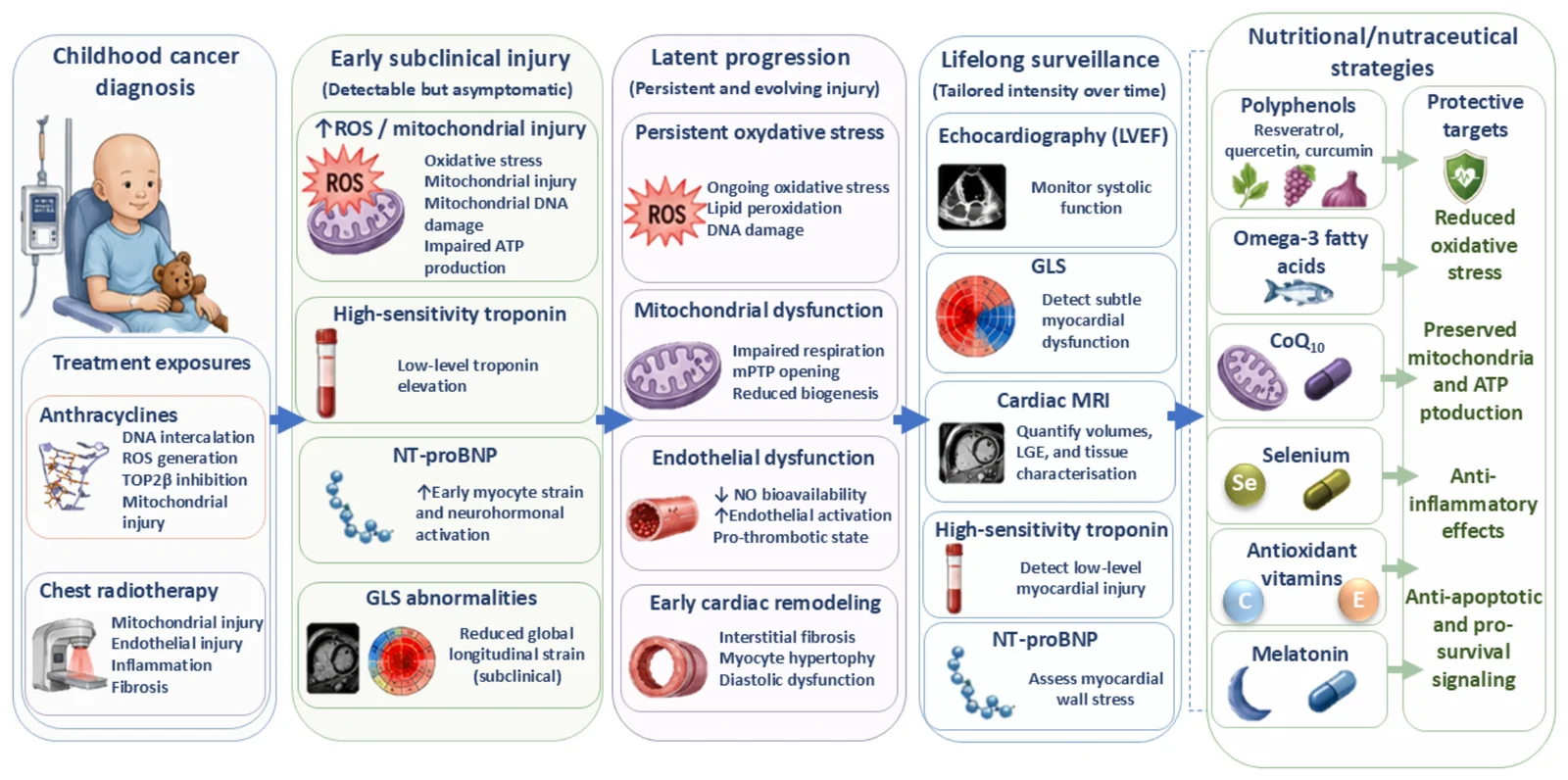
Life-course cardiovascular risk and precision nutritional prevention in pediatric cancer survivors. Schematic representation of cardiovascular risk progression in pediatric cancer survivors exposed to anthracyclines and chest radiotherapy. Initial treatment-related injury may cause early subclinical cardiac damage characterized by increased ROS production, mitochondrial injury, troponin elevation, NT-proBNP increase, and GLS abnormalities. During long-term survivorship, persistent oxidative stress, mitochondrial dysfunction, endothelial dysfunction, and early cardiac remodeling may progress silently before overt cardiovascular disease develops. Lifelong surveillance using echocardiography, GLS, cardiac MRI, high-sensitivity troponin, and natriuretic peptides enables early detection of myocardial injury. Nutritional and nutraceutical strategies, including polyphenols, omega-3 fatty acids, CoQ10, selenium, antioxidant vitamins, and melatonin, may provide complementary cardioprotection by reducing oxidative stress, preserving mitochondrial function and ATP production, limiting inflammation, and supporting anti-apoptotic and pro-survival signaling. Abbreviations: ATP—adenosine triphosphate; CoQ10—coenzyme Q10; DNA—deoxyribonucleic acid; GLS—global longitudinal strain; LGE—late gadolinium enhancement; LVEF—left ventricular ejection fraction; mPTP—mitochondrial permeability transition pore; MRI—magnetic resonance imaging; NO—nitric oxide; NT-proBNP—N-terminal pro-B-type natriuretic peptide; ROS—reactive oxygen species; TOP2β—topoisomerase II beta.

Growing interest has therefore been directed toward nutritional and nutraceutical cardioprotective strategies capable of targeting the molecular mechanisms underlying treatment-induced cardiac injury. Unlike many pharmacological interventions that focus on downstream manifestations of cardiotoxicity, nutritional compounds may modulate upstream pathogenic pathways, including OS, mitochondrial dysfunction, inflammation, and epigenetic dysregulation (Figure 1) [20,21,22,23].

**Figure 2:**
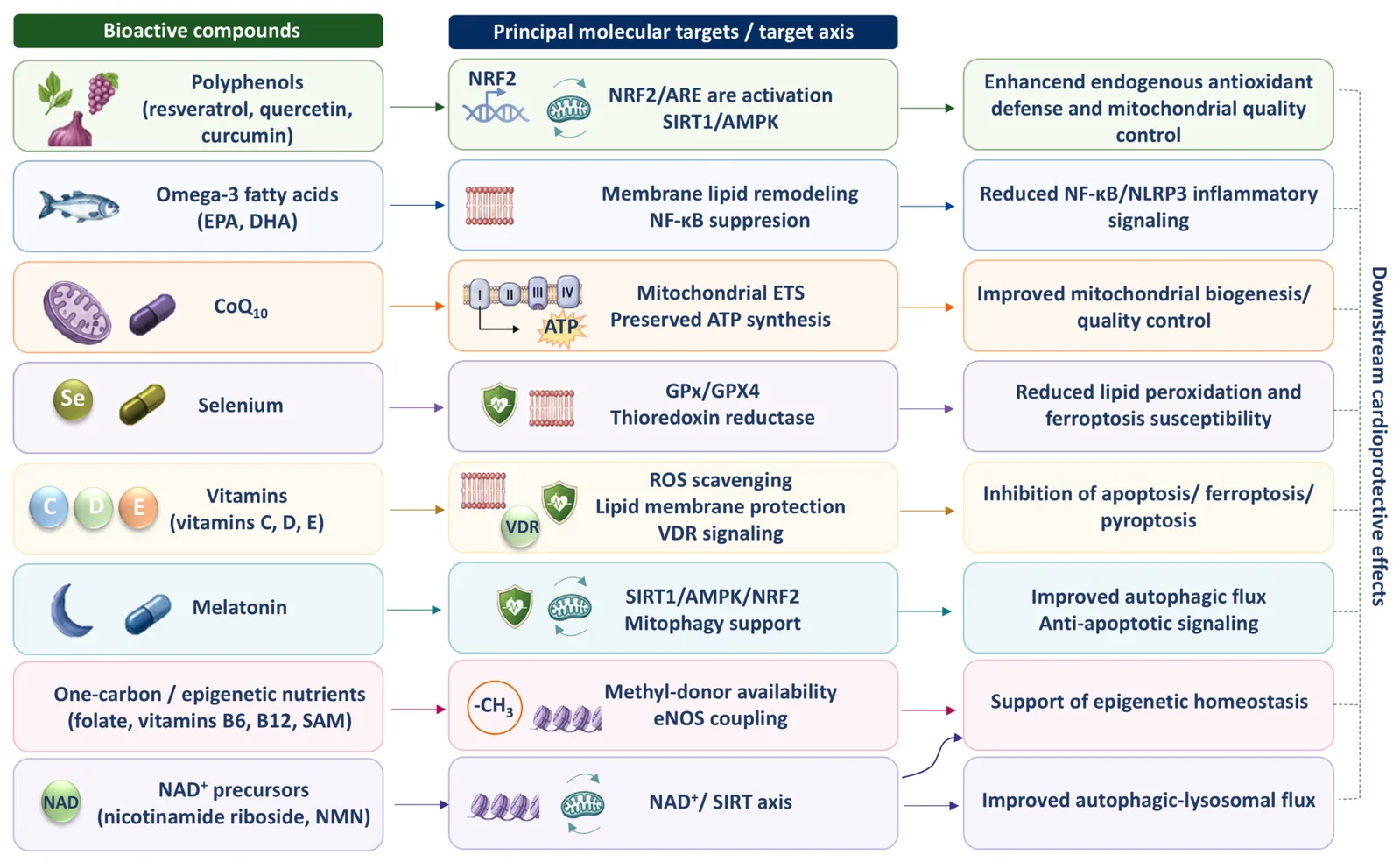
Major bioactive nutritional compounds, their principal molecular targets, and downstream cardioprotective effects. Schematic overview of the major nutritional and nutraceutical bioactive compounds discussed in this review and their principal molecular targets relevant to cancer therapy-related cardioprotection. Polyphenols, omega-3 fatty acids, coenzyme Q10, selenium, antioxidant vitamins, melatonin, one-carbon/epigenetic nutrients, and NAD^+^ precursors act through partially overlapping molecular pathways involving NRF2/ARE, SIRT1/AMPK, membrane lipid remodeling, mitochondrial electron transport and ATP synthesis, GPx/GPX4 and thioredoxin reductase activity, ROS scavenging, VDR signaling, mitophagy, methyl-donor availability, eNOS coupling, and NAD^+^/SIRT-dependent regulation. These mechanisms may enhance endogenous antioxidant defenses, preserve mitochondrial quality control and bioenergetics, suppress NF-κB/NLRP3-mediated inflammatory signaling, reduce lipid peroxidation and ferroptotic susceptibility, attenuate apoptosis and pyroptosis, improve autophagic–lysosomal flux, and support epigenetic homeostasis. Collectively, these interconnected effects may contribute to downstream cardioprotection during cancer therapy. The curved arrow between the one-carbon/epigenetic and NAD^+^-dependent pathways indicates mechanistic crosstalk between nutrient-sensitive epigenetic regulation and cellular metabolic signaling. Abbreviations: AMPK—AMP-activated protein kinase; ARE—antioxidant response element; ATP—adenosine triphosphate; CoQ10—coenzyme Q10; DHA—docosahexaenoic acid; eNOS—endothelial nitric oxide synthase; EPA—eicosapentaenoic acid; ETS—electron transport system; GPx—glutathione peroxidase; NAD^+^—nicotinamide adenine dinucleotide; NF-κB—nuclear factor kappa B; NLRP3—NLR family pyrin domain containing 3; NMN—nicotinamide mononucleotide; NRF2—nuclear factor erythroid 2-related factor 2; ROS—reactive oxygen species; SAM—S-adenosylmethionine; SIRT1—sirtuin 1; VDR—vitamin D receptor.

Nutritional interventions offer several theoretical advantages: multi-targeted mechanisms addressing OS, inflammation, and mitochondrial dysfunction; favorable safety profiles with minimal adverse effects; potential for long-term preventive use; and accessibility and acceptability to patients [24]. Emerging preclinical evidence supports the cardioprotective potential of dietary bioactive compounds and nutraceuticals, including polyphenols, omega-3 polyunsaturated fatty acids, coenzyme Q10, selenium, melatonin, and vitamins (Figure 2) [22,25]. These agents may preserve mitochondrial integrity, enhance endogenous antioxidant defenses, and activate cytoprotective signaling pathways such as nuclear factor erythroid 2-related factor 2 (Nrf2, a transcription factor that regulates expression of antioxidant, detoxification, and cellular defense systems) and Sirtuin 1 (SIRT1, an enzyme that regulates physiological processes by modifying gene expression through histone deacetylation), thereby suppressing inflammatory responses and attenuating oxidative injury induced by chemotherapy and radiotherapy [26,27].

Consequently, nutrition-based cardioprotection has emerged as a promising complementary approach within the evolving field of cardio-oncology. A better understanding of the molecular interactions between nutrition, OS, and cancer therapy-related cardiac injury may ultimately contribute to the development of more effective preventive strategies capable of improving both cardiovascular outcomes and quality of life among cancer survivors. Advances in nutrigenomics and precision nutrition are increasingly revealing how dietary factors interact with genetic and epigenetic determinants of cardiotoxicity, opening new opportunities for individualized prevention strategies [28,29]. However, clinical translation has been hampered by methodological heterogeneity, variable bioavailability, lack of standardized dosing, and concerns about potential interference with cancer treatment efficacy [30,31].

Recent reviews have addressed selected nutritional approaches to mitigate anticancer therapy-associated toxicity but have largely focused on adult populations, individual micronutrients, specific malignancies, or multiorgan toxicities. The present narrative review offers a broad mechanistic and translational perspective by integrating nutritional cardioprotection with redox imbalance, mitochondrial dysfunction, regulated cell death, epigenetic remodeling, and gut–heart signaling. Particular emphasis is placed on pediatric cancer survivors and on differentiating direct pediatric clinical evidence from adult and preclinical findings. Overall, this review synthesizes current evidence on the nutritional prevention of oxidative stress-induced cardiotoxicity, highlighting underlying molecular mechanisms, experimental and clinical data, and key translational challenges.

A structured literature search was performed to support this narrative review and minimize selective citation. PubMed/MEDLINE, Scopus, and Web of Science were searched from database inception to 12 August 2026. Search terms were combined using Boolean operators and included terms related to cancer therapy-associated cardiac injury (“cardiotoxicity”, “anthracycline”, “doxorubicin”, “radiotherapy”, “cardiomyopathy”, and “cardio-oncology”), oxidative and mitochondrial mechanisms (“oxidative stress”, “reactive oxygen species”, “antioxidant paradox”, “mitochondrial dysfunction”, “ferroptosis”, “pyroptosis”, and “epigenetics”), and nutritional interventions (“nutrition”, “nutraceutical”, “polyphenol”, “resveratrol”, “quercetin”, “curcumin”, “omega-3”, “coenzyme Q10”, “selenium”, “vitamin C”, “vitamin E”, “vitamin D”, “melatonin”, “folate”, “S-adenosylmethionine”, “NAD^+^”, “nicotinamide riboside”, “nicotinamide mononucleotide”, “gut microbiota”, “functional food”, “Mediterranean diet”, “Mediterranean dietary pattern”, “diet quality”, “whole diet”, “healthy eating”, and “Healthy Eating Index”). Additional searches specifically combined these terms with “child”, “pediatric”, “childhood cancer survivor”, “leukemia”, and “acute lymphoblastic leukemia”.

Priority was given to original experimental studies, randomized and controlled clinical trials, prospective clinical studies, systematic reviews and meta-analyses, and recent authoritative guidelines or consensus documents. Studies directly involving pediatric patients receiving anthracyclines were specifically identified and distinguished from adult clinical studies and preclinical evidence. Reference lists of relevant articles were also screened to identify additional eligible publications. Because the present work was designed as a narrative rather than a systematic review, no formal meta-analysis or GRADE assessment was undertaken; instead, the translational maturity of each nutritional intervention was interpreted according to the highest level of available evidence.

## 2. Mechanisms of Oxidative Stress-Induced Cardiotoxicity of Anthracyclines

**Figure 3:**
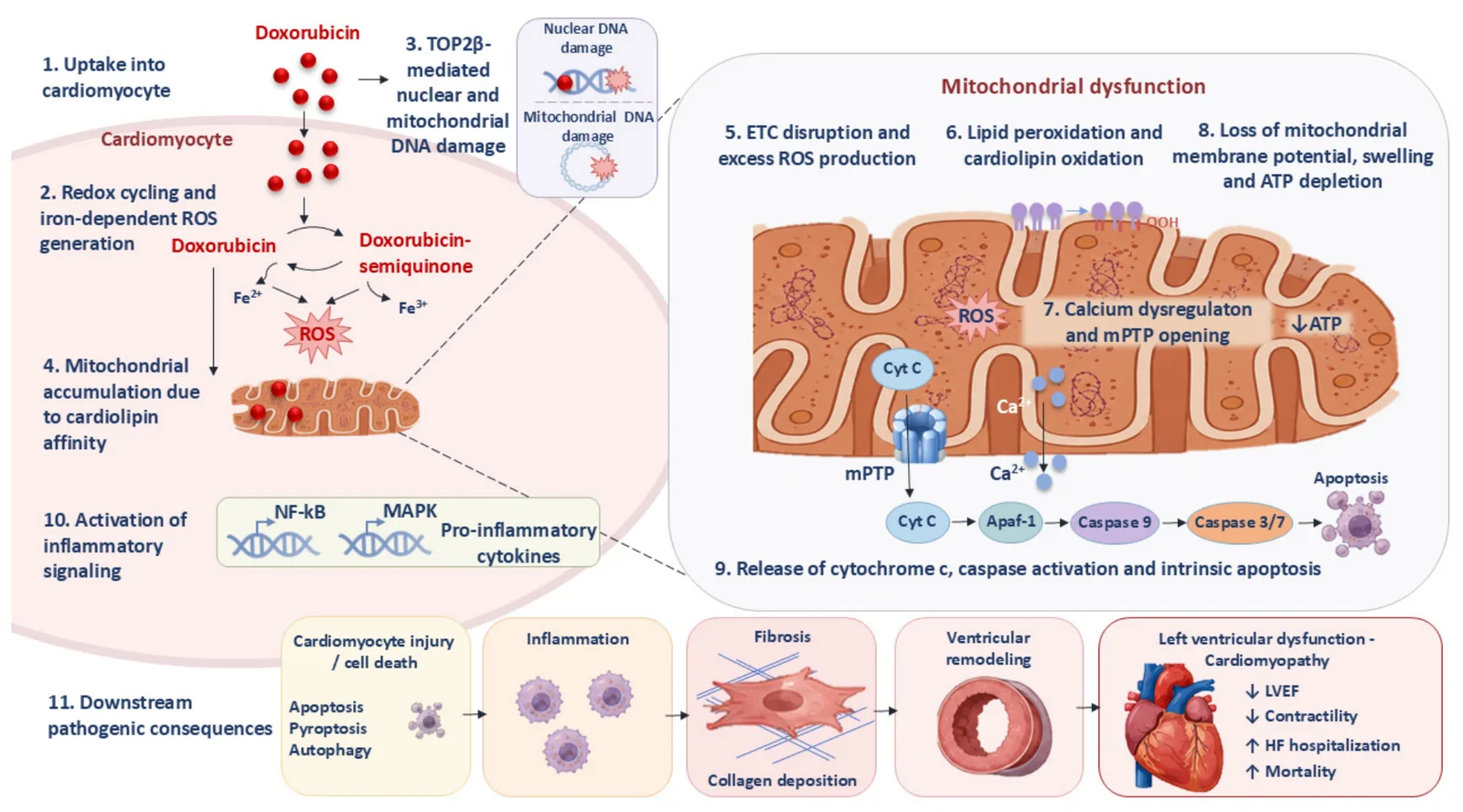
Doxorubicin-induced oxidative stress and mitochondrial dysfunction in the development of cardiomyopathy. Schematic overview of the main molecular mechanisms by which DOX promotes cardiomyopathy. After entering cardiomyocytes, DOX undergoes redox cycling and iron-dependent ROS generation and preferentially accumulates in mitochondria because of its affinity for cardiolipin. DOX also induces TOP2β-mediated nuclear and mitochondrial DNA damage. At the mitochondrial level, it disrupts the electron transport chain, increases mitochondrial ROS production, promotes lipid peroxidation and cardiolipin oxidation, impairs calcium homeostasis, and triggers opening of the mitochondrial permeability transition pore. These events cause loss of mitochondrial membrane potential, ATP depletion, cytochrome c release, and activation of intrinsic apoptosis. In parallel, inflammatory signaling is activated, further contributing to cardiomyocyte injury, inflammation, fibrosis, and ventricular remodeling, ultimately leading to left ventricular dysfunction, cardiomyopathy, and heart failure. Abbreviations Apaf-1—apoptotic protease activating factor 1; ATP—adenosine triphosphate; Cyt c—cytochrome c; DNA—deoxyribonucleic acid; DOX—doxorubicin; ETC—electron transport chain; HF—heart failure; LVEF—left ventricular ejection fraction; MAPK—mitogen-activated protein kinase; mPTP—mitochondrial permeability transition pore; NF-κB—nuclear factor kappa B; ROS—reactive oxygen species; TOP2β—topoisomerase II beta.

AIC involves complex, interconnected molecular mechanisms that converge on OS, mitochondrial dysfunction, and cardiomyocyte death. Understanding these pathways is essential for rational design of cardioprotective interventions (Figure 3). The quinone structure of anthracyclines undergoes one-electron reduction by NADPH-dependent reductases, generating highly unstable semiquinone radicals that rapidly react with molecular oxygen to produce superoxide anion (O_2_•^−^) [4]. This redox cycling continuously regenerates the parent quinone while producing ROS, creating a futile cycle of OS. Superoxide dismutase (SOD) converts O_2_•^−^ to hydrogen peroxide (H_2_O_2_), which can be further reduced to highly reactive hydroxyl radicals (•OH) via Fenton chemistry in the presence of inadequate sequestered ferrous iron as a catalyst [5].

Cardiomyocytes are particularly vulnerable to oxidative injury due to high mitochondrial density and metabolic activity (reflecting their high energetic demand, adult mammalian cardiomyocytes devote approximately 30–40% of their intracellular volume to mitochondria); relatively low expression of antioxidant enzymes (catalase and glutathione peroxidase (GPx)) compared to other tissues; and limited regenerative capacity [15]. Consequently, the accumulation of ROS leads to lipid peroxidation of cellular and mitochondrial membranes, generating toxic aldehydes such as 4-hydroxynonenal and malondialdehyde (MDA) that propagate oxidative damage [32,33].

AIC is largely mediated through the inhibition of TOP2β, which plays a crucial role in regulating DNA structure during transcriptional and replicative processes [34]. Unlike the TOP2α isoform targeted for anticancer effects, TOP2β is highly expressed in post-mitotic cardiomyocytes. The interaction of anthracyclines with TOP2β initiates a cascade characterized by DNA double-strand break formation, activation of p53- and ataxia–telangiectasia mutated (ATM)-mediated stress responses, and transcriptional suppression of genes governing mitochondrial biogenesis and redox balance [5]. Specifically, TOP2β inhibition suppresses expression of peroxisome proliferator-activated receptor gamma coactivator 1-alpha (PGC-1α), a master regulator of mitochondrial biogenesis, and downstream targets including mitochondrial transcription factor A (TFAM) and nuclear respiratory factors (NRF1/2) [35]. This transcriptional repression impairs mitochondrial turnover and repair, exacerbating oxidative injury. The cardioprotective effects of dexrazoxane are partially mediated through catalytic inhibition of TOP2β, which limits the formation of cleavable TOP2β–DNA complexes [36].

Anthracyclines possess high affinity for iron, forming anthracycline–iron complexes that catalyze Fenton reactions, generating highly reactive hydroxyl radicals from hydrogen peroxide [5]. This iron-dependent mechanism amplifies OS and contributes to lipid peroxidation, protein oxidation, and DNA damage. Cardiomyocytes are particularly susceptible to iron-catalyzed oxidative injury owing to their high metabolic demands, substantial mitochondrial content, and limited capacity for iron export, which collectively favor intracellular iron accumulation [37]. Dexrazoxane is metabolized intracellularly into an EDTA-like iron-chelating compound that reduces the pool of redox-active iron, thereby limiting anthracycline–iron complex formation, hydroxyl radical generation, and oxidative cardiac injury [33]. Although dexrazoxane remains the only approved cardioprotective agent for anthracycline-treated patients, its clinical administration has been limited so far by historical concerns regarding hematologic toxicity, secondary malignancies, and potential effects on tumor response, highlighting the continued need for safe and effective cardioprotective strategy development [38].

Beyond direct ROS generation, anthracyclines activate pro-inflammatory signaling cascades, including nuclear factor-kappa B (NF-κB), mitogen-activated protein kinases (MAPKs), and NLR family pyrin domain containing 3 (NLRP3) inflammasome, which amplify OS and promote cardiomyocyte apoptosis [5]. Inflammatory cytokines (TNF-α, IL-1β, and IL-6) further exacerbate cardiac injury through paracrine effects on endothelial cells, fibroblasts, and immune cells, contributing to cardiac remodeling and fibrosis [15].

## 3. Radiotherapy-Induced Cardiotoxicity and Oxidative Stress

Radiotherapy remains a cornerstone in the treatment of numerous thoracic malignancies, including breast cancer, Hodgkin lymphoma, lung cancer, and esophageal cancer. Despite substantial advances in radiation delivery techniques, inadvertent cardiac exposure remains an important clinical concern, particularly among long-term cancer survivors. Radiotherapy-induced heart disease represents a significant late complication of thoracic radiotherapy, with manifestations including pericarditis, myocardial fibrosis, coronary artery disease, valvular dysfunction, and conduction abnormalities [7]. The latency period for radiotherapy-induced heart disease ranges from months to decades, with risk proportional to radiation dose, volume of heart exposed, and presence of cardiovascular risk factors [39]. Unlike AIC, which primarily targets cardiomyocytes, radiotherapy-induced heart disease is characterized by extensive vascular injury, endothelial dysfunction, chronic inflammation, and progressive fibrosis affecting multiple cardiac structures [7].

Ionizing radiation induces cardiotoxicity through multiple mechanisms converging on OS. Direct ionization of water molecules generates reactive oxygen species (ROS), producing an acute oxidative burst, whereas radiation-induced mitochondrial dysfunction; NADPH oxidase activation; and increased formation of RNS, including nitric oxide (NO•) and peroxynitrite (ONOO^−^), sustain chronic oxidative and nitrosative stress [7]. Mitochondria represent both a major source and target of radiation-induced OS. Radiation-mediated disruption of the electron transport chain promotes electron leakage and persistent ROS generation, resulting in mitochondrial DNA damage, impaired ATP production, altered calcium handling, and activation of cell death pathways. Importantly, OS may persist long after completion of radiotherapy, contributing to the chronic and progressive nature of radiotherapy-induced heart disease. Experimental studies have demonstrated sustained increases in myocardial ROS production months after radiation exposure, suggesting that OS is not merely an acute event but rather a long-term pathological process [40].

The vascular endothelial cells are among the most radiosensitive cell type of the cardiovascular system and represent a primary target of radiation-induced injury. Endothelial cells exposed to ionizing radiation exhibit increased OS, DNA damage, mitochondrial dysfunction, and impaired nitric oxide bioavailability. Reduced nitric oxide production together with increased ROS and RNS generation results in endothelial dysfunction, characterized by impaired vasodilation, enhanced leukocyte adhesion, increased vascular permeability, and a prothrombotic phenotype [40]. Radiation-induced endothelial injury triggers upregulation of adhesion molecules, including vascular cell adhesion molecule-1 (VCAM-1), intracellular adhesion molecule-1 (ICAM-1), and E-selectin, facilitating recruitment and activation of inflammatory cells within the vascular wall [41]. Concurrently, endothelial damage promotes platelet activation, fibrin deposition, and intimal hyperplasia, thereby accelerating atherosclerotic processes and increasing risk of coronary artery disease. Endothelial injury promotes capillary rarefaction and subsequent microvascular dysfunction, and tissue hypoxia, creating a pro-fibrotic microenvironment. Activation of transforming growth factor-beta 1 (TGF-β1) signaling promotes differentiation of cardiac fibroblasts to myofibroblasts, excessive collagen deposition, and progressive myocardial fibrosis [7].

Accumulating evidence from preclinical studies indicates that antioxidant and anti-inflammatory interventions, including statins, angiotensin-converting enzyme inhibitors, and naturally derived antioxidants, can attenuate radiotherapy-induced heart disease by scavenging ROS, suppressing pro-inflammatory signaling pathways, and limiting myocardial fibrosis [7,42,43,44]. Lisinopril, an ACE inhibitor, was shown to mitigate radiation-induced mitochondrial defects in rat heart and blood cells, preserving mitochondrial membrane potential and reducing oxidative damage [45]. However, clinical validation of these interventions for radiotherapy-induced heart disease prevention remains limited. Integrated multimodal surveillance, incorporating speckle-tracking echocardiography; cardiac MRI; and circulating biomarkers such as high-sensitivity cardiac troponin T, NT-proBNP, and circulating endothelial cells, may facilitate the early identification of subclinical cardiotoxicity and enable the timely initiation of cardioprotective interventions [46]. However, optimal screening protocols and intervention thresholds require further investigation.

## 4. Oxidative Stress, Cardiac Remodeling, and Heart Failure Development

**Figure 4:**
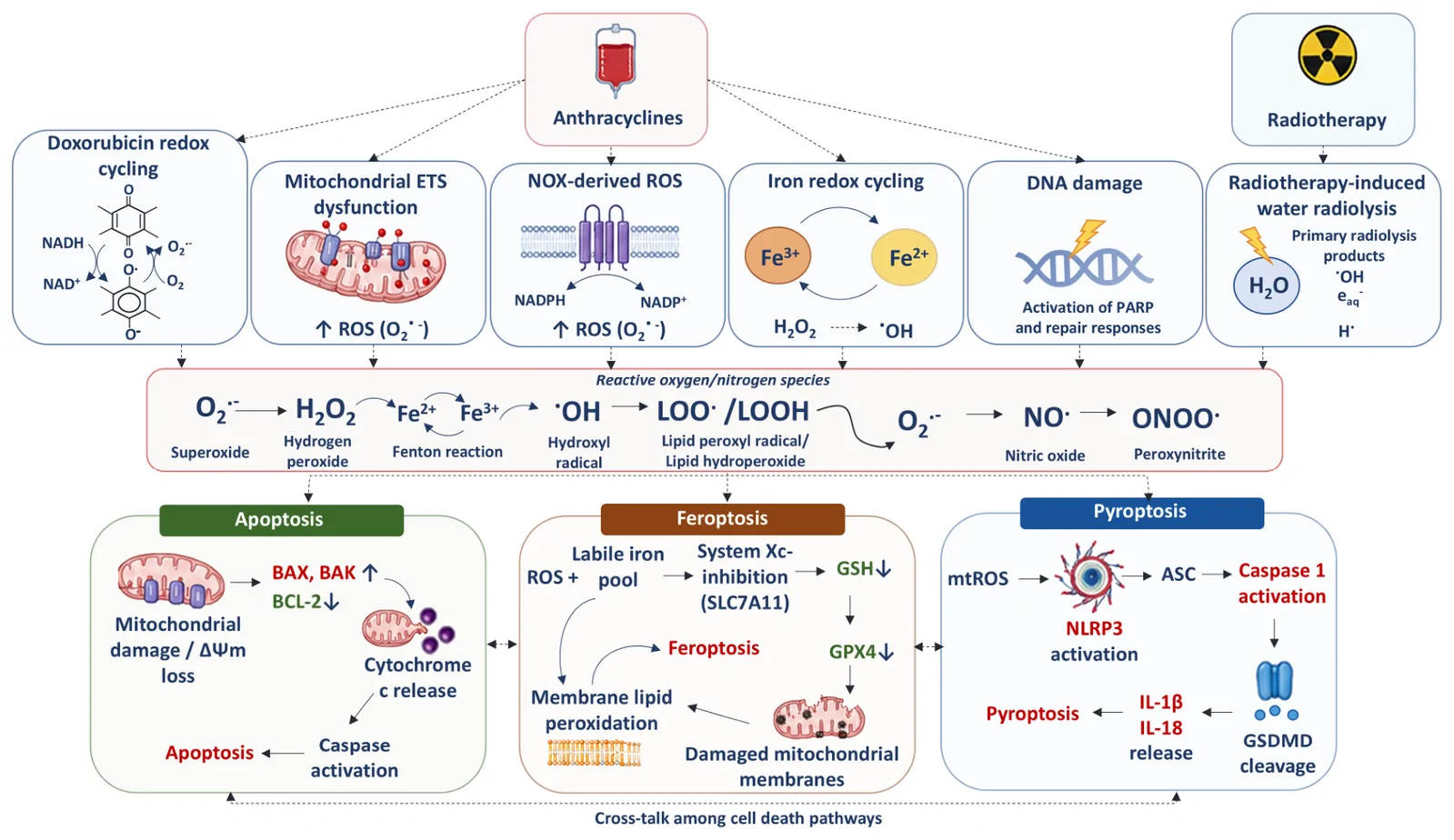
Integrated schematic representation of the major oxidative and redox-dependent mechanisms linking anthracycline treatment and radiotherapy to regulated cardiomyocyte death. Anthracyclines promote reactive oxygen species generation through doxorubicin redox cycling, mitochondrial electron transport system dysfunction, NOX-dependent ROS production, iron redox cycling, and DNA damage, whereas radiotherapy additionally induces water radiolysis with the formation of highly reactive primary species. These processes converge on excessive generation of reactive oxygen and nitrogen species, including superoxide, hydrogen peroxide, hydroxyl radicals, lipid peroxyl radicals/lipid hydroperoxides, nitric oxide, and peroxynitrite. Oxidative stress subsequently activates interconnected cell-death pathways. Apoptosis is mediated predominantly through mitochondrial membrane depolarization, altered BAX/BAK–BCL-2 balance, cytochrome c release, and caspase activation. Ferroptosis is driven by expansion of the labile iron pool, inhibition of system Xc^−^, glutathione depletion, GPX4 impairment, and membrane lipid peroxidation. Pyroptosis is promoted by mitochondrial ROS-dependent NLRP3 inflammasome activation, ASC recruitment, caspase-1 activation, GSDMD cleavage, and release of IL-1β and IL-18. Dashed bidirectional arrows indicate mechanistic crosstalk among apoptosis, ferroptosis, and pyroptosis, emphasizing that these pathways are not functionally isolated but form an interconnected network of oxidative stress-driven cardiomyocyte injury. Abbreviations: ASC—apoptosis-associated speck-like protein containing a CARD; BAK—BCL-2 antagonist/killer 1; BAX—BCL-2-associated X protein; BCL-2—B-cell lymphoma 2; DNA—deoxyribonucleic acid; e_aq_^−^—hydrated electron; ETS—electron transport system; GPX4, glutathione peroxidase 4; GSDMD—gasdermin D; GSH—glutathione; H•—hydrogen atom; IL-1β—interleukin-1 beta; IL-18—interleukin-18; LOO•—lipid peroxyl radical; LOOH—lipid hydroperoxide; mtROS—mitochondrial reactive oxygen species; NAD^+^—nicotinamide adenine dinucleotide; NADH—reduced nicotinamide adenine dinucleotide; NADP^+^—nicotinamide adenine dinucleotide phosphate; NADPH—reduced nicotinamide adenine dinucleotide phosphate; NLRP3—NLR family pyrin domain containing 3; NO•—nitric oxide; NOX, NADPH oxidase; O_2_•^−^—superoxide anion radical; •OH—hydroxyl radical; ONOO^−^—peroxynitrite; PARP—poly(ADP-ribose) polymerase; ROS—reactive oxygen species; SLC7A11—solute carrier family 7 member 11; ΔΨm—mitochondrial membrane potential.

OS represents a central pathogenic axis linking early molecular injury to late structural and functional deterioration of the myocardium in cancer therapy-related cardiotoxicity. Excessive generation of ROS and RNS initiates a complex network of cellular responses involving mitochondrial dysfunction, impaired calcium handling, DNA damage, inflammatory activation, extracellular matrix remodeling, and cardiomyocyte death. These interconnected oxidative stress-driven mechanisms converge on several forms of regulated cardiomyocyte death, most notably apoptosis, ferroptosis, and pyroptosis, as summarized in Figure 4. These processes are particularly relevant in AIC and radiation-induced heart disease, where oxidative damage may persist long after treatment completion and contribute to progressive cardiac remodeling and heart failure development (Figure 1) [47,48,49].

The transition from subcellular injury to clinically evident ventricular dysfunction is not abrupt but evolves through a continuum of maladaptive responses. Initially, OS impairs mitochondrial bioenergetics and activates stress-sensitive signaling pathways. Subsequently, cardiomyocyte apoptosis, ferroptosis, inflammatory cell recruitment, fibroblast activation, and extracellular matrix deposition promote adverse myocardial remodeling. Over time, these changes result in ventricular dilatation or stiffening, impaired systolic and/or diastolic function, reduced cardiac reserve, and eventually overt heart failure [48,50].

### 4.1. Apoptosis

Apoptosis is one of the most extensively characterized mechanisms of cardiomyocyte loss in AIC. DOX promotes apoptosis through both intrinsic mitochondrial and extrinsic death receptor-mediated pathways. The intrinsic pathway is primarily triggered by OS, mitochondrial DNA damage, calcium overload, and loss of mitochondrial membrane potential. These events lead to mitochondrial outer membrane permeabilization; cytochrome c release; apoptosome formation; caspase-9 activation; and downstream activation of executioner caspases, particularly caspase-3 (Figure 3) [47,51]. At the molecular level, anthracyclines alter the balance between pro-apoptotic and anti-apoptotic B-cell lymphoma 2 (BCL-2) family proteins. Increased expression of BAX and BAK, together with reduced BCL-2 activity, facilitates mitochondrial membrane permeabilization and irreversible commitment to apoptosis [47]. In addition, DOX-induced DNA damage activates p53-dependent transcriptional programs, thereby promoting mitochondrial apoptotic signaling and cell-cycle arrest in cardiomyocytes [52].

Radiotherapy-induced cardiac injury similarly involves apoptotic signaling, particularly in endothelial cells, cardiomyocytes, and vascular smooth muscle cells. Ionizing radiation induces DNA double-strand breaks, mitochondrial dysfunction, and ROS/RNS accumulation, thereby activating p53, MAPK, and NF-κB-related pathways that may culminate in apoptosis. Although apoptosis may initially serve to eliminate irreversibly damaged cells, excessive or persistent activation contributes to cumulative cardiomyocyte loss, capillary rarefaction, impaired contractile reserve, and subsequent myocardial remodeling [7,47].

Importantly, apoptosis is closely integrated with other forms of regulated cell death, including ferroptosis, pyroptosis, necroptosis, and autophagy-related cell death. This overlap suggests that cardiotoxicity should not be conceptualized as a single-pathway phenomenon but rather as the consequence of interacting cell death programs driven by OS and mitochondrial dysfunction [53,54].

### 4.2. Ferroptosis

Ferroptosis has recently emerged as a particularly important mechanism of anthracycline-induced cardiac injury. It is a regulated, iron-dependent form of cell death characterized by excessive lipid peroxidation, depletion of glutathione, inactivation of glutathione peroxidase 4 (GPX4), and accumulation of oxidized phospholipids within cellular and mitochondrial membranes [55,56].

DOX promotes ferroptosis through several converging mechanisms. First, it disrupts intracellular iron homeostasis by increasing the labile iron pool and compromising ferritin-mediated iron sequestration. Second, DOX enhances mitochondrial ROS generation, which accelerates lipid peroxidation. Third, anthracycline exposure impairs cellular antioxidant defenses, particularly by inhibiting the cystine/glutamate antiporter system Xc^−^, depleting intracellular glutathione, and reducing GPX4 activity. Together, these alterations generate a biochemical environment favorable for ferroptotic cardiomyocyte death [55,56,57].

Several ferroptosis-related regulatory pathways have been implicated in DOX-induced cardiotoxicity, including Nrf2/Keap1, GPX4, heme oxygenase-1, AMP-activated protein kinase (AMPK), mitochondrial ferritin, and ferroportin signaling. The Nrf2 pathway is particularly relevant because it coordinates antioxidant defense, iron metabolism, and lipid peroxide detoxification. However, Nrf2 signaling may exert context-dependent effects depending on the balance between antioxidant adaptation and heme oxygenase-1-mediated iron release [55,57].

The relevance of ferroptosis is supported by experimental studies showing that pharmacological or genetic inhibition of ferroptotic pathways attenuates DOX-induced myocardial injury. Ferroptosis inhibitors, iron chelators, GPX4-preserving interventions, and natural compounds targeting lipid peroxidation have shown cardioprotective effects in preclinical models [55,58]. These findings provide a strong rationale for considering ferroptosis as a therapeutic target and help explain why nutritional compounds that modulate iron metabolism, glutathione homeostasis, Nrf2 signaling, and lipid peroxidation may exert cardioprotective effects.

### 4.3. Pyroptosis

Pyroptosis is an inflammatory form of regulated cell death that has recently emerged as an important contributor to cancer therapy-related cardiotoxicity. Unlike apoptosis, which is generally considered immunologically silent, pyroptosis is characterized by inflammasome activation, caspase-dependent cleavage of gasdermin proteins, formation of membrane pores, cell swelling, membrane rupture, and release of pro-inflammatory intracellular contents. This process links OS, mitochondrial dysfunction, innate immune activation, and adverse cardiac remodeling, making it highly relevant in both AIC and radiation-induced heart disease [47,54,59].

The canonical pyroptotic pathway is primarily mediated by activation of the nucleotide-binding oligomerization domain-like receptor family pyrin domain-containing 3 (NLRP3) inflammasome. In response to danger signals such as mitochondrial ROS, oxidized mitochondrial DNA, potassium efflux, lysosomal damage, and calcium dysregulation, NLRP3 assembles with the adaptor protein apoptosis-associated speck-like protein containing a CARD (ASC) and pro-caspase-1. This complex promotes caspase-1 activation, which subsequently cleaves pro-IL-1β, pro-IL-18, and gasdermin D (GSDMD). The N-terminal fragment of GSDMD forms pores in the plasma membrane, leading to pyroptotic cell death and amplification of local inflammatory signaling [59,60].

In DOX-induced cardiotoxicity, pyroptosis appears to be closely linked to OS and mitochondrial injury. DOX increases mitochondrial ROS generation, disrupts mitochondrial membrane potential, and promotes release of mitochondrial damage-associated molecular patterns, which can activate NLRP3 inflammasome signaling. Experimental studies have shown that DOX upregulates NLRP3, cleaved caspase-1, GSDMD-N, IL-1β, and IL-18 in cardiomyocytes, supporting the involvement of NLRP3/caspase-1/GSDMD-mediated pyroptosis in anthracycline-related myocardial injury [47,60,61].

Recent mechanistic studies further suggest that NADPH oxidase 4 (NOX4)-derived ROS may aggravate DOX-induced cardiomyocyte pyroptosis by activating the NLRP3 inflammasome. This finding is particularly relevant because it positions ROS not only as a direct mediator of oxidative damage but also as an upstream trigger of inflammatory cell death and cytokine release [61]. Similarly, inhibition of NLRP3 inflammasome activation has been shown to attenuate DOX-induced myocardial injury, improve cardiac function, and reduce inflammatory damage in experimental models [60,62]. GSDMD has also been identified as a direct mediator of DOX-induced cardiomyocyte pyroptosis. Experimental evidence indicates that GSDMD activation contributes to mitochondrial damage, inflammatory cytokine release, and structural cardiac injury following DOX exposure. These findings suggest that the GSDMD axis may represent a potential therapeutic target for limiting inflammatory cardiomyocyte death in AIC [63].

Pyroptosis is also relevant in radiation-induced cardiac injury. Ionizing radiation induces mitochondrial dysfunction, ROS/RNS production, endothelial damage, and chronic inflammation, all of which may activate the NLRP3 inflammasome. Recent reviews have highlighted NLRP3 inflammasome signaling as a central mediator of radiation-induced cardiovascular injury, contributing to endothelial dysfunction, inflammatory cell recruitment, microvascular damage, myocardial fibrosis, and late cardiac remodeling [7,64]. In this context, pyroptosis may serve as an important mechanistic bridge between early radiation-induced oxidative injury and chronic inflammatory-fibrotic remodeling.

Pyroptosis may be particularly relevant to the progression of cardiotoxicity because it propagates myocardial injury beyond the initially affected cardiomyocytes. The release of the pro-inflammatory cytokines IL-1β and IL-18, together with extracellular ATP, high-mobility group box 1, and other damage-associated molecular patterns, promotes immune-cell recruitment, cardiac fibroblast activation, endothelial dysfunction, and extracellular matrix accumulation. Through these interconnected inflammatory and profibrotic responses, pyroptosis may contribute to the transition from acute oxidative myocardial injury to persistent inflammation, myocardial fibrosis, adverse ventricular remodeling, and ultimately heart failure. Several experimental interventions targeting pyroptotic signaling have shown cardioprotective effects. Natural compounds and phytochemicals, including calycosin, amentoflavone, astragaloside IV, and bardoxolone methyl, have been reported to attenuate DOX-induced cardiotoxicity by suppressing NLRP3 inflammasome activation, inhibiting caspase-1/GSDMD signaling, activating SIRT1 or Nrf2 pathways, and reducing inflammatory cytokine release [60,63,65,66,67]. These findings provide a strong mechanistic rationale for investigating nutrition-derived and nutraceutical interventions that modulate inflammasome activation, mitochondrial ROS, redox-sensitive signaling, and inflammatory cell death.

Collectively, pyroptosis should be considered an important component of OS-driven cardiotoxicity. By integrating mitochondrial injury, inflammasome activation, cytokine release, endothelial dysfunction, and fibro-inflammatory remodeling, pyroptosis provides a mechanistic link between early subcellular damage and late structural deterioration of the myocardium. Targeting the ROS/NLRP3/caspase-1/GSDMD axis may therefore represent a promising strategy for preventing or attenuating cancer therapy-related cardiac remodeling and heart failure development.

## 5. Mitochondrial Dysfunction and Cardiac Remodeling

Mitochondria are central to anthracycline and radiation-induced cardiotoxicity, serving as both primary targets and amplifiers of oxidative injury. Cardiomyocytes contain the highest mitochondrial density of any cell type, reflecting their enormous energy demands for continuous contractile function. Accumulating evidence suggests that mitochondrial injury represents not merely a consequence of OS but also an active driver of cardiac damage. Cancer therapies disrupt mitochondrial bioenergetics, impair ATP production, alter calcium homeostasis, induce mitochondrial DNA damage, and interfere with mitochondrial quality control mechanisms. These alterations collectively promote cardiomyocyte dysfunction, maladaptive remodeling, and progressive heart failure [68,69].

### 5.1. Mitochondrial ROS and Electron Transport Chain Disruption

Anthracyclines preferentially localize to mitochondria owing to their high affinity for cardiolipin, a phospholipid highly enriched in the inner mitochondrial membrane [33]. Cardiolipin binding disrupts electron transport chain function, particularly at Complex I and Complex III, leading to electron leakage and excessive superoxide generation [70]. Mitochondrial ROS production creates a vicious cycle: oxidative damage to ETC proteins further impairs electron transport, amplifying ROS generation and propagating mitochondrial dysfunction. Mitochondrial DNA (mtDNA) is particularly susceptible to oxidative injury because of its proximity to the electron transport chain, absence of protective histones, and relatively limited DNA repair capacity. Excessive ROS generation induced by anthracyclines and radiation promotes mtDNA oxidation, strand breaks, deletions, and mutations, leading to impaired transcription of genes encoding critical components of the respiratory chain [69]. DOX has been shown to directly accumulate within mitochondria and interact with mtDNA, exacerbating mitochondrial dysfunction and impairing mitochondrial biogenesis. Damage to mtDNA contributes to defective oxidative phosphorylation, reduced ATP production, and increased ROS generation, thereby amplifying mitochondrial injury and cardiomyocyte dysfunction [68,71,72].

Excessive mitochondrial ROS promote cardiolipin peroxidation, disrupting its essential roles in preserving mitochondrial membrane integrity, maintaining cristae architecture, and stabilizing electron transport system (ETS) supercomplexes [70,73]. Oxidative modification of cardiolipin facilitates the release of cytochrome c from the inner mitochondrial membrane into the cytosol, thereby initiating caspase-dependent intrinsic apoptosis. Moreover, mitochondrial ROS promote mitochondrial permeability transition pore (mPTP) opening, leading to mitochondrial swelling, membrane depolarization, bioenergetic failure, and depletion of intracellular ATP stores (Figure 3) [71].

### 5.2. Mitochondrial Dynamics: Fission, Fusion, and Mitophagy

Mitochondrial quality control relies on the coordinated regulation of mitochondrial fission; fusion; and mitophagy, the selective removal of damaged mitochondria. Anthracycline exposure disrupts these homeostatic mechanisms by promoting excessive mitochondrial fission and impairing mitophagy clearance. The resulting accumulation of dysfunctional mitochondria enhances ROS production and contributes to progressive cardiomyocyte injury [74,75]. Namely, DOX disrupts mitochondrial dynamics by promoting a shift toward mitochondrial fission, characterized by increased expression and activation of fission-related proteins such as dynamin-related protein 1 (Drp1) and fission protein 1 (Fis1), together with reduced levels of fusion mediators including mitofusin 2 (Mfn2) and optic atrophy 1 (OPA1). These alterations ultimately lead to mitochondrial fragmentation and dysfunction [76]. Excessive mitochondrial fragmentation is associated with impaired oxidative phosphorylation and reduced ATP production, increased ROS production, and heightened susceptibility to mitochondria-mediated apoptotic signaling. Astaxanthin, a carotenoid antioxidant, was shown to mitigate DOX-induced cardiotoxicity by restoring mitochondrial dynamics, upregulating *Mfn2* and *OPA1* while reducing *Drp1* and *Fis1* expression [74,77].

Mitophagy is a specialized form of autophagy that maintains mitochondrial quality by selectively eliminating damaged mitochondria, primarily through the PINK1/Parkin signaling pathway. Anthracycline exposure may disrupt mitophagy flux, resulting in either insufficient removal of dysfunctional mitochondria or excessive mitochondrial degradation, both of which can aggravate mitochondrial dysfunction and cardiomyocyte injury [71,78]. Astaxanthin was found to regulate PINK1/Parkin-mediated mitophagy, promoting clearance of damaged mitochondria while preserving healthy mitochondrial populations [74].

### 5.3. Calcium Dysregulation and Membrane Depolarization

Mitochondria contribute to cytosolic Ca^2+^ buffering and the maintenance of calcium homeostasis required for normal excitation–contraction coupling. Anthracycline exposure disrupts calcium handling by altering the function of key regulatory proteins, including ryanodine receptors, sarco/endoplasmic reticulum Ca^2+^-ATPase (SERCA), and the mitochondrial calcium uniporter. The resulting mitochondrial Ca^2+^ overload promotes opening of the mPTP, dissipation of the mitochondrial membrane potential, impairment of oxidative phosphorylation and ATP synthesis, and activation of mitochondria-mediated apoptotic pathways [79]. Simvastatin co-treatment was shown to reduce DOX-induced mitochondrial calcium overload and membrane depolarization in human cardiomyocytes, preserving mitochondrial function [80].

Importantly, disturbances in calcium homeostasis have been observed not only in AIC but also following radiotherapy and targeted anticancer therapies, suggesting that calcium dysregulation may represent a convergent mechanism contributing to treatment-related cardiac injury [81]. Collectively, mitochondrial dysfunction constitutes a central pathogenic nexus through which multiple cardiotoxic mechanisms converge. Accordingly, nutritional interventions that preserve mitochondrial structural and functional integrity, promote mitochondrial biogenesis, and maintain mitochondrial quality-control pathways may represent promising strategies for cardioprotection.

## 6. Pediatric Cancer Survivors: Cardiovascular Risk and Long-Term Complications

Pediatric cancer survivors constitute a particularly vulnerable population with respect to long-term cardiovascular morbidity and mortality. Although approximately 80% of children diagnosed with cancer now survive for at least 5 years, survivors have an approximately eight-fold higher risk of cardiovascular mortality than age-matched individuals without a history of childhood cancer [82]. Anthracycline exposure during childhood confers a substantial lifelong risk of cardiotoxicity because of the cumulative dose-dependent nature of myocardial injury, the increased vulnerability of the developing heart, and the prolonged latency during which subclinical cardiac damage may progress to clinically overt dysfunction. This risk may be further amplified by somatic growth, pubertal and hormonal changes, and the progressive accumulation of conventional cardiovascular risk factors during adulthood [83,84].

Childhood and adolescence may represent a particularly important developmental window for preventive interventions because cardiac structure, metabolism, and regulatory programs remain highly adaptive during growth and maturation. Mitochondrial function and quality-control pathways undergo substantial developmental remodeling, and mitochondrial ROS are closely linked to cardiomyocyte maturation and cellular stress responses. This developmental plasticity may increase the long-term consequences of oxidative injury occurring during anticancer therapy. Consistent with this concept, recent studies in anthracycline-exposed childhood cancer survivors have identified persistent alterations in mitochondrial gene-expression pathways associated with subsequent cardiomyopathy [85]. Developmental plasticity also involves extensive transcriptional and epigenetic regulation. Importantly, differential DNA methylation patterns have been identified in anthracycline-exposed childhood cancer survivors who subsequently developed cardiomyopathy, providing clinical evidence that persistent epigenetic alterations may contribute to late cardiac injury. Nutritional status may intersect with these mechanisms through one-carbon metabolism and methyl-donor availability, nicotinamide adenine dinucleotide (NAD^+^)-dependent sirtuin activity, antioxidant defenses, and mitochondrial homeostasis. Therefore, nutritional prevention during childhood may theoretically influence not only acute redox injury but also longer-term metabolic and epigenetic programming [86]. Puberty represents an additional period of cardiovascular and metabolic adaptation, characterized by rapid somatic growth, changes in body composition and sex-hormone concentrations, and increasing hemodynamic demands. These developmental changes may interact with pre-existing subclinical myocardial injury acquired during cancer therapy and thereby influence the evolution of late cardiotoxicity. Consequently, nutritional strategies initiated during childhood or adolescence may be particularly relevant from a life-course perspective. However, this concept should currently be regarded as a strong biological and translational rationale rather than evidence of established clinical efficacy, since adequately powered pediatric nutritional intervention trials remain limited.

Among childhood cancer survivors, patients treated for ALL represent one of the largest and most extensively investigated survivor populations. Because anthracyclines remain a cornerstone of treatment protocols for pediatric leukemia, long-term survivors are particularly vulnerable to therapy-related cardiac injury (Figure 1). Survivors of childhood ALL, who are frequently exposed to anthracyclines as part of multi-agent chemotherapy, may experience a progressive decline in left ventricular function, with symptomatic heart failure reported in approximately 10–20% of patients within 20 years after treatment [1,82]. Recent analyses from the Childhood Cancer Survivor Study (CCSS), St. Jude Lifetime Cohort (SJLIFE), and PanCareSurFup consortium have demonstrated that the cumulative burden of cardiovascular disease continues to increase throughout adulthood, with survivors exhibiting accelerated cardiovascular aging and substantially higher rates of major adverse cardiovascular events than the general population [87,88].

Several treatment-related, demographic, and genetic factors have been associated with an increased risk of AIC in pediatric patients, including higher cumulative anthracycline exposure (>250 mg/m^2^); younger age at treatment, particularly before 5 years of age; female sex; concomitant chest radiotherapy; and genetic variants affecting drug metabolism, transport, and antioxidant defense pathways [89,90]. The American Heart Association recently issued a scientific statement emphasizing the need for lifelong cardiovascular surveillance in pediatric cancer survivors, with risk-stratified screening protocols based on treatment exposures [2]. Serial echocardiography remains the cornerstone of cardiotoxicity surveillance, allowing the detection of subclinical left ventricular impairment through the evaluation of systolic and diastolic function, including left ventricular ejection fraction (LVEF), global longitudinal strain (GLS), and diastolic filling parameters [91]. Although LVEF remains the most widely used parameter for cardiac surveillance, it is relatively insensitive to early myocardial injury and often declines only after significant structural and functional damage has occurred. In contrast, advanced imaging modalities, such as speckle-tracking echocardiography and cardiac MRI with parametric T1 and T2 mapping, can detect subclinical myocardial abnormalities, including fibrosis, edema, and inflammation, at earlier stages of cardiotoxicity [91]. Serum biomarkers provide a complementary approach to imaging-based surveillance of cardiotoxicity. Among these, cardiac troponins and natriuretic peptides have demonstrated utility in detecting subclinical myocardial injury and stratifying future cardiovascular risk. Notably, increases in troponin levels during or immediately following anthracycline therapy have been shown to predict later declines in cardiac function, supporting their role in identifying high-risk individuals for intensified monitoring and preventive treatment strategies (Figure 1) [92,93].

The cardiovascular effects of childhood cancer therapy may extend well beyond the pediatric period and persist throughout survivorship. In contrast to conventional cardiovascular disease, which most commonly becomes clinically apparent in middle or later adulthood, treatment-related cardiovascular injury may originate during childhood and progress subclinically over several decades before manifesting as overt cardiac disease [87]. Pharmacological intervention following the detection of asymptomatic left ventricular dysfunction, primarily with angiotensin-converting enzyme inhibitors and β-blockers, has yielded limited and inconsistent benefits in childhood cancer survivors, with no conclusive evidence that these secondary preventive therapies prevent progression to clinically overt heart failure [17]. These limitations underscore the importance of primary cardioprotection aimed at preventing or attenuating myocardial injury during cancer treatment. Dexrazoxane represents the most extensively studied cardioprotective agent in pediatric patients receiving anthracyclines and may be considered, particularly when high cumulative anthracycline exposure is anticipated. Although historical concerns regarding secondary malignant neoplasms and potential interference with antitumor efficacy initially restricted its use, accumulating evidence from long-term follow-up studies indicates that dexrazoxane provides sustained cardioprotection without a demonstrable adverse effect on cancer-related outcomes or the overall risk of second malignancies [94].

The cardiovascular burden among childhood cancer survivors extends beyond heart failure and encompasses a broad spectrum of late complications, including premature coronary artery disease, valvular heart disease, cardiac arrhythmias, cerebrovascular events, and vascular dysfunction [95]. In addition to treatment-related myocardial injury, survivors of childhood leukemia frequently develop cardiometabolic abnormalities, such as hypertension, obesity, insulin resistance, metabolic syndrome, and endothelial dysfunction, which may contribute to premature cardiovascular aging and further increase long-term cardiovascular risk [96,97,98]. Emerging evidence suggests that hypertension may develop at a substantially younger age in anthracycline-exposed survivors than in the general population and may represent an important modifiable risk factor for the subsequent development or progression of cardiac dysfunction [99].

Childhood cancer survivors who have undergone hematopoietic stem cell transplantation are at particularly high cardiovascular risk because of the combined effects of prior cardiotoxic treatment exposures, total-body irradiation, graft-versus-host disease, persistent systemic inflammation, and prolonged immunosuppressive therapy [95]. Accordingly, lifelong cardiovascular surveillance and proactive management of modifiable risk factors, including hypertension, dyslipidemia, diabetes mellitus, and obesity, are essential components of survivorship care and may improve long-term cardiovascular outcomes in this growing population [98]. Current international guidelines recommend lifelong, risk-adapted cardiovascular surveillance based on treatment-related exposures, cumulative anthracycline dose, radiation dose and field, and additional patient-specific risk factors [100]. However, conventional risk-stratification models may not fully capture interindividual susceptibility, as they generally provide limited consideration of genetic predisposition, epigenetic alterations, biological aging, and dynamically evolving cardiometabolic risk profiles [29,100]. These limitations underscore the need for more comprehensive preventive strategies that address the complex interactions among treatment-related injury, OS, biological aging, and acquired cardiovascular risk factors. Within this framework, nutritional interventions targeting OS, mitochondrial dysfunction, inflammation, and epigenetic dysregulation may offer a complementary approach to reducing long-term cardiovascular risk in childhood cancer survivors, although their clinical effectiveness requires further prospective validation.

## 7. Epigenetic Factors in Oxidative Stress-Induced Cardiotoxicity

OS represents one of the mechanisms by which environmental triggers (including xenobiotics and nutrition) can induce acute, chronic, or late-onset cardiotoxicity. OS modulates the epigenetic landscape contributing to the pathogenesis of cardiovascular diseases, as it is highlighted by results of numerous in vitro and in vivo experiments [101]. Epigenetic regulation, defined as heritable changes in gene expression without alterations in DNA sequence, has been proposed to mediate the interaction between environmental and genetic susceptibility factors, providing the direct environmental-to-genetic interface [102].

The novel concept of “redox epigenetics” emphasizes the role of OS in the regulation of genome stability, metabolic pathways, and main biological processes through epigenetic mechanisms [103]. The integration of studies on mechanisms of redox regulation and metabolic–epigenetic crosstalk can provide novel strategies for the treatment of human diseases from a new perspective [104].

Key epigenetic mechanisms, including DNA methylation, histone modifications, chromatin remodeling, non-coding RNAs (ncRNAs), and RNA methylation, dynamically regulate transcriptional programs in response to developmental cues, metabolic states, mechanical stress, and inflammatory signals. Unlike static genetic variation, epigenetic modifications are dynamic and context-dependent, enabling cells to integrate external stimuli into relatively stable regulatory states [105].

**Figure 5:**
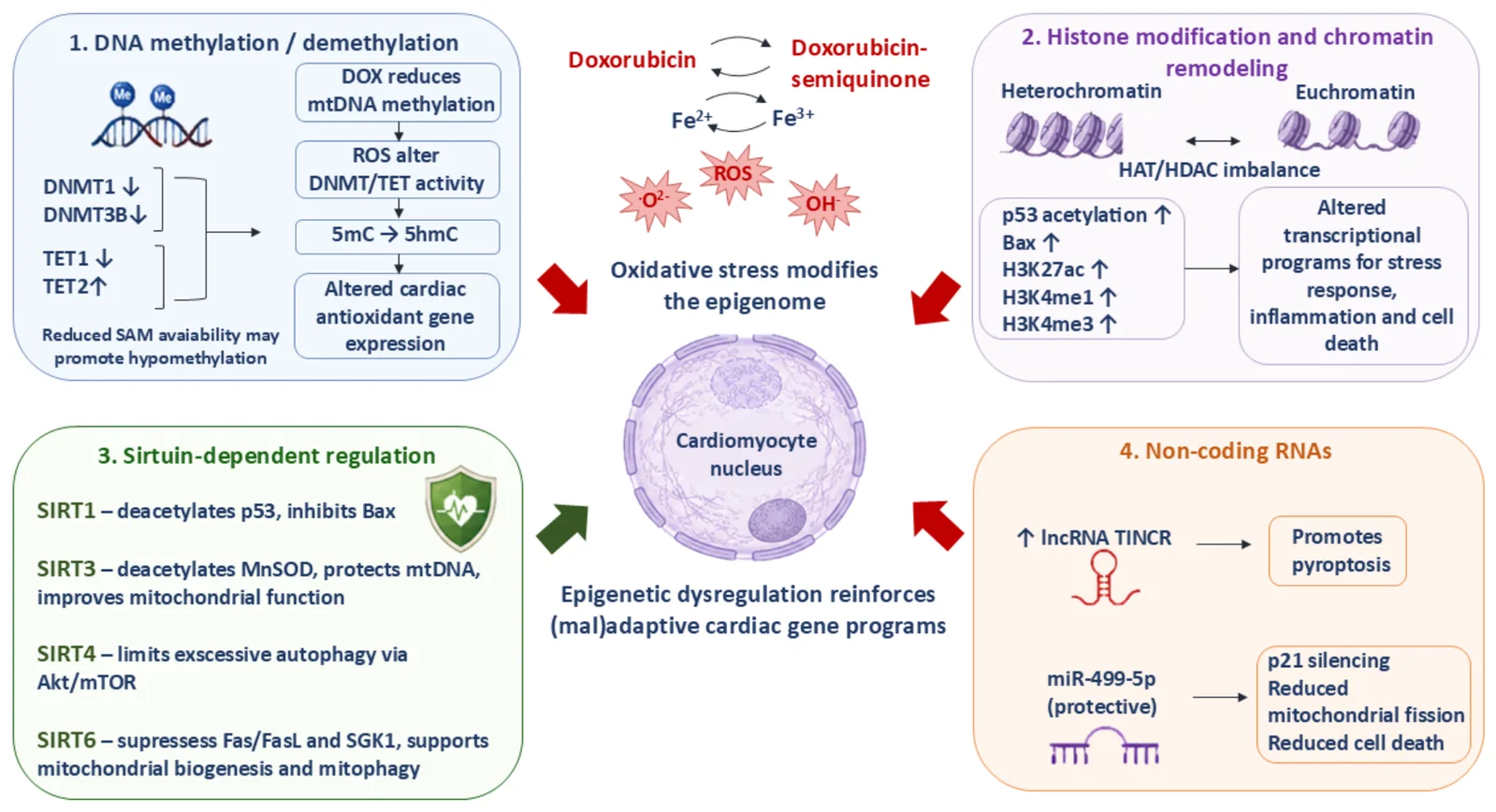
Epigenetic mechanisms in doxorubicin-induced cardiomyopathy. Schematic representation of epigenetic mechanisms involved in doxorubicin-induced cardiomyopathy. Doxorubicin redox cycling and iron-dependent ROS generation modify the cardiomyocyte epigenome and promote dysregulation of cardiac gene programs. The figure summarizes four major epigenetic levels: altered DNA methylation/demethylation, histone modifications and chromatin remodeling, sirtuin-dependent regulation, and non-coding RNA-mediated control. These mechanisms influence antioxidant defense, mitochondrial gene expression, inflammatory signaling, pyroptosis, autophagy, and cell death pathways, thereby contributing to (mal)adaptive cardiac remodeling and doxorubicin-induced cardiomyopathy. Red arrows indicate predominantly maladaptive DOX/ROS-driven epigenetic effects, whereas the green arrow highlights protective sirtuin-dependent regulation. Protective or context-dependent mechanisms within the other epigenetic categories are indicated within the corresponding panels. Abbreviations: 5hmC—5-hydroxymethylcytosine; 5mC—5-methylcytosine; Akt—protein kinase B; Bax—Bcl-2-associated X protein; DNA—deoxyribonucleic acid; DNMT—DNA methyltransferase; DOX—doxorubicin; Fas—Fas cell surface death receptor; FasL—Fas ligand; H3K4me1—monomethylation of histone H3 lysine 4; H3K4me3—trimethylation of histone H3 lysine 4; H3K27ac—acetylation of histone H3 lysine 27; HAT—histone acetyltransferase; HDAC—histone deacetylase; lncRNA—long non-coding RNA; miR-499-5p—microRNA-499-5p; MnSOD—manganese superoxide dismutase; mtDNA—mitochondrial DNA; mTOR—mechanistic target of rapamycin; p21—cyclin-dependent kinase inhibitor 1A; p53—tumor protein p53; ROS—reactive oxygen species; SAM—S-adenosylmethionine; SGK1—serum/glucocorticoid-regulated kinase 1; SIRT—sirtuin; TET—ten-eleven translocation enzyme; TINCR—terminal differentiation-induced non-coding RNA.

Anthracycline chemotherapy drug DOX-induced OS and mitochondrial dysfunction are considered major causes of cardiotoxicity, implicating mitochondria as a source and target of ROS. High levels of ROS can induce excessive mitochondrial fission, which can trigger cell apoptosis and necrosis, resulting in the deterioration of cardiac function. The balance is further disturbed by ROS-induced suppression of the mitochondrial fusion, which has been described as a defensive response to enhance resistance to stress [106]. These ROS-induced changes in mitochondrial number and morphology are described as dysregulation of mitochondrial dynamics and they represent a hallmark of DOX-induced cardiac injury, driving energy depletion, OS, and cardiomyocyte apoptosis, since DOX is promoting mitochondrial fission and suppressing mitochondrial fusion [74]. Lifestyle factors such as nutrition, drugs, and environmental exposures influence cellular pools of key cytoplasmic and mitochondrial metabolites such as S-adenosylmethionine (SAM), acetyl-CoA, and NAD^+^, which act as vital cofactors for epigenetic enzymes, ultimately leading to long-lasting, aberrant DNA methylation, histone modifications, and non-coding RNA expression [107]. The mechanisms of DOX-induced modification of the cardiomyocyte epigenome are described in detail in Figure 5 and in further paragraphs.

### 7.1. DNA Methylation in Oxidative Stress-Induced Cardiotoxicity

DNA methylation is the most extensively studied and well-understood epigenetic mechanism. As DNA methylation regulates gene expression, it is required for normal cardiac development, maturation, and health, but it is also amongst important extragenomic factors that are responsible for heart diseases. These epigenetic changes correlate with the stress-response genes that are activated in cardiac disease; however, DNA methylation is required for normal function of the heart, as shown by loss-of-function experiments [108]. DNA methylation is an attractive topic in the study of OS-induced cardiotoxicity, since the methylome may be modified by environmental factors and dietary interventions [109].

In mammalian cells, DNA methylation is the epigenetic mechanism that entails the covalent chemical addition of a single methyl group to the C5 position of the cytosine ring of DNA, resulting in 5-methylcytosine (5mC) formation. It predominantly occurs on cytosines followed by a guanine, in CpG dinucleotides, resulting in 5mCpGs. However, 5mC can also be found in non-CpG methylation contexts (CpH), where H denotes an adenine, cytosine, or thymine [110]. Unlike nuclear DNA methylation, mtDNA methylation predominantly occurs at non-CpG sites [111]. The majority of CpH methylation (mCpH) is primarily found in mCAC or mCAG formats, and it is a typical feature of mammalian neurons (extremely abundant > 2%) and embryonic stem cells (abundant 1–2%) [112]. In heart tissue, the dominant sequence context for non-CpG methylation is mCAC, and its abundance belongs to the category of detectable (<1%) [113,114]. Since both CpGs and CpHs are substrates for methylation and demethylation, the in vivo role of mCpHs is to repress transcription independently of CpG contexts. In addition, methylated CAC is a binding target of methyl-CpG binding protein 2 (MeCP2), that acts by binding to the methylated sequences of its target gene promoter and recruiting transcriptional repressors to silence gene expression [115]. MeCP2, as an important epigenetic regulator of gene transcription, is responsible for normal cardiac development and maintenance of cardiomyocyte structures [116,117].

DNA methylation has key roles in regulating transcription factor (TF) binding and gene expression. The methylation of promoter-associated CpGs suppresses gene expression, leading to gene silencing. The majority of CpG dinucleotides are methylated in the human genome (~80%), with the exception of those within CpG islands (CGIs). However, although CpG methylation is less common in CGIs, 5mCpGs are not as rare within CGIs as thought previously, most particularly in germline and embryonic stem cells [113].

CpG islands are numerous in the human genome (~30.000) and unevenly distributed throughout the genome [118]. They are localized in promoter regions, enhancers, exons, introns, repeated sequences, subtelomeric regions, and intergenic regions. CpG dinucleotides within gene promoters, enhancers, and other regulatory elements are generally infrequently methylated or completely unmethylated, and they are often found in housekeeping genes [119,120]. On the other hand, CpG dinucleotides within gene bodies, repetitive elements, and intergenic regions can be more heavily methylated [121,122].

A recently published DNA methylation atlas of normal human cell types describes the methylome of 39 normal human cell types, including heart tissue [123]. DNA methylation pattern is a key feature of cellular differentiation, modulating gene expression both in health and disease states [124,125]. In the DNA of patients with congenital heart diseases, the analysis of the location of CpG sites that were differentially methylated showed that differential methylation sites (DMSs) within CGIs of patients with congenital heart diseases were extremely likely to be more hypermethylated, in comparison with those in the control group. Further analysis of these DMSs within CGIs showed that this hypermethylation was particularly concentrated around the promoter compared with other locations [126].

For execution of the canonical form of CpG methylation, regulation of DNA methylation, and further gene silencing, both trans-acting factors and the DNA sequence are required. Trans-factors include methylation “writers” such as DNA methyl-transferases (DNMTs) that establish methylation marks and “erasers” such as ten-eleven translocation group of enzymes (TET 1–3) catalyzing demethylation of DNA, as well as “readers” such as MeCP2 and histone deacetylases (HDACs) that link methylation to the regulation of gene transcription. The covalent addition of a methyl group to the 5-carbon position of cytosine is an active process, catalyzed by DNMTs, using SAM as the methyl donor. During early embryogenesis, de novo DNMTs, such as DNMT3A and DNMT3B, are involved in initial establishment of DNA methylation patterns to unmodified DNA. These initial DNA methylation marks, introduced by DNMT3A/B, are subsequently propagated, faithfully reproduced, and stably preserved across cell divisions by the DNMT1, which is known as the canonical maintenance methyltransferase [127].

During embryonal development, heart-specific DNA methylation is required for fine-tuned spatiotemporal expression of cardiac genes involved in numerous regulatory pathways. Further dynamic changes are detected in cardiac maturation, but also in cardiac disease, as they correlate with the expression of stress-response genes that are activated in cardiac remodeling, hypertrophy, and apoptosis. DNA methylation is recognized as the most prominent epigenetic modification in disease pathogenesis and is also associated with translational potential for prevention and treatment of cardiovascular diseases. Loss-of-function experiments directed towards DNA methylation machinery led to deleterious changes in cardiac structure and function, underlining the importance of DNA methylation in proper functioning of the heart [128].

ROS can induce specific DNA hypermethylation by upregulating DNMTs; however, DNA bases can be directly modified by ROS. For example, hydroxyl radicals can lead to the formation of 5-hydroxymethylcytosine (5hmC) from 5-methylcytosine (5mC) and the resulting 5hmC has been proposed to interfere with DNMT1 to block the proper inheritance of methylation patterns [101]. In addition, ROS can reduce the availability of the cofactor SAM, thus limiting the activity of DNMTs, leading to DNA hypomethylation. Crosstalk between OS and epigenetic marks is bidirectional: OS can alter epigenetic processes by removing DNA and histone methylation marks, but also epigenetic modifications can silence nuclear genes that encode mitochondrial proteins and antioxidant systems [129].

This bidirectional, self-reinforcing loop between OS and epigenetics is an attractive topic in the study of cardiotoxicity because it may be restored by pharmaceutical and dietary interventions [109]. Each type of DNMT has a distinct epigenetic role in heart diseases, since the aberrant activity of these enzymes contributes to pathological cardiac remodeling, heart failure, atherosclerosis, and ischemic damage. DNMT1 is a key player in heart failure and cardiomyopathy, with its expression elevated in response to pathological stress. The myocardial tissue-specific *Dnmt1* knockout in rats confers a protective effect against Adriamycin-induced cardiac damage, altering gene expression and DNA methylation patterns, resulting in activation of pathways involved in myocardial protection and anti-apoptosis in response to pathological stress [130]. These results highlight the potential impact of epigenetic regulation in heart disease [131]. Treatment of murine heart cell line H9c2 with DOX induced decreased expression of the *Dnmt1* gene, which is associated with reduced mtDNA methylation level, disrupting mitochondrial gene expression and cardiac function [132].

DNMT3A/3B enzymes are the most relevant in adult cardiomyocytes, since cardiomyocytes show low rates of DNA synthesis postnatally [133]. DNMT3A is the primary cardiac isoform due to its high transcript abundance, and the heart relies on de novo demethylation enzymes to continually regulate cardiac gene expression and adapt to metabolic and/or oxidative stress. Epitranscriptomic analysis performed on primary human cardiomyocyte-like cells (HCM-ls) that were treated with 1 µM of DOX revealed that the expression of *DNMT1* and *DNMT3B* genes was downregulated in DOX-treated cells, whereas *DNMT3A* expression was not significantly modified [34].

In vitro experiments of *DNMT3A* knockout in human cardiomyocytes induced important consequences for cardiomyocyte morphology and function, such as gene expression changes of contractile proteins, aberrant activation of the glucose/lipid metabolism, and accumulation of lipid vacuoles, as well as impaired glucose metabolism and lower glycolytic enzyme expression, rendering knockout-engineered heart tissue sensitive to metabolic stress [134]. In a murine model, the cardiac-specific deletion or disruption of *DNMT3B* can lead to accelerated severe systolic insufficiency, myocardial thinning, and interstitial fibrosis [135].

DNA methylome in adult heart is generally stable, but the methylation/demethylation processes are highly dynamic during cardiomyocyte development, postnatal maturation, and disease. The heart can adapt to stimuli via the activation of specific transcriptional programs; thus, for adequate transcriptional regulation, the active process of removal of methyl groups catalyzed by TET dioxygenases (TETs) is required. The primary functions of TETs is to oxidize 5mC to 5hmC in an Fe (II)/α-ketoglutarate-dependent manner. 5hmC is not only an intermediate product of an active demethylation process but can also act as a relatively stable epigenetic mark [136].

TET1 is expressed in fetal heart, lung, and brain and in adult skeletal muscle, thymus, and ovary; however, its expression in adult heart, lung, or brain remains at undetectable levels [137]. On the other hand, TET2 and TET3 are differentially active in cardiomyocytes and cardiac fibroblasts, having critical yet opposing roles in regulating cardiac homeostasis, hypertrophy, and fibrosis [138]. TET2 represents a critical epigenetic regulator in the heart with a dual role: it protects the heart since the activation of TET2 counteracts cardiac remodeling and improves heart function; on the other hand, TET2 deletion after the onset of pathological hypertrophy averted pathogenic progression and rescued the decline in heart function [139]. TET3 expression is reduced in cardiac fibrosis, and loss of TET3 in cardiac fibroblasts leads to spontaneous DNA damage. TET3 exerts antifibrotic effects both through its demethylating activity and also through maintaining genomic integrity by facilitating repair of DNA damage [140].

Oxidative damage and subsequent disruption of the cardiomyocyte redox environment frequently results in ROS-mediated oxidation of the functional ferrous state (Fe^2+^) within the active-site into the inactive ferric state (Fe^3+^), directly inhibiting the TET enzymatic activity. In addition, ROS can oxidize the active-site cysteines and critical zinc-coordinating cysteines within the catalytic domains of TETs, leading to suppression of their catalytic function and limiting hydroxymethylation [141,142]. In addition, TET activity is suppressed under a variety of pathophysiological conditions. As previously described in detail, the accumulation of DOX metabolites in the heart enhances OS via increased production of ROS and mitochondrial disfunction [143]. RNA-sequencing data from a recent in vitro study on HCM-ls cells showed that the *TET1* gene was downregulated in DOX-treated cells, whilst *TET2* was upregulated [34].

TET demethylation activity can be inhibited by 2-hydroxyglutarate (2-HG), produced by mutated enzymes isocitrate dehydrogenases (IDHs). IDH1 and IDH2 enzymes are in-volved in citrate metabolism by converting isocitrate to α-ketoglutarate (α-KG), which is required for the biological activity of diverse dioxygenases (including TET2). Mutant IDHs, through aberrant oxidation of isocitrate, produce 2-HG, which results in TET inhibition, DNA hypermethylation, increased ROS generation, and enhanced OS sensitivity [144]. Conversely, SIRT3 (a major mitochondrial NAD^+^-dependent deacetylase) regulates ROS production by deacetylating and activating IDH2, which generates NADPH that is necessary for the regeneration of reduced glutathione, the major antioxidant responsible for preventing ROS-induced damage in response to DOX, thus protecting the heart by regulating OS, energy metabolism, and mitochondrial dynamic [145].

### 7.2. Histone Modifications and Chromatin Remodeling in Oxidative Stress Induced Cardiotoxicity

The pathogenesis of OS-induced cardiotoxicity is complex, including epigenetic modifications that disrupt physiological homeostasis, leading to aberrant gene silencing or activation and subsequent “epigenetic memory” that promotes long-term cardiomyocyte damage and heart failure [146].

Histones represent the primary structural proteins of chromatin; they assemble with DNA to form nucleosomes and they are classified as canonical core histone proteins H2A, H2B, H3, and H4, and a single linker histone H1 molecule, located outside the nucleosome. Histone modifications (HMs) are numerous; they regulate protein activity, localization, and gene expression by altering chromatin states. HMs represent the chemical alterations of histone proteins that affect the packing of DNA within a cell, since they can alter the compactness of chromatin (chromatin remodeling) by changing the affinity between histones and DNA double strands, essentially controlling whether chromatin is in an euchromatin state (loosened, accessible, open, exposed, and transcriptionally active), or in a heterochromatin state (condensed, hidden, repressed, and transcriptionally silent) [147]. Chromatin accessibility refers to the physical contact permissibility of nuclear macromolecules with DNA, where the accessible regions only comprise ~2–3% of the whole genome and more than 90% of these regions are occupied by TFs [148]. Both the transcriptional and replication activities for nucleosomal DNA segments are inherently hindered by histone proteins. HMs influence the nucleosome unwrapping and stability; they regulate transcription, DNA replication, and DNA repair [149].

The core histones at their N-terminus are rich in lysine (K) and arginine (R) residues, and they can undergo modifications called the “histone code”, which determine whether specific genes are transcriptionally active or not. The modifications can be cell- and/or tissue-specific, and they comprise post-translational modifications (PTMs) and chromatin remodeling. The specific lysine and arginine residues on these N-terminal tails act as binding sites for enzymes. The addition or removal of chemical groups (such as acetyl, methyl, or phosphate groups) alters the positive charge of the tails. In addition to most common modification processes such as methylation, acetylation, and phosphorylation, histone PTMs comprise ubiquitination, malonylation, propionylation, butyrylation, crotonylation, and lactylation, which together constitute the “histone code” [150]. Amongst PTMs, the histone methylation is more stable in comparison with other PTMs, whereas the acetylation modification is characterized by rapid turnover rates and high dynamics [151].

Three classes of histone-modifying proteins regulate the addition, removal, and interpretation of PTMs. Writer enzymes (kinases, histone methyltransferases (HMTs), and hystone acetyltransferases (HATs)) add chemical groups to histone proteins. Eraser enzymes (phosphatases, histone demethylases (HDMs), and HDACs) remove PTMs from histone proteins. Reader proteins recognize specific modifications and interpret the epigenetic signals contained within them [152].

Histone PTMs regulate pathobiological programs in the human heart by remodeling chromatin accessibility. Aberrant HMs lead to imbalanced expression of cardiovascular disease-related genes, resulting in changes in cellular phenotype and cardiac function, including inflammation, oxidative and mitochondrial stress, fibrosis and extracellular matrix remodeling, endothelial dysfunction, vascular smooth muscle cell phenotypic switching, and cardiac hypertrophy and remodeling [153].

Histone modifications due to PTMs are significantly affecting chromatin remodeling: for example, acetylation neutralizes the positive charge of lysine, which loosens chromatin and allows DNA to become accessible for gene transcription, more specifically, the acetylation of histone H3 on lysine 27 (H3K27ac) is an epigenetic marker for active transcription. In addition, H3K27ac is found both at active enhancers and promoters, rendering them accessible to TFs and other members of the transcriptional complex in order to regulate transcription of genes. Chromatin regulation changes under healthy and diseased conditions are successfully identified by histone acetylome studies, since H3K27ac level correlates with gene expression changes in human tissue of remodeled myocardium as compared to controls [154].

A group of enzymes involved in the balance of histone acetylation/deacetylation, defined as HATs and HDACs, are relevant to multiple cardiac diseases, especially in cardiac hypertrophy [155]. The importance of histone acetylation in the model of neonatal rat cardiomyocytes is emphasized by results showing that the level of H3K27ac (promotes gene transcription by regulating promoter and enhancer activity) significantly increased in cardiomyocytes following DOX treatment, resulting in upregulation of multiple cardiotoxic genes including *Bax*, *Fas*, and *Bnip3*. On the other hand, the small molecule C646, an inhibitor of HAT p300, reversed DOX-induced H3K27ac accumulation in cardiomyocytes and prevented the increase in DOX-induced DNA damage and apoptosis, further providing insights into the relationship between DOX-induced cardiotoxicity and epigenetic regulation. These findings identify H3K27ac as a molecular switch for DOX-induced activation of cardiotoxic genes, and histone acetylation as a potential target for the prevention and treatment of DOX-induced cardiotoxicity [156].

The HDAC family includes four major classes: HDAC I, IIa, IIb, and III, where HDAC III is also known as sirtuins (SIRTs), involved in cardiac protection by their function against OS and aging [157]. Amongst the epigenetic mechanisms that are influenced in response to DOX exposure, the specific changes in the transcription profiles of HDACs and HATs are reported to be deregulated in cardiac tissues, having a central position in DOX-induced cardiotoxicity [158]. Acetylation of crucial genes, such as *p53* and *Bax*, is involved in DOX-induced programmed death of cardiomyocytes. DOX increases *p53* gene expression and induces *Bax* upregulation, whereas SIRT1-mediated deacetylation of *p53* reverses the above process via inhibition of *Bax* expression. In addition, DOX induces cardiomyocyte pyroptosis by upregulating the *TINCR* gene (encodes a long non-coding RNA) by enhancing histone acetylation (H3K27ac) at the promoter region [159].

SIRTs function as NAD^+^-dependent histone deacetylases and deacylases. There are seven members in humans, with SIRT1, 6, and 7 found in the nucleus; SIRT2, which is primarily cytosolic; and SIRT3, 4, and 5, which are found in the mitochondria. Recently, particular attention has been directed towards SIRT1, 3, 4, and 6, which emerge as key regulators of endothelial integrity, mitochondrial quality control, OS, inflammatory signaling, and myocardial remodeling [160]. SIRT6 protects cardiomyocytes against DOX-induced cardiotoxicity, acting as a crucial molecular corepressor at *p53*-dependent *Fas* and *FasL* target genes, reduced cardiomyocyte apoptosis, and enhanced endogenous antioxidant defense. In addition, SIRT6 partial knockout or silencing worsened cardiac damage, remodeling, and OS injury in DOX-treated mice or cultured cardiomyocytes [161].

In DOX-induced in vitro and in vivo cardiotoxicity models, SIRT4 overexpression resulted in activation of the Akt/mTOR signaling pathway, with subsequent inhibition of excessive autophagy, which may provide a prospective therapeutic target for DOX-induced cardiotoxicity [162].

Methylation of H3 lysine 4 (H3K4) is achieved by enzymes classified as writers (methyltransferases), erasers (demethylases), and readers of methylation (effector proteins). H3K4 can be in a mono-, di-, or tri-methylated, with distinct roles in the formation of a histone code for regulation of gene transcription. One of the most important signature is the mono-methylation of histone H3 on lysine 4 (H3K4me1), which is associated with active enhancers, whereas tri-methylation of histone H3 on lysine 4 (H3K4me3) is associated with transcriptional start sites and it has a distinct role in transcriptional pause-release and elongation rather than transcriptional initiation [163].

DOX causes two out of three main types of “epigenetic memory” in heart cells of patients, involved in the pathophysiology of acute and late-onset cardiotoxicity, by inducing persistent changes in histone methylation, such as methylation and/or demethylation on H3K4 [164]. Three major types of epigenetic memory can be distinguished: cellular memory, transcriptional memory, and transgenerational memory. Anthracyclines can cause persistent, long-term changes in the cardiac epigenome at the cellular and transcriptional level, leading to DOX-induced cardiotoxicity [146].

Results obtained in a study using a DOX-induced chronic rat cardiotoxicity model showed that histone methylation modifications result in protection or aggravation of cardiotoxicity, depending on context. Sestrin2 (SESN2), a stress-inducible protein, protected against DOX-induced cardiomyopathy by regulating mitophagy and mitochondrial function. On the other hand, JMJD3 inhibited the transcription of SESN2 by reducing tri-methylation of H3K27 in the promoter region of *SESN2*. Jumonji domain-containing 3 (JMJD3) protein is a histone demethylase protein which specifically catalyzes the demethylation of H3K27 (H3K27me3) and regulates gene expression. In addition, in both the heart samples from patients with dilated cardiomyopathy and chronic DOX-stimulation induced cardiomyopathy, gene expression study revealed the overexpression of *JMJD3* and downregulation of *SESN2* [165].

In the rat cardiomyoblast cell line H9c2 used as a model system to analyze the development of cardiomyopathy in response to DOX-induced OS, it has been shown that appropriate H3K4me3 levels are critical to maintain cellular homeostasis, whereas DOX-induced epigenetic changes alter the H3K4me3 levels in the promoters of important inflammatory (*NLRP3*) and antioxidant (*Nrf2*) genes, leading to increased OS and cardiotoxicity [166].

### 7.3. Non-Coding RNAs in Oxidative Stress-Induced Cardiotoxicity

Non-coding RNAs (ncRNAs) are emerging as key regulators of gene expression, in-volved in epigenetic modifications referred to as heritable and reversible changes in the function of a gene without altering its DNA sequence. NcRNAs modulate gene expression at the transcriptional and post-transcriptional levels, influencing DNA methylation and chromatin dynamics. In addition to homeostasis maintenance, ncRNAs are involved in the pathogenesis of a wide range of diseases, such as cancer, cardiovascular disorders, neurological conditions, and metabolic diseases [167].

Advances in high-throughput sequencing techniques and bioinformatics enabled the emergence of integrative genomics approaches, combining data from various sources, such as genomics, transcriptomics, proteomics, and epigenomics, to better understand the molecular mechanisms governing cellular function and disease. The human genome is transcriptionally active. It is estimated that at least 80% of the human genome is transcribed into RNAs; however only approximately 2% of these RNAs are protein-coding. The remaining 98% of genome is transcribed into non-protein-coding RNAs, which are not translated into proteins [168,169].

Based on their length, ncRNAs are classified into two main categories: 1. long non-coding RNAs (lncRNAs, longer than 200 nucleotides) and 2. small non-coding RNAs (˂200 nucleotides) [170]. According to their function, ncRNAs are recognized as housekeeping and regulatory. The category of housekeeping ncRNAs comprises ribosomal RNAs (rRNAs), transfer RNAs (tRNAs), small nuclear RNAs (snRNAs), and small nucleolar (snoRNAs), playing essential roles in fundamental cellular processes, including transcription, splicing, and translation. Regulatory ncRNAs are primarily involved in modulating gene expression, and they are divided into circular and linear RNAs; linear RNAs are further divided into small ncRNAs (sncRNAs) and long ncRNAs (lncRNAs). The sncRNA group includes microRNAs (miRNAs), small interfering RNAs (siRNAs), and piwi-associated RNAs (piRNAs), which primarily act as negative controllers of gene expression at a post-transcriptional level but have more recently emerged as key players in RNA metabolism at the transcriptional and translational levels [171]. MiRNAs are completely complementary with their mRNA targets; thus, they can directly cleave and degrade mRNAs, affecting gene expression [172].

Recent epigenomic studies revealed that miRNAs, lncRNAs, and other ncRNA types play a critical role in epigenetic regulation during cellular differentiation and tissue development, including cardiac stem cell commitment, cardiogenic differentiation, and maturation [173]. In addition, since all epigenetic alterations are dynamic and reversible, and responsive to external inputs such as nutrition, stress, hypoxia, or xenobiotics, there is also a significant level of crosstalk between all three main epigenetic mechanisms, i.e., the non-coding RNAs, DNA methylation, and histone alterations, enabling integrated insight into development, cellular differentiation, and disease pathogenesis [174,175]. Among ncRNA, the long ncRNA and small ncRNA can affect histone modification, DNA methylation targeting, and gene silencing. MiRNAs bind directly to the DNMT1 catalytic domain with high affinity, and miR-155-5p leads to inhibition of DNMT1 enzyme activity. Exogenous miR-155-5p in cells induces aberrant DNA methylation of the genome, resulting in hypomethylation of low to moderately methylated regions [176].

This multi-level crosstalk of epigenetic components is complex and can be tissue- and/or disease-specific. For example, ncRNAs can directly or indirectly target some methyltransferases or acetyltransferases in cardiac hypertrophy and failure [177]. This role can be dual, as in the example of miR-21 that exerts opposing effects depending on cell type: in cardiomyocytes, it limits apoptosis by inhibiting pro-apoptotic pathways and mitigates the myocardial injury; however, in cardiac fibroblasts, miR-21 suppresses TGF-β1 receptor III and activates the TGF-β1 pathway, contributing to collagen deposition and heart failure progression via fibrosis [175]. Another ncRNA, miR-133, suppresses myocardial hypertrophy by downregulating DNMT3B expression and inhibiting DNA methylation [178].

Among direct targets of miRNAs are the genes that encode protein complexes and enzymes involved in the histone modification processes, such as HMTs and HDACs. The so-called lncRNA/miRNA/SIRT1 axis represents the critical post-transcriptional regulatory network in the heart [179]. On the other hand, both miRNA and lncRNAs are also regulated through histone modifications, such as methylation and HDAC overexpression [180]. NcRNAs also modify DNA methylation by regulating DNMT expression [178]. Further research on this crosstalk between ncRNAs, DNA methylation, and histone modifications might provide novel insights into the mechanisms of cardiovascular diseases [105].

Both circulating and exosomal miRNAs serve as promising tools for the early detection of cardiotoxicity, in addition to echocardiography and serum biomarkers, such as troponins or natriuretic peptides, which are detected after damage has occurred. In addition to the role of microRNAs as potential biomarkers for heart failure, DOX-induced cardiotoxicity, and coronary disease, recent studies have discovered that dysregulation of three types of ncRNAs, namely microRNAs, long non-coding RNAs, and circular RNAs (circRNAs), has been strongly linked to pathophysiological mechanisms involved in DOX-induced cardiotoxicity such as cell death, OS, and mitochondrial dysfunction [181]. Altered expressions of miRNAs are extensively studied in numerous in vitro and in vivo models of DOX-induced cardiotoxicity, as well as in clinical trials [182,183,184,185].

The circulating miRNAs miR-1 and miR-133b (muscle and/or cardio-specific miRNAs, also known as myomiRs) have emerged as potential biomarkers for early detection of cardiotoxicity in patients receiving both conventional and targeted cancer therapy and in the assessment of recovery from cardiotoxicity [186]. The abundantly expressed miRNAs in the myocardium, which play a central role in cardiogenesis, heart function, and pathology comprise miR-1, miR-133a/b, miR-208a/b, and miR-499 [187,188]. One of the cardio-specific miRNAs, miR-1, is highly expressed in cardiomyocytes and detectable in the blood, since it can be secreted into the bloodstream via extracellular vesicles such as exosomes and microvesicles. Under the conditions of stress or injury, miR-1 overexpression within cardiomyocytes is reflected by miR-1 presence in the circulation [189].

The mechanism underlying the upregulation of miR-1 in DOX-treated patients is revealed by in vitro and in vivo data, showing that miR-1 can regulate cardiomyocyte apoptosis by mediating the post-transcriptional repression of insulin-like growth factor 1 (IGF-1) [190].

In breast cancer patients who received a total dosage of 410.3 ± 9.5 mg DOX in four cycles for 3 months, cardiotoxicity occurred in 17.9% of patients, and circulating levels of miR-1, miR-133b, miR-146a, and miR-423-5p increased during the treatment, whereas miR-208a and miR-208b were undetectable. In this study, circulating miR-1 showed a superior ability than cardiac troponin I to discriminate between patients with and without cardiotoxicity induced by DOX treatment [191]. Therefore, the emergence of miRNAs as potential non-invasive biomarkers, due to their stability in biological fluids and their ability to reflect early myocardial stress and damage, can promote the personalized cardioprotective strategies, minimizing cardiovascular complications during cancer therapy and enhance patient outcomes [192].

Recent data identified cardioprotective miRNAs, such as miR-21, which post-transcriptionally regulates its target gene *BTG2* (anti-proliferative factor and B cell translocation gene 2), a member of an anti-proliferative gene family (pro-apoptotic tumor suppressor gene). Cardiac myocytes may be protected from DOX-induced injury by miR-21, through post-transcriptional regulation of *BTG2*. However, this epigenetic regulation in possible clinical trials requires a careful approach in patients, since decreasing the pro-apoptotic effect of DOX by downregulation of *BTG2* expression might reduce the therapeutic effect of DOX [193,194]. MiR-30 and miR-499-5p are two cardioprotective miRNAs downregulated in the heart after DOX treatment. To counteract the DOX-induced cardiotoxicity, the miR-30 family downregulates p53 expression, reducing mitochondrial fission and apoptosis, and also targets Beclin-1 to preserve cardiomyocyte autophagy and avoid DOX-induced apoptosis [195]. In DOX-treated cardiomyocytes, downregulated miR-30 results in DNA damage, leading to p53 activation, which in turn, suppresses miR-499-5p, resulting in increased mitochondrial fission and apoptosis. The overexpression of miR-499-5p contributed to lowered DOX cardiotoxicity by silencing p21 and inhibiting mitochondrial fission and cell death in vitro and in vivo [196].

Specific types of epigenetic regulator, long non-coding RNAs, modify gene expression through interactions with protein partners, influencing DNA repair, chromatin remodeling, and transcription. Recently discovered lncRNAs, such as choline kinase beta-divergent transcript (CHKB-DT) and non-coding RNA activated by DNA damage (NORAD), exert cardioprotective effects in murine and human models [174,197].

## 8. Nutritional and Nutraceutical Cardioprotective Interventions

**Figure 6:**
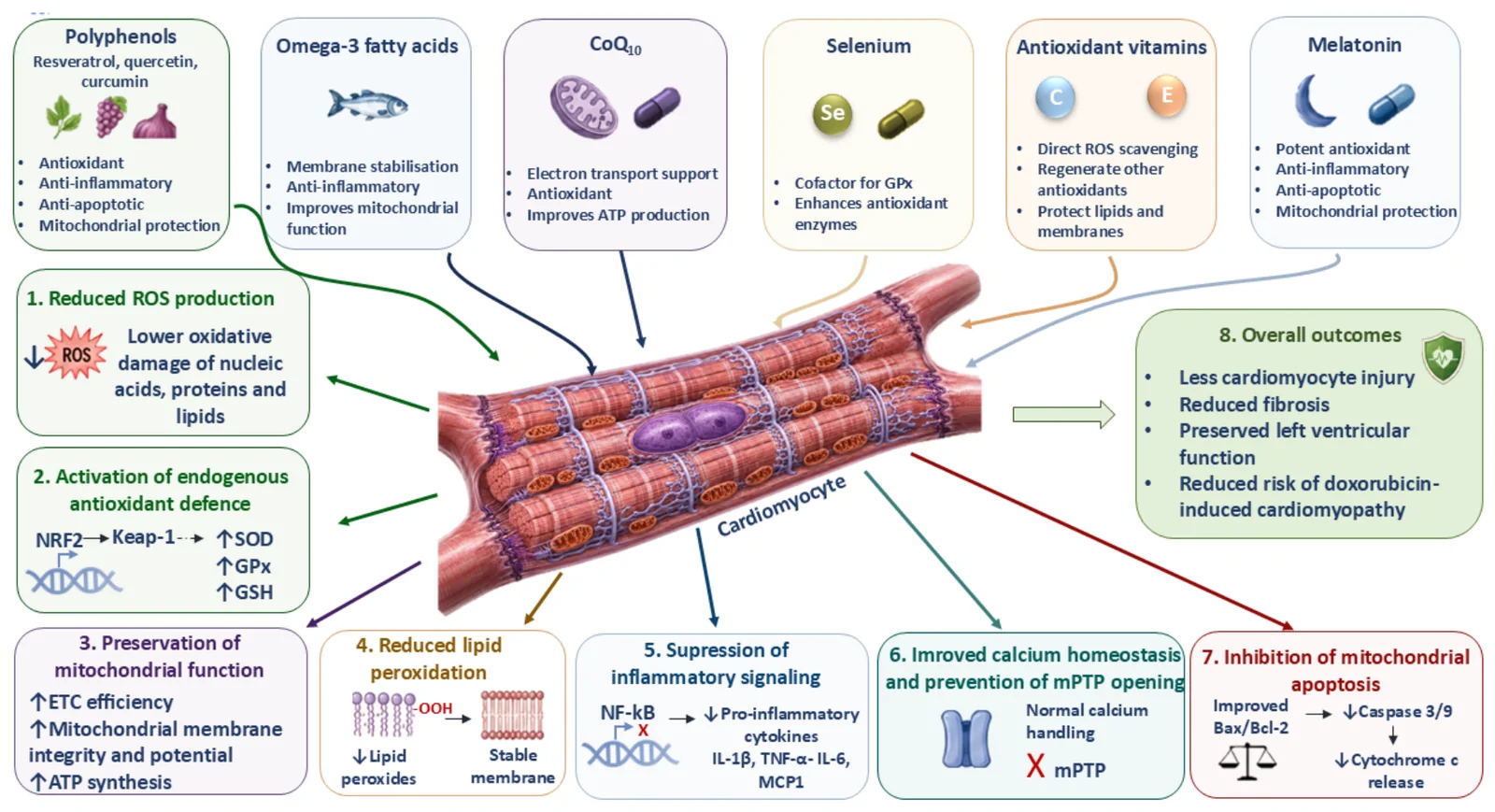
Preventive mechanisms of nutritional interventions against doxorubicin-induced cardiomyopathy. Schematic representation of the main cardioprotective mechanisms by which selected nutritional and nutraceutical interventions may attenuate DOX-induced cardiomyopathy. Polyphenols, omega-3 fatty acids, CoQ10, selenium, antioxidant vitamins, and melatonin act through complementary pathways, including reduced ROS production, activation of endogenous antioxidant defenses, preservation of mitochondrial function, suppression of lipid peroxidation and inflammatory signaling, improvement in calcium homeostasis, prevention of mPTP opening, and inhibition of mitochondrial apoptosis. Together, these mechanisms may reduce cardiomyocyte injury, limit fibrosis, preserve left ventricular function, and decrease the risk of DOX-induced cardiomyopathy. Abbreviations: ATP—adenosine triphosphate; Bax—Bcl-2-associated X protein; Bcl-2—B-cell lymphoma 2; CoQ10—coenzyme Q10; DOX—doxorubicin; ETC—electron transport chain; GPx—glutathione peroxidase; GSH—glutathione; IL-1β—interleukin-1 beta; IL-6—interleukin-6; Keap-1—Kelch-like ECH-associated protein 1; MCP-1—monocyte chemoattractant protein-1; mPTP—mitochondrial permeability transition pore; NF-κB—nuclear factor kappa B; Nrf2—nuclear factor erythroid 2-related factor 2; ROS—reactive oxygen species; Se—selenium; SOD—superoxide dismutase; TNF-α—tumor necrosis factor alpha.

Given the multifactorial pathophysiology of cancer therapy-related cardiotoxicity, nutritional and nutraceutical strategies have attracted increasing interest as potentially multitargeted approaches capable of attenuating OS, preserving mitochondrial function, modulating inflammation, and limiting regulated cardiomyocyte death. Dietary bioactive compounds may exert pleiotropic effects through the modulation of redox-sensitive transcriptional pathways, mitochondrial quality-control mechanisms, inflammatory signaling, lipid peroxidation, and multiple forms of regulated cell death (Figure 6) [22,23]. This concept of nutritional cardioprotection may be particularly relevant in long-term cancer survivorship, in which early attenuation of oxidative and inflammatory injury could potentially reduce the cumulative burden of subclinical myocardial damage. However, translation into routine cardio-oncology practice remains limited, as the available evidence is derived predominantly from in vitro and animal studies, whereas adequately powered randomized clinical trials remain scarce. Nutritional and nutraceutical approaches should therefore be regarded as promising adjuncts, rather than established alternatives, to guideline-directed cardiovascular surveillance and evidence-based pharmacological cardioprotection [14,22]. It should be emphasized that the evidence base for nutritional cardioprotection is substantially more extensive for AIC than for radiation-induced heart disease; consequently, the latter is represented predominantly by preclinical studies and limited exploratory clinical evidence.

### 8.1. Polyphenols

Polyphenols are a heterogeneous class of plant-derived bioactive compounds characterized by one or more phenolic rings and multiple hydroxyl groups. They include stilbenes, flavonols, flavones, flavanols, anthocyanins, catechins, phenolic acids, lignans, and several related plant secondary metabolites. Although traditionally regarded as direct antioxidants, polyphenols are now recognized as modulators of intracellular signaling pathways involved in OS adaptation, mitochondrial function, inflammatory regulation, autophagy, ferroptosis, apoptosis, and epigenetic remodeling [4,23,198,199].

#### 8.1.1. Resveratrol

Resveratrol, a stilbene polyphenol abundant in grapes, berries, and red wine, has been extensively investigated in experimental models of cardiometabolic disease and AIC. The major cardioprotective mechanisms of resveratrol include activation of sirtuin-1 (SIRT1), stimulation of AMPK signaling, enhancement of Peroxisome proliferator-activated receptor gamma coactivator 1-alpha (PGC-1α)-mediated mitochondrial biogenesis, suppression of NF-κB-driven inflammation, and activation of Nrf2-dependent antioxidant responses [200,201]. In experimental models, resveratrol attenuates DOX-induced cardiomyocyte injury by reducing ROS generation, preserving mitochondrial membrane potential, improving mitochondrial bioenergetics, and inhibiting apoptosis. In the context of DOX-induced cardiotoxicity, resveratrol has been shown to attenuate myocardial injury through mitochondrial stabilization and activation of SIRT1-dependent cardioprotective signaling. SIRT1 activation is mechanistically relevant because this NAD^+^-dependent deacetylase regulates mitochondrial homeostasis; OS responses; and cellular survival pathways, including PGC-1α-dependent mitochondrial biogenesis and p53-dependent apoptotic signaling [200,202,203].

Resveratrol also activates the Nrf2/Keap1 pathway, a master regulator of antioxidant and cytoprotective gene expression. Under basal conditions, Nrf2 is sequestered by Keap1 and targeted for ubiquitin-proteasomal degradation. OS or electrophilic compounds modify redox-sensitive cysteine residues on Keap1, allowing Nrf2 stabilization; nuclear translocation; and transcriptional activation of antioxidant response element (ARE)-dependent genes, including those encoding SOD, catalase, GPx, heme oxygenase-1, NAD(P)H quinone oxidoreductase 1, and other phase II detoxification enzymes [204,205].

More recent studies have expanded the mechanistic framework by implicating ferroptosis. Resveratrol has been shown to attenuate DOX-induced cardiotoxicity by modulating MAPK signaling and reducing ferroptotic myocardial injury [206]. In addition, activation of the p62-Nrf2 axis appears to protect against DOX-induced ferroptosis, suggesting that resveratrol-mediated Nrf2 activation may contribute not only to antioxidant defense but also to preservation of iron and lipid peroxide homeostasis [207].

In addition to SIRT1- and Nrf2-mediated antioxidant signaling, resveratrol may modulate the balance between autophagy and apoptosis in DOX-induced cardiotoxicity. Gu et al. [208] expanded the mechanistic framework of resveratrol-mediated cardioprotection beyond its classical antioxidant activity. The authors demonstrated that DOX disrupts the balance between autophagy and apoptosis through E2F1/mTORC1 and E2F1/AMPKα2 signaling, leading to autophagy inhibition and apoptosis promotion. Resveratrol attenuated DOX-induced cardiotoxicity by restoring autophagic activity and reducing apoptotic cardiomyocyte death. This study is particularly relevant because it indicates that resveratrol should not be regarded merely as a direct free radical scavenger, but rather as a modulator of adaptive cellular stress responses, including autophagy, apoptosis, and potentially mitochondrial quality control. Zhang et al. [209] sought to overcome two major pharmacokinetic limitations of resveratrol, poor aqueous solubility and low oral bioavailability, by developing resveratrol-loaded solid lipid nanoparticles (Res-SLN). Nanoencapsulation enhanced resveratrol stability, controlled its release, and improved its pharmacokinetic profile. In an experimental model of DOX-induced cardiotoxicity, treatment with Res-SLN preserved heart rate, LVEF, and fractional shortening, while reducing ultrastructural myocardial damage. These findings indicate that lipid-based nanoformulations may improve the delivery and biological efficacy of resveratrol; however, their translational potential in cardio-oncology requires confirmation in well-designed clinical studies. A comparative in vitro study evaluated resveratrol alongside established cardioprotective agents, including dexrazoxane and carvedilol, in differentiated H9c2 cells exposed to DOX. Following 24 h pretreatment, resveratrol produced a greater improvement in cell viability than the comparator interventions. Both resveratrol and dexrazoxane attenuated ROS generation after 3 h of DOX exposure, whereas all tested treatments reduced ROS levels after 24 h. Although these findings are limited by the use of an in vitro screening model and cannot be directly extrapolated to clinical practice, they support further investigation of resveratrol in more physiologically relevant preclinical models and clinical studies [210]. The timing of resveratrol administration may substantially influence its cardioprotective efficacy. In a rat model of DOX-induced cardiotoxicity, both prophylactic and post-exposure resveratrol treatment attenuated echocardiographic evidence of cardiac dysfunction and reduced circulating markers of myocardial injury, including lactate dehydrogenase (LDH) and creatine kinase-MB (CK-MB). However, prophylactic administration provided greater protection against myocardial apoptosis and fibrosis than treatment initiated after DOX exposure. These findings suggest that resveratrol-based cardioprotection may be more effective when initiated before or concomitantly with cardiotoxic therapy, rather than after structural myocardial injury has become established [211].

Collectively, preclinical evidence identifies resveratrol as a multitarget polyphenol with potential cardioprotective effects in DOX-induced cardiotoxicity. Its actions extend beyond direct free-radical scavenging and include attenuation of ROS generation, preservation of cardiac function, inhibition of apoptosis and fibrosis, modulation of autophagy–apoptosis homeostasis, and improvement in mitochondrial stress responses. Despite these promising experimental findings, the clinical translation of resveratrol into cardio-oncology practice remains limited, and adequately powered randomized clinical trials specifically evaluating its efficacy in preventing AIC are currently lacking. Major translational challenges include low oral bioavailability; rapid systemic metabolism; extensive glucuronidation and sulfation; variability among formulations; and uncertainty regarding the optimal dose, treatment duration, and timing of administration relative to chemotherapy.

#### 8.1.2. Quercetin and Flavonoids

Quercetin, a flavonol abundant in onions, apples, and berries, exhibits potent antioxidant and anti-inflammatory properties. Experimental studies demonstrate that quercetin can attenuate anthracycline-induced cardiac injury. In vitro and in vivo models indicate that quercetin reduces OS, restores antioxidant enzyme activity, preserves mitochondrial membrane integrity, and suppresses cardiomyocyte apoptosis. Quercetin scavenges ROS directly and chelates transition metals, preventing Fenton chemistry [198]. Additionally, quercetin modulates inflammatory signaling by inhibiting NF-κB activation and reducing pro-inflammatory cytokine production (TNF-α, IL-1β, and IL-6) [212,213].

Early mechanistic studies demonstrated that quercetin attenuated DOX-induced cardiotoxicity in both in vitro and in vivo models by reducing OS and increasing the expression of Bmi-1, a polycomb group protein involved in the transcriptional regulation of mitochondrial function and cellular redox homeostasis. Bmi-1 upregulation was associated with reduced DOX-induced DNA double-strand breaks and preservation of DNA repair capacity in cardiomyocytes. These findings indicate that quercetin-mediated cardioprotection extends beyond direct ROS scavenging and may involve the modulation of stress-responsive transcriptional networks, mitochondrial homeostasis, and genomic integrity [214].

More recent in vivo evidence indicates that oral quercetin administration partially preserved cardiac function in animals receiving chronic DOX treatment, restored NT-proBNP concentrations toward control values, attenuated increases in circulating markers of myocardial injury, and reduced genomic damage, as reflected by decreased γH2AX and 8-hydroxy-2′-deoxyguanosine levels. These effects were accompanied by modulation of Nrf2- and SOD1-associated antioxidant responses [215]. Quercetin’s ability to chelate iron and modulate oxidative metabolism is particularly relevant in AIC, where iron-mediated lipid peroxidation and ferroptosis contribute to myocardial injury. Flavonoids, including quercetin and related derivatives, have been investigated for their combined iron-chelating, antioxidant, and carbonyl reductase-inhibitory effects, which may collectively mitigate AIC [216]. Collectively, these findings support the potential of quercetin to limit OS, DNA damage, and early biochemical and functional manifestations of AIC.

Combination strategies have also been investigated. In a rat model of DOX-induced cardiomyopathy, quercetin administered in combination with candesartan attenuated increases in biochemical markers of tissue injury, including creatine kinase (CK) and LDH, and favorably influenced the serum lipid profile. These findings suggest that quercetin may enhance the cardioprotective effects of pharmacological interventions targeting neurohormonal activation and adverse cardiovascular remodeling [217]. Although limited to a preclinical model, this study supports the potential use of nutraceutical compounds as adjuncts to established cardioprotective therapies rather than as stand-alone alternatives.

Several studies have investigated nanodelivery strategies to overcome the poor aqueous solubility, limited systemic bioavailability, and inadequate cardiac accumulation of quercetin. A carboxyl-functionalized MCM-41 mesoporous silica nanosystem co-encapsulating DOX and quercetin provided sustained drug release and attenuated DOX-induced cytotoxicity in H9c2 cardiomyoblasts [218]. Similarly, the combined administration of liposomal DOX and free quercetin reduced cardiotoxicity in both in vitro and in vivo models, suggesting that quercetin may complement anthracycline delivery systems designed to improve the therapeutic index of anthracyclines [219]. More recently, cardiac-targeted, quercetin-loaded poly(lactic-co-glycolic acid) nanoparticles functionalized with a targeting peptide were evaluated in a mouse model of DOX-induced cardiotoxicity. This formulation restored endogenous antioxidant defenses; preserved mitochondrial membrane potential; reduced ROS generation, lipid peroxidation, protein carbonylation, and circulating troponin I and CK-MB levels; and enhanced myocardial Nrf2/HO-1 signaling [220]. Collectively, these preclinical findings suggest that nanoencapsulation and cardiac-targeting approaches may improve quercetin delivery and cardioprotective efficacy; however, their clinical translation will require comprehensive evaluation of pharmacokinetics, biodistribution, long-term safety, antitumor efficacy, and manufacturing reproducibility.

Emerging mechanistic evidence suggests that quercetin may modulate regulated cell-death pathways and mitochondrial quality-control mechanisms. In an experimental model of DOX-associated atrial fibrillation, quercetin restored autophagic flux through the EGFR/TFEB/ATG5-regulated autophagy–lysosomal pathway and attenuated atrial electrical and structural abnormalities [221]. These findings extend the potential cardioprotective effects of quercetin beyond the preservation of ventricular function and indicate that modulation of autophagy may contribute to its antiarrhythmic activity, independently of its direct antioxidant properties. In a separate study, quercetin attenuated DOX-induced cardiotoxicity through modulation of the HO-1/PGC-1α–ALOX5 signaling axis, thereby linking arachidonic acid metabolism and OS with mitochondrial dysfunction, iron-dependent lipid peroxidation, and ferroptotic cardiomyocyte injury [222]. This mechanism is consistent with growing evidence that ferroptosis contributes to AIC by integrating disturbances in iron homeostasis, phospholipid peroxidation, mitochondrial injury, and regulated cardiomyocyte death.

The potential impact of quercetin co-administration on the antitumor efficacy of DOX is an important translational consideration. In an in vitro study, quercetin enhanced DOX cytotoxicity in human breast cancer cells while attenuating its adverse effects in non-malignant mammary epithelial cells and AC16 human cardiomyocyte-like cells. Mechanistically, quercetin increased intracellular DOX accumulation in breast cancer cells by downregulating ATP-binding cassette (ABC) transporters involved in drug efflux, including P-glycoprotein, breast cancer resistance protein (BCRP), and multidrug resistance-associated protein-1 (MRP-1) [223]. Although these findings require confirmation in appropriate in vivo tumor models and clinical studies, they raise the possibility that quercetin could improve the therapeutic index of DOX by enhancing tumor-cell sensitivity while limiting injury to non-malignant tissues. This consideration is essential because any antioxidant or nutraceutical strategy intended for cardio-oncology should be evaluated not only for its cardioprotective efficacy but also for its effects on tumor response, chemotherapy pharmacokinetics, and long-term oncological outcomes.

Finally, plant-derived extracts containing quercetin and related polyphenols may also contribute to cardioprotection, although their interpretation is more complex because they represent multicomponent mixtures rather than isolated compounds. In a rat model of DOX-induced cardiotoxicity, an aqueous–methanolic extract of *Jatropha mollissima* leaves, reported to contain several phenolic constituents, including quercetin and rutin, attenuated biochemical and histopathological indices of cardiac injury and modulated OS and inflammatory responses [224]. These findings support further investigation of polyphenol-rich phytochemical preparations; however, the observed effects cannot be attributed to an individual constituent, and their translational relevance remains limited by variability in phytochemical composition, insufficient extract standardization, uncertain pharmacokinetics and bioavailability, potential herb–drug interactions, and incomplete safety characterization.

Despite promising experimental findings, the available evidence supporting quercetin in cancer therapy-related cardiotoxicity remains predominantly preclinical. Currently, adequately powered randomized clinical trials are insufficient to support its routine use for the prevention of DOX-induced cardiotoxicity. Future investigations should prioritize standardized and well-characterized formulations; optimization of dosing and treatment timing; comprehensive pharmacokinetic and biodistribution analyses; biomarker-guided patient stratification; and concurrent assessment of cardiovascular safety, antitumor efficacy, and long-term oncological outcomes.

#### 8.1.3. Curcumin

Curcumin, the principal curcuminoid of turmeric (*Curcuma longa*), has been extensively investigated as a pleiotropic cardioprotective polyphenol in experimental models of DOX-induced cardiotoxicity. Its protective effects are mediated through antioxidant, anti-inflammatory, anti-apoptotic, anti-pyroptotic, mitochondria-preserving, and potentially anti-fibrotic mechanisms. Overall, the available literature supports curcumin as a multi-target modulator of the major molecular pathways implicated in AIC, although most evidence remains preclinical.

Several experimental studies have demonstrated that curcumin attenuates DOX-induced myocardial injury through complementary antioxidant, anti-inflammatory, and anti-apoptotic mechanisms. Its cardioprotective effects include activation of Nrf2-dependent signaling, enhancement of endogenous antioxidant enzyme activity, direct scavenging of ROS, and attenuation of oxidative DNA and protein damage [225]. Benzer et al. showed that curcumin reduced DOX-induced cardiotoxicity through antioxidant, anti-inflammatory, and anti-apoptotic effects, supporting its broad cytoprotective profile in cardiac tissue [226]. Its protective activity has also been observed in the liver, suggesting broader systemic effects mediated, at least in part, by modulation of inducible nitric oxide synthase, NF-κB, and TNF-α signaling and attenuation of inflammatory and nitrosative stress responses associated with anthracycline exposure [227]. Additionally, curcumin has been shown to modulate apoptosis-related proteins, including BAX, BCL-2, and caspase-3, reducing inflammatory cytokine production and preventing inflammatory-mediated cardiac injury [227,228].

Recent studies have expanded the mechanistic framework of curcumin-mediated cardioprotection beyond classical antioxidant activity. Curcumin has been shown to modulate the Rac1/TWEAK/Fn14/NF-κB signaling axis in experimentally induced DOX cardiotoxicity, thereby attenuating pro-inflammatory receptor signaling and downstream NF-κB activation [229]. Additional findings suggest that curcumin may also improve DOX-induced cardiac dysfunction by regulating apelin expression, implicating the apelinergic system as a potential mediator of its cardioprotective and cardiometabolic effects [230].

Curcumin may also modulate regulated cell-death pathways implicated in AIC. Experimental evidence indicates that curcumin attenuates DOX-induced cardiomyocyte injury through mTOR-dependent regulation of autophagy and pyroptosis [231]. These effects have been associated with suppression of the NLRP3 inflammasome/caspase-1/gasdermin D signaling axis, suggesting that inhibition of inflammatory cell death may contribute to curcumin-mediated cardioprotection under conditions of DOX-induced OS. This mechanistic concept is consistent with recent reviews identifying curcumin and other natural bioactive compounds as potential modulators of NLRP3 inflammasome activation in chemotherapy-related cardiotoxicity [232,233].

Mitochondrial preservation appears to represent an important component of curcumin-mediated cardioprotection. Experimental evidence indicates that curcumin attenuates DOX-induced OS and mitochondrial dysfunction through a 14-3-3γ-dependent mechanism that promotes the mitochondrial translocation of Bcl-2 and preserves mitochondrial integrity [234]. In vivo studies have further demonstrated that curcumin reduced OS, inflammation, and apoptosis in DOX-treated rats, with ultrastructural and computational evidence supporting preservation of cardiac tissue architecture [225]. In BALB/c mice, curcumin administered in combination with a reduced dose of DOX decreased lipid peroxidation, protein carbonylation, and histopathological cardiac injury and was associated with improved survival [235]. These findings suggest that curcumin may enhance the cardiac tolerability of anthracycline treatment in selected experimental settings; however, any potential improvement in the therapeutic index requires confirmation through concurrent assessment of antitumor efficacy and cardiotoxicity.

A major translational limitation of curcumin is its poor aqueous solubility, limited intestinal absorption, rapid metabolism, and consequently low systemic bioavailability. To overcome these pharmacokinetic constraints, several studies have investigated nanoformulations and combined drug-delivery systems. Curcumin nanoparticles have been shown to attenuate DOX-induced cardiotoxicity in vivo, suggesting that nanoencapsulation may enhance curcumin delivery and biological activity [236]. Similarly, curcumin-coated gold nanoparticles reduced DOX-induced cardiac injury in mice, at least partly through modulation of apoptotic signaling [237]. Silicified curcumin microspheres have also been reported to improve curcumin bioavailability and exert cardiovascular protective effects predominantly through activation of the Nrf2/HO-1 pathway [238]. Additional preclinical studies have evaluated curcumin as part of combined anthracycline-delivery platforms. A complex micellar system co-encapsulating curcumin and DOX attenuated cardiotoxicity while preserving antitumor efficacy [239]. Tumor-targeted approaches have included pH-responsive sodium alginate-derived prodrug nanoparticles designed to promote the preferential release of DOX and curcumin within the tumor microenvironment, thereby enhancing tumor-selective drug delivery and potentially reducing systemic toxicity [240]. Another nanoparticle-based study reported one-step formation of lipid-polyacrylic acid-calcium carbonate nanoparticles for co-delivery of DOX and curcumin, further reinforcing the translational relevance of co-delivery platforms [241]. Collectively, these strategies may address both the limited bioavailability of curcumin and the need to maintain antineoplastic efficacy while reducing off-target cardiac injury. However, their clinical translation will require rigorous evaluation of pharmacokinetics, biodistribution, nanoparticle-associated toxicity, manufacturing reproducibility, and long-term cardiovascular and oncological outcomes.

The gut–heart axis may represent an additional mechanism contributing to curcumin-mediated cardioprotection. In a rat model of DOX-induced cardiotoxicity, Zn(II)–curcumin supplementation attenuated intestinal dysbiosis and disturbances in zinc homeostasis [242]. These findings extend the potential cardioprotective actions of curcumin beyond direct effects on myocardial OS and suggest that modulation of the gut microbiota, trace-element homeostasis, and systemic inflammatory and metabolic signaling may influence the development of anthracycline-induced cardiac injury.

From a translational perspective, the timing of curcumin administration may substantially influence its cardioprotective efficacy. In an experimental model of DOX-induced cardiomyopathy, prophylactic administration provided greater protection against cardiac injury than treatment initiated after DOX exposure [243]. These findings suggest that curcumin-based cardioprotection may be more effective when initiated before or concomitantly with cardiotoxic therapy, rather than after structural myocardial damage has become established. Collectively, extensive preclinical evidence identifies curcumin as a mechanistically promising, multitarget cardioprotective polyphenol in DOX-induced cardiotoxicity. However, direct clinical evidence remains insufficient to support its routine use in cardio-oncology. Future studies should prioritize standardized and well-characterized formulations; optimization of dose and treatment timing; comprehensive pharmacokinetic and bioavailability assessments; biomarker-guided patient stratification; and evaluation of sensitive cardiac endpoints, including myocardial strain and circulating biomarkers. Importantly, cardiovascular outcomes should be assessed concurrently with antitumor efficacy, treatment-related toxicity, and long-term oncological outcomes.

Among the polyphenols investigated for anthracycline cardioprotection, curcumin is supported by one of the few available clinical studies directly evaluating a nutraceutical intervention in patients receiving DOX. In a small randomized, double-blind, placebo-controlled trial involving patients with breast cancer, six months of nanocurcumin supplementation was associated with preservation of selected echocardiographic parameters and a lower occurrence of marked LVEF decline [244]. However, between-group differences in several cardiac parameters did not reach statistical significance, and no cases of symptomatic cardiomyopathy were observed during the study period. These findings therefore provide a preliminary clinical signal rather than definitive evidence of cardioprotective efficacy. The use of a nanoformulation is nevertheless pharmacologically relevant, as it is intended to overcome the poor aqueous solubility, limited gastrointestinal absorption, rapid metabolism, and low systemic bioavailability of native curcumin. Larger, adequately powered trials incorporating myocardial strain, cardiac biomarkers, clinically defined cardiotoxicity, and long-term oncological outcomes are required to establish its potential role in cardio-oncology.

Nevertheless, the clinical evidence remains preliminary. Larger, adequately powered multicenter randomized controlled trials are needed to confirm cardioprotective efficacy; determine the optimal dose, formulation, timing, and duration of treatment; and evaluate sensitive cardiovascular endpoints, including GLS, cardiac troponins, natriuretic peptides, clinically defined cardiotoxicity, and long-term cardiovascular outcomes. Such studies should also establish whether curcumin alters the pharmacokinetics or antineoplastic efficacy of concomitant chemotherapy. This consideration is particularly important because earlier experimental studies suggested that dietary curcumin may attenuate chemotherapy-induced apoptosis in certain tumor models [245]. Future trials should therefore incorporate concurrent assessment of cardiovascular protection, tumor response, cancer-related outcomes, and potential nutraceutical–drug interactions.

Polyphenols have also been investigated in the context of radiation-induced cardiac injury, although the evidence is substantially more limited than that available for AIC. In a rat model of radiation-induced heart injury, curcumin attenuated the radiation-associated increase in IL-4 and IL-13 signaling; reduced the expression of IL4Rα1, IL13Rα2, and Dual oxidase 1 and 2; and decreased myocardial infiltration by inflammatory cells, supporting combined anti-inflammatory and redox-modulating effects [246]. Similarly, zingerone, a naturally occurring phenolic compound derived from ginger, reduced γ-radiation-induced cardiac biochemical and histological injury in rats, accompanied by lower lipid peroxidation, restoration of glutathione and catalase activity, suppression of TNF-α, myeloperoxidase and COX-2-associated inflammatory responses, reduced caspase-3 expression and DNA fragmentation, and preservation of mitochondrial respiratory-complex activity [247]. These findings suggest that the antioxidant, anti-inflammatory, mitochondrial, and anti-apoptotic actions attributed to polyphenols in DOX-induced cardiotoxicity may also be relevant to radiation-induced myocardial injury. However, these observations remain predominantly preclinical and require validation in models that more closely reproduce contemporary fractionated thoracic radiotherapy and, ultimately, in prospective clinical studies.

### 8.2. Omega-3 Polyunsaturated Fatty Acids

Omega-3 polyunsaturated fatty acids (omega-3 PUFAs), particularly eicosapentaenoic acid (EPA) and docosahexaenoic acid (DHA), represent one of the most clinically relevant classes of nutritional compounds investigated for attenuation of chemotherapy-associated toxicities. Their biological effects extend beyond classical cardiometabolic protection and include modulation of membrane lipid composition, suppression of inflammatory signaling, generation of specialized pro-resolving lipid mediators, preservation of mitochondrial function, and regulation of OS responses. In the context of cardio-oncology, omega-3 PUFAs are of particular interest because AIC is characterized by mitochondrial dysfunction, excessive ROS generation, lipid peroxidation, inflammatory activation, calcium dysregulation, and progressive cardiomyocyte injury [248,249]. Importantly, compared with many other nutraceuticals, omega-3 fatty acids have direct clinical evidence in pediatric oncology, although the overall clinical evidence base remains limited.

Preclinical data support these clinical observations. Mechanistically, EPA and DHA may reduce DOX-induced myocardial injury through several complementary pathways. Incorporation of omega-3 PUFAs into cardiomyocyte membranes alters membrane fluidity, lipid raft organization, receptor signaling, and susceptibility to inflammatory eicosanoid production. By partially replacing arachidonic acid within membrane phospholipids, omega-3 PUFAs reduce the generation of pro-inflammatory lipid mediators and promote the biosynthesis of resolvins, protectins, and maresins, which facilitate resolution of inflammation. Omega-3 PUFAs may also activate antioxidant and cytoprotective signaling pathways, including the Nrf2/HO-1 axis, and reduce ROS production by improving mitochondrial function and limiting oxidative membrane injury [248]. In H9c2 rat cardiomyoblasts, EPA and DHA attenuated DOX-induced ROS generation and preserved mitochondrial membrane potential, at least partly by maintaining uncoupling protein 2 expression [250]. These findings suggest that omega-3 fatty acids may protect against DOX-induced cellular injury through modulation of mitochondrial redox homeostasis, rather than solely through direct free-radical scavenging. Additional evidence indicates that DHA attenuates DOX-induced cardiotoxicity by suppressing the NF-κB/iNOS-derived nitric oxide signaling axis, thereby limiting inflammatory activation and excessive RNS generation [251]. Collectively, these findings implicate the interplay between mitochondrial dysfunction, inflammation, and nitrosative stress as a potential target of omega-3 fatty acid-mediated cardioprotection.

Uygur et al. [249] showed that fish-derived omega-3 fatty acids attenuated acute DOX-induced cardiotoxicity in rats. Omega-3 pretreatment reduced myocardial MDA levels, increased SOD and GPx activities, reduced TUNEL-positive apoptotic cardiomyocytes, and improved histopathological myocardial injury. These findings support a mechanistic role for omega-3 fatty acids in suppressing lipid peroxidation, reinforcing endogenous antioxidant defenses, and limiting apoptosis in anthracycline-exposed myocardium. More recently, Monte et al. [252] investigated omega-3 supplementation in a chronic DOX cardiotoxicity model and reported attenuation of structural and functional cardiac alterations, accompanied by reduced protein carbonylation. Interestingly, this protection was not directly related to the ceramide pathway, suggesting that omega-3-mediated cardioprotection may be more closely associated with oxidative protein damage, membrane remodeling, and mitochondrial-inflammatory signaling than with ceramide-dependent mechanisms alone.

Recent experimental evidence has broadened the potential protective effects of omega-3 fatty acids beyond direct myocardial injury. In preclinical models of DOX toxicity, omega-3 supplementation attenuated cachexia and reduced genotoxic damage, as reflected by decreased micronucleus formation [253]. Although these findings were not primarily based on cardiovascular endpoints, they may be relevant to cardio-oncology because systemic inflammation, metabolic deterioration, and treatment-related cachexia can further compromise cardiovascular resilience during chemotherapy. Omega-3 fatty acids have also been shown to attenuate DOX-induced hepatic and renal injury by reducing OS and preserving endogenous antioxidant defenses. In the liver, these effects were additionally associated with suppression of NF-κB/p65-mediated inflammatory signaling [254,255]. Collectively, these findings suggest that omega-3 supplementation may mitigate the broader systemic oxidative and inflammatory toxicity associated with anthracycline exposure, although the extent to which these extracardiac effects contribute to direct cardioprotection remains to be established.

A particularly interesting extension of this field involves specialized pro-resolving lipid mediators derived from omega-3 fatty acids. Resolvin E1, an EPA-derived mediator, was shown to protect against DOX-induced cardiotoxicity by inhibiting OS, autophagy dysregulation, and apoptosis through AKT/mTOR-related signaling [256]. In another study, resolvin E1 attenuated DOX-induced cardiac fibroblast senescence, with IL-1β identified as an important mediator [257]. These findings are highly relevant to long-term cardiotoxicity because fibroblast senescence, sterile inflammation, and impaired resolution of inflammation may contribute to extracellular matrix remodeling, fibrosis, and progressive ventricular dysfunction after anthracycline exposure. Thus, the benefit of omega-3 fatty acids may not be limited to prevention of acute oxidative injury but may also involve active resolution of inflammation and limitation of fibro-inflammatory remodeling.

The strongest clinical evidence in the specific context of DOX-induced cardiotoxicity comes from a randomized controlled trial conducted in children with newly diagnosed ALL. El Amrousy et al. [258] randomized 60 children to receive either omega-3 fatty acids at a dose of 1000 mg/day for six months in addition to their usual DOX-containing chemotherapy protocol, or chemotherapy alone. After six months, omega-3 supplementation was associated with significantly lower MDA levels and significantly higher glutathione and SOD activity compared with controls, indicating an improvement in systemic redox balance. In parallel, troponin I, CK-MB, and NT-proBNP increased significantly in the control group but remained unchanged in the omega-3 group. Echocardiographic assessment also showed preservation of systolic function, including peak mitral annular systolic velocity and two-dimensional GLS, in omega-3-treated children, whereas the control group developed significant impairment of left ventricular function. This trial is particularly important because it directly links omega-3 supplementation with reduced OS, preservation of cardiac biomarkers, and attenuation of early subclinical DOX cardiotoxicity in a pediatric oncology population.

Additional pediatric oncology data support the broader cardiometabolic relevance of omega-3 supplementation during ALL treatment. In a secondary analysis of a randomized controlled trial, Barbosa-Cortés et al. [259] evaluated long-chain omega-3 PUFA supplementation in children undergoing treatment for ALL and reported favorable effects on selected cardiometabolic factors. Although this study was not designed primarily to assess AIC, it is relevant because cardiometabolic abnormalities, dyslipidemia, inflammation, and altered body composition may contribute to long-term cardiovascular risk in childhood cancer survivors. A related randomized clinical trial also explored the effect of long-chain omega-3 PUFA supplementation on body composition in children with ALL, further supporting its potential role as part of supportive nutritional care in pediatric oncology [260].

From an oncological perspective, omega-3 PUFAs are also attractive because they may improve chemotherapy tolerability and potentially enhance antitumor efficacy in certain settings. Experimental and clinical evidence suggests that DHA and EPA can modulate tumor cell membrane composition, oxidative susceptibility, inflammatory signaling, and chemotherapy response. Earlier experimental work showed that AIC was not increased by dietary omega-3 fatty acids, supporting their potential safety as adjunctive agents during anthracycline exposure [261]. However, the impact of omega-3 supplementation on tumor response, relapse risk, and long-term survival remains insufficiently established in contemporary cardio-oncology trials.

Despite these promising findings, several limitations warrant consideration. First, most mechanistic evidence is derived from preclinical models, whereas clinical data remain limited. Second, substantial heterogeneity in omega-3 formulations, including the EPA-to-DHA ratio, dose, chemical form, purity, oxidation status, and treatment duration, restricts direct comparison across studies. Third, although the pediatric ALL trial provides encouraging evidence of reduced OS and early biochemical or functional cardiac injury, larger, adequately powered multicenter randomized trials are required to confirm these findings and determine whether omega-3 supplementation improves clinically meaningful long-term outcomes, including persistent abnormalities in myocardial strain, decline in LVEF, symptomatic heart failure, and major cardiovascular events. Finally, safety assessments should account for gastrointestinal intolerance, hypersensitivity to fish-derived products, product purity and contamination, susceptibility to lipid oxidation, and the potential for increased bleeding risk at high doses, particularly in patients receiving concomitant anticoagulant or antiplatelet therapy [248].

Overall, omega-3 PUFAs represent one of the most promising nutritional strategies for prevention of OS-induced cardiotoxicity, particularly in pediatric patients receiving anthracycline-based chemotherapy. Their cardioprotective potential is supported by mechanistic evidence involving reduced mitochondrial ROS generation, improved antioxidant status, lower lipid and protein oxidation, anti-inflammatory lipid mediator production, and preservation of myocardial function. Among available nutritional interventions, omega-3 fatty acids are notable because they have direct randomized clinical evidence in children with ALL receiving DOX. Nevertheless, before routine implementation in cardio-oncology practice, further clinical trials should define optimal dose, EPA/DHA composition, timing of administration, duration of therapy, interaction with anticancer efficacy, and long-term cardiovascular outcomes. Careful consideration of PUFA type, dose, timing, and patient-specific factors is essential. Future research should clarify optimal omega-3 PUFA formulations and dosing regimens for cardioprotection without compromising antitumor efficacy.

### 8.3. Coenzyme Q10 and Mitochondria-Targeted Antioxidants

Coenzyme Q10 (CoQ10; ubiquinone) is an endogenously synthesized lipid-soluble benzoquinone localized predominantly within the inner mitochondrial membrane. It functions as an essential electron carrier between complexes I/II and complex III of the respiratory chain and, in its reduced form ubiquinol, acts as a membrane antioxidant capable of limiting lipid peroxidation. Because anthracyclines disrupt mitochondrial electron transport and increase mitochondrial ROS production, CoQ10 has long been proposed as a biologically plausible cardioprotective agent during DOX-containing chemotherapy [262]. Mechanistically, CoQ10 may attenuate AIC through several complementary actions. First, by supporting electron transfer within the respiratory chain, it may help preserve oxidative phosphorylation and ATP generation. Second, ubiquinol can reduce lipid peroxidation within mitochondrial and sarcolemmal membranes. Third, CoQ10 may stabilize mitochondrial membrane integrity and reduce propagation of ROS-dependent injury. Finally, by improving mitochondrial redox homeostasis, it may indirectly attenuate downstream activation of apoptotic, inflammatory, and fibrotic pathways [262,263].

Early clinical evidence for CoQ10 in anthracycline cardioprotection is limited but noteworthy. Iarussi et al. conducted a controlled study in children with ALL and non-Hodgkin lymphoma receiving anthracyclines and reported a protective effect of CoQ10 on cardiac function [264]. This study is particularly relevant to pediatric cardio-oncology because it directly examined CoQ10 in children exposed to anthracycline therapy. However, it was small; methodologically limited by contemporary standards; and preceded the current era of strain imaging, cardiac troponins, natriuretic peptides, and standardized cardio-oncology surveillance.

More recently, Greenlee et al. [265] performed a phase I randomized, placebo-controlled, cross-over dose-finding study of CoQ10 in women with stage I–III breast cancer receiving DOX plus cyclophosphamide. The primary objective was not cardioprotection, but rather evaluation of safety, tolerability, and potential pharmacokinetic interaction with DOX CoQ10 at 300 mg/day significantly increased serum CoQ10 concentrations and was well tolerated, with no clinically significant effect on DOX pharmacokinetics, total antioxidant capacity, or adverse events. This study is important because it directly addresses a major concern in cardio-oncology: whether antioxidant supplementation may alter anthracycline exposure. Nevertheless, it was very small and not designed to determine whether CoQ10 prevents cardiac dysfunction.

Taken together, CoQ10 has strong mechanistic plausibility and encouraging preclinical support, but its clinical efficacy for prevention of AIC remains insufficiently established. Future studies should evaluate standardized CoQ10 formulations, baseline CoQ10 status, dose–response relationships, pharmacokinetics, cardiac troponins, natriuretic peptides, GLS, cardiac magnetic resonance markers, and long-term cardiovascular outcomes. Importantly, trials should also monitor oncological endpoints to ensure that supplementation does not compromise antitumor efficacy.

Mitochondria-targeted antioxidants represent a promising evolution of CoQ10-based cardioprotection. Unlike conventional antioxidants, mitochondria-targeted antioxidants are designed to accumulate within mitochondria, thereby neutralizing ROS close to their major site of production. Mitoquinone (MitoQ) consists of a ubiquinone antioxidant moiety linked to a lipophilic triphenylphosphonium cation, which enables its accumulation within the mitochondrial matrix at concentrations several hundred-fold higher than untargeted antioxidants driven by the negative mitochondrial membrane potential. SKQ1 is another plastoquinone-derived mitochondria-targeted antioxidant with a similar conceptual rationale. These compounds are especially relevant in DOX cardiotoxicity because mitochondrial ROS generation is a proximal driver of downstream cardiomyocyte injury. Sacks et al. [266] directly compared mitochondrial antioxidants with vitamin C in a DOX-exposed H9c2 cardiomyoblast model. They found that MitoQ and SKQ1, but not vitamin C, mitigated DOX-induced cellular damage and that pretreatment with MitoQ provided greater protection than simultaneous co-treatment. This study is mechanistically important because it suggests that the subcellular localization and timing of antioxidant intervention may be more relevant than nonspecific systemic antioxidant capacity. In other words, protection against DOX cardiotoxicity may require early mitochondrial conditioning rather than delayed neutralization of cytosolic ROS.

MitoQ has also been investigated in the vascular component of anthracycline toxicity. Clayton et al. showed that DOX-induced OS and endothelial dysfunction were mediated, at least in part, by mitochondrial ROS and that endothelial function could be rescued by MitoQ [267]. This finding is highly relevant because cancer therapy-related cardiotoxicity is not restricted to cardiomyocytes; endothelial dysfunction, microvascular injury, and impaired nitric oxide bioavailability contribute to long-term cardiovascular risk, particularly in patients exposed to anthracyclines and radiotherapy. A recent study by Abdeahad et al. extended this concept by showing that MitoQ reduced senescence burden in DOX-treated endothelial cells through the attenuation of mitochondrial ROS and DNA damage [268]. This observation is particularly important for long-term survivorship because endothelial senescence may contribute to vascular aging, impaired vasodilatory capacity, chronic inflammation, and accelerated cardiovascular disease after cancer therapy. Thus, MitoQ may have relevance not only for acute cardiomyocyte protection but also for prevention of vascular aging and delayed cardiovascular complications. Despite these promising findings, MitoQ and SKQ1 remain predominantly experimental in the context of AIC. No adequately powered randomized clinical trials have established their efficacy for prevention of cancer therapy-related cardiac dysfunction. Moreover, because their uptake depends on mitochondrial membrane potential, their pharmacodynamics may vary across healthy cardiomyocytes, injured myocardium, endothelial cells, and cancer cells. Future studies should therefore assess tissue specificity, timing of administration, potential effects on tumor biology, and long-term cardiovascular safety.

Because the clinical translation of CoQ10 is limited by poor oral bioavailability and instability, nanoformulation strategies are particularly relevant. Quagliariello et al. [269] developed CoQ10-loaded secondary and tertiary nanoemulsions and tested them in human cardiomyocytes and hepatocytes exposed to DOX and trastuzumab. CoQ10-loaded nanocarriers improved cell viability and reduced lipid peroxidation, ROS generation, leukotriene B4, p65/NF-κB expression, and IL-1β and IL-6 production. These findings suggest that nanoencapsulation may enhance the cardio- and hepatoprotective effects of CoQ10 by simultaneously targeting OS and inflammatory signaling during exposure to cardiotoxic anticancer therapies [269].

CoQ10 and mitochondria-targeted antioxidants are conceptually attractive because they address a proximal mechanism of OS-induced cardiotoxicity: mitochondrial ROS generation and bioenergetic failure. However, their translational readiness differs substantially. CoQ10 has the longest history of human use and limited clinical evidence in anthracycline-treated patients, including pediatric and breast cancer settings, but high-quality efficacy trials remain lacking. MitoQ and SKQ1 have stronger mechanistic specificity for mitochondrial ROS or cardiolipin stabilization, but their evidence in cancer therapy-related cardiotoxicity is mainly preclinical. Several issues must be resolved before these interventions can be integrated into cardio-oncology practice. First, timing may be critical, as mitochondrial antioxidants appear more effective when administered before or early during DOX exposure rather than after irreversible injury has occurred. Second, not all antioxidants are equivalent; mitochondrial targeting may provide advantages over conventional cytosolic antioxidants. Third, oncological safety must be carefully evaluated because antioxidant strategies could theoretically alter chemotherapy-induced oxidative tumor cell injury. Fourth, clinical trials should assess not only LVEF but also GLS, cardiac biomarkers, endothelial function, mitochondrial biomarkers, quality of life, and long-term cardiovascular events.

### 8.4. Selenium

Selenium is an essential trace element with a central role in redox regulation, antioxidant defense, thyroid hormone metabolism, immune function, and cardiovascular homeostasis. Its biological activity is mediated mainly through its incorporation into selenoproteins, several of which are directly involved in the control of OS. Among these, GPx, thioredoxin reductases (TXNRD), selenoprotein P, and other redox-active selenoproteins are particularly relevant for the myocardium, where continuous mitochondrial oxidative metabolism requires efficient antioxidant buffering. Because AIC is strongly associated with mitochondrial ROS generation, lipid peroxidation, inflammation, apoptosis, ferroptosis, and progressive ventricular remodeling, selenium has been proposed as a potentially useful micronutrient for nutritional cardioprotection. The mechanistic rationale for selenium supplementation in DOX-induced cardiotoxicity is based largely on its capacity to reinforce endogenous antioxidant systems rather than on direct radical scavenging. Selenium is required for the activity of GPx enzymes, which reduce hydrogen peroxide and lipid hydroperoxides, thereby limiting propagation of oxidative membrane damage. It is also essential for TXNRD activity, which maintains thioredoxin-dependent redox signaling and contributes to mitochondrial redox homeostasis. In experimental models of oxidative myocardial stress, selenium supplementation increased GPx and TXNRD activity, attenuated OS-induced cardiomyocyte cell cycle arrest, and activated redox-sensitive PI3K/AKT signaling [270]. These findings provide a broader mechanistic basis for selenium-mediated cardioprotection, suggesting that selenium may preserve cardiomyocyte viability by maintaining selenoprotein-dependent antioxidant capacity and redox-sensitive survival pathways.

Direct preclinical evidence supports a cardioprotective role for selenium in AIC. In a murine model of DOX cardiotoxicity, selenium supplementation attenuated myocardial dysfunction, reduced circulating biomarkers of cardiac injury, limited oxidative damage and pro-inflammatory cytokine expression in myocardial tissue, enhanced Nrf2 signaling, and suppressed activation of the NLRP3 inflammasome [271]. Importantly, inhibition of Nrf2 abolished the protective effects of selenium and diminished its suppression of NLRP3 activation, indicating that the Nrf2–NLRP3 axis may be a key mechanistic link between selenium-dependent antioxidant defense and reduced inflammatory cardiac injury. This is highly relevant because both impaired Nrf2 signaling and NLRP3 inflammasome activation are increasingly recognized as contributors to OS-induced cardiotoxicity, pyroptosis, and adverse cardiac remodeling. Earlier experimental studies also support the involvement of selenium-dependent antioxidant mechanisms in anthracycline-induced cardiac injury. In a rat model of delayed DOX cardiotoxicity, dietary selenium supplementation attenuated myocardial damage, with the findings implicating phospholipid hydroperoxide glutathione peroxidase, now designated GPX4, rather than conventional cytosolic GPx activity alone. By reducing membrane phospholipid hydroperoxides, GPX4 may limit lipid peroxidation and preserve cardiomyocyte membrane integrity [272]. This distinction is important because DOX-induced cardiotoxicity involves peroxidation of mitochondrial and sarcolemmal phospholipids, including cardiolipin-rich mitochondrial membranes. Therefore, selenium-dependent enzymes that reduce lipid hydroperoxides may be particularly relevant for preventing membrane destabilization, mitochondrial dysfunction, and downstream cardiomyocyte death.

Nanotechnology-based selenium formulations have further broadened the range of potential cardioprotective strategies. In a murine model, selenium-silica nanocomposites attenuated DOX-induced cardiotoxicity while preserving the antitumor activity of DOX. Treatment was associated with improved survival, reduced histopathological myocardial injury and apoptosis, preservation of LVEF, and attenuation of cardiac ROS generation [273]. In more recent study, selenium and iron oxide nanoparticles, alone and particularly in combination, mitigated DOX-induced cardiomyopathy in rats. DOX increased CK-MB, troponin I, NADPH oxidase, MDA, inflammatory mediators, and matrix metalloproteinase-9, while reducing SOD and GPx. Selenium-based nanoparticle treatment improved OS, inflammatory, and apoptotic markers, suggesting that selenium nanoformulations may provide more efficient redox modulation than conventional supplementation in selected experimental settings [274]. Biogenically synthesized selenium nanoparticles conjugated with DOX have also been investigated as a strategy for reducing the systemic toxicity of anthracycline therapy. In a murine model, the DOX–selenium nanoparticle conjugate attenuated oxidative and nitrosative stress, genotoxicity, and tissue injury in the heart, liver, and kidneys. These effects were associated with reduced ROS and RNS generation, decreased MDA and 8-hydroxy-2′-deoxyguanosine levels, restoration of endogenous antioxidant enzyme activities, preservation of tissue architecture, and attenuation of DNA damage. Increased BCL-2 expression further suggested suppression of mitochondria-mediated apoptotic signaling [275]. This study is particularly relevant because it suggests that selenium nanoparticles may simultaneously reduce OS, DNA damage, and apoptosis-related injury during DOX exposure, although further studies are required to confirm whether such formulations preserve antitumor efficacy and are safe in clinical settings.

Selenium may also be relevant to radiation-induced cardiac injury. Radiotherapy-induced cardiotoxicity is characterized by persistent ROS generation, endothelial dysfunction, DNA damage, immune dysregulation, and progressive fibrosis. Experimental work using fungus-based oral selenium microcarriers (a novel bioactive *Cordyceps militaris*-based Se oral delivery system (Se@CM)) showed protection against high-dose X-ray-induced heart injury by reducing ROS overproduction, alleviating DNA damage, and modulating immune responses [276]. Although this evidence is still preclinical and not specific to conventional thoracic radiotherapy protocols, it supports the broader concept that selenium-based strategies may have relevance in both anthracycline- and radiation-associated oxidative cardiac injury.

Clinical evidence for selenium in anthracycline cardioprotection is limited but notable. Tacyildiz et al. investigated the potential protective effect of selenium supplementation in a group of 67 pediatric cancer patients receiving anthracyclines and reported an association between low selenium levels and AIC, with findings suggesting that selenium supplementation may help prevent cardiac toxicity [277]. This study is important because it directly addresses a pediatric oncology population, which is highly relevant given the long-term cardiovascular vulnerability of childhood cancer survivors. However, the evidence should be interpreted cautiously because the study was relatively small and did not use contemporary cardio-oncology endpoints such as GLS, high-sensitivity troponins, serial natriuretic peptides, or cardiac MRI.

The potential clinical role of selenium in oncology remains complex. A recent systematic review of selenium as complementary treatment in cancer patients concluded that selenium has been investigated across several malignancies and treatment settings, but the evidence remains heterogeneous with respect to cancer type, selenium formulation, baseline selenium status, dose, endpoints, and safety [278]. This heterogeneity is especially relevant in cardio-oncology, where supplementation should ideally target patients with low or insufficient selenium status rather than applied indiscriminately. Both selenium deficiency and excessive selenium exposure may be harmful, and high-dose selenium supplementation can cause toxicity, including gastrointestinal symptoms, hair and nail changes, neurological symptoms, and metabolic disturbances. Another important translational issue is oncological safety. Since OS contributes not only to cardiotoxicity but also to the cytotoxic action of some anticancer treatments, antioxidant micronutrients must be evaluated for their possible effects on tumor response. The interaction between selenium and DOX may be context-, dose-, formulation-, and tumor-dependent. Some experimental data suggest that selenium-dependent modulation of GPx activity can influence DOX-induced tumor cell cytotoxicity and hydroxyl radical production [279]. An important cautionary aspect of selenium supplementation relates to its potential impact on the oxidative mechanisms of anticancer therapy. Gencheva et al. [280] investigated the prooxidant combination of ascorbate and menadione sodium bisulfite (VC/VK3; Apatone^®^) in human glioblastoma and non-transformed glial cell lines. The authors showed that VC/VK3 induced cancer cell toxicity in an H_2_O_2_- and iron-dependent manner, suggesting the involvement of ferroptosis-like oxidative cell death. However, selenium supplementation significantly protected cancer cells from VC/VK3-induced OS and cytotoxicity by increasing the expression and enzymatic activity of antioxidant selenoproteins, including thioredoxin reductase and GPx. Inhibition of thioredoxin reductase or glutathione-dependent antioxidant systems enhanced VC/VK3 toxicity, indicating that selenoprotein-mediated antioxidant defenses can contribute to resistance against prooxidant anticancer strategies [280]. Although this study was not performed in a DOX cardiotoxicity model, it is highly relevant for cardio-oncology because it emphasizes that selenium-based cardioprotection must be evaluated together with oncological efficacy, particularly when anticancer activity depends on OS or ferroptosis-related mechanisms. Therefore, selenium should not be presented as a universally protective supplement during chemotherapy but rather as a biologically plausible intervention requiring careful dose optimization, formulation selection, and simultaneous assessment of cardiovascular and oncological outcomes. current evidence is insufficient to recommend routine selenium supplementation for all patients receiving anthracyclines. Future studies should stratify patients according to baseline selenium status, define optimal selenium species and dose, assess cardiac biomarkers and strain-based imaging, monitor long-term cardiovascular outcomes, and carefully evaluate possible interactions with antitumor efficacy.

### 8.5. Vitamins

#### 8.5.1. Vitamin C

Vitamin C, or ascorbic acid, is a water-soluble antioxidant with important roles in redox homeostasis; collagen biosynthesis; endothelial function; immune regulation; and regeneration of other antioxidants, including vitamin E. In the context of AIC, vitamin C has attracted attention because DOX promotes ROS and RNS generation, mitochondrial dysfunction, lipid peroxidation, protein oxidation, DNA damage, inflammation, and cardiomyocyte death. A recent review summarized the available preclinical and clinical evidence and concluded that vitamin C has biologically plausible cardioprotective potential against DOX-induced cardiotoxicity, although clinical evidence remains limited and heterogeneous [281]. Mechanistically, vitamin C may protect the myocardium through several complementary pathways. First, ascorbate can directly neutralize aqueous ROS and limit propagation of oxidative injury. Second, it can regenerate oxidized vitamin E and thereby indirectly support membrane antioxidant defense. Third, vitamin C may reduce nitrosative stress by modulating nitric oxide synthase activity and nitric oxide-related pathways. Fourth, by lowering OS, it can attenuate activation of redox-sensitive signaling cascades, including p38 MAPK, JNK, p53, NF-κB, and inflammatory cytokine pathways [282]. These mechanisms are particularly relevant in DOX cardiotoxicity, where oxidative/nitrosative stress acts upstream of mitochondrial dysfunction, apoptosis, inflammation, and adverse cardiac remodeling [281].

Several preclinical studies support this mechanistic rationale. Swamy et al. showed that ascorbic acid protected against DOX-induced myocardial injury in rats by increasing endogenous antioxidant defenses, including reduced glutathione, SOD, and catalase, while decreasing MDA, CK, LDH, AST, and ALT [283]. These findings suggest that vitamin C can reduce lipid peroxidation and preserve antioxidant enzyme activity during anthracycline exposure. Importantly, both preventive and post-treatment administration improved OS parameters, although preventive use may be more relevant for nutritional cardioprotection. Akolkar et al. [284] provided particularly important in vivo evidence linking vitamin C to suppression of oxidative/nitrosative stress, inflammation, apoptosis, and structural cardiac injury. In a rat model of DOX-induced cardiomyopathy, vitamin C improved cardiac structure and function, reduced oxidative and nitrosative stress, attenuated inflammatory activation, and limited apoptotic signaling. This study is highly relevant because it connects biochemical redox modulation with functional and structural myocardial protection, supporting the concept that vitamin C may interfere with multiple downstream consequences of DOX-induced ROS/RNS generation. Cellular studies have further clarified the subcellular basis of vitamin C-mediated cardioprotection. The cellular time-course study by Ludke et al. [285] further clarified the sequence of molecular events involved in vitamin C-mediated protection. In isolated adult rat cardiomyocytes, DOX induced a time-dependent increase in ROS production, followed by activation of p53, p38, and JNK MAPK signaling; autophagy markers; apoptotic signaling; and loss of cell viability. Vitamin C reduced ROS generation at all examined time points and blunted activation of these stress-induced pathways, thereby preventing apoptosis and preserving cardiomyocyte viability [285]. This work supports the concept that vitamin C may be most effective when present early during DOX exposure, before OS-dependent signaling cascades become amplified.

A key issue in cardio-oncology is whether antioxidant vitamins might interfere with antitumor efficacy. Older experimental data suggested that vitamins C and E can protect normal cells against DOX-induced radical injury without necessarily reducing the cytotoxic effect of DOX against tumor cells [285]. In addition, some in vitro studies showed that ascorbic acid enhanced the antineoplastic activity of DOX, cisplatin, and paclitaxel in human breast carcinoma cells [286]. However, these findings cannot be directly extrapolated to clinical oncology because the biological effects of vitamin C depend strongly on concentration, route of administration, redox environment, tumor type, iron availability, and concurrent treatment. At physiological concentrations, vitamin C acts mainly as an antioxidant, whereas pharmacological intravenous ascorbate may exert prooxidant effects through hydrogen peroxide generation in the extracellular tumor microenvironment. The potential oncological interaction between vitamin C and anthracyclines is particularly important. Bober et al. [287] used proteomic analysis in MCF-7 breast cancer cells and showed that vitamin C potentiated the antiproliferative effect of DOX, with the strongest effect observed when 200 µM vitamin C was combined with 1 µM DOX. The combined treatment altered the expression of proteins involved in structural organization, transcription, translation, immune processes, antioxidant responses, cellular signaling, and transport [287]. This finding supports the hypothesis that vitamin C may not necessarily compromise DOX efficacy and may, in some cellular contexts, enhance its antitumor activity. Similarly, Perveen et al. [288] investigated interactions between anthracyclines, DNA, and ascorbic acid using electrochemical, spectroscopic, theoretical, and cancer cell line approaches. The authors found that ascorbic acid enhanced anthracycline–DNA interactions under physiological conditions and increased the inhibitory effects of anthracyclines against non-small cell lung cancer cell lines [288]. Although this study is not a cardiotoxicity model, it is useful because it addresses a key cardio-oncology concern: whether vitamin C could interfere with anthracycline antitumor activity. These data suggest that vitamin C may, under certain conditions, enhance rather than reduce anthracycline cytotoxicity.

However, not all antioxidant strategies are equivalent. Sacks et al. [266] compared vitamin C with the mitochondria-targeted antioxidants MitoQ and SKQ1 in H9c2 cardiomyoblasts exposed to DOX. MitoQ and SKQ1, but not vitamin C, significantly mitigated DOX-induced cellular injury, with MitoQ pretreatment showing the strongest protection. The protective effect was associated with reductions in both intracellular and mitochondrial OS [266]. This study is important because it suggests that mitochondrial localization of antioxidant activity may be more relevant than nonspecific aqueous antioxidant capacity in DOX cardiotoxicity.

Recent experimental work has expanded the discussion toward combined metabolic and antioxidant strategies. Al-Jada et al. investigated the effects of ketogenic diet and high-dose vitamin C on DOX toxicity in a murine breast cancer model [289]. This study is relevant to precision nutrition because it evaluates vitamin C not as an isolated antioxidant but as part of a metabolic intervention that may influence both tumor biology and chemotherapy-associated toxicity. Nevertheless, such approaches should be interpreted cautiously until their effects on cardiac outcomes, OS markers, tumor response, and systemic toxicity are more clearly established.

Despite promising preclinical evidence, clinical data specifically supporting vitamin C for prevention of AIC remain insufficient. The 2025 review by Nsairat et al. emphasized that available clinical evidence is variable and that further studies are required to define optimal dose, administration route, timing relative to DOX administration and preexisting cardiovascular conditions, and cancer-type influencing effectiveness [281]. This limitation is important because many preclinical studies use controlled experimental conditions and dosing regimens that may not reflect human pharmacokinetics. Moreover, modern cardio-oncology trials should evaluate high-sensitivity troponins, natriuretic peptides, GLS, cardiac MRI, OS biomarkers, and long-term cardiovascular outcomes rather than relying only on conventional serum enzymes or histopathology. Unlike omega-3 fatty acids or nanocurcumin, vitamin C currently lacks robust randomized clinical evidence demonstrating prevention of anthracycline-related cardiac dysfunction. Therefore, vitamin C should be discussed as a promising but not yet clinically established cardioprotective strategy. Future clinical trials should determine whether vitamin C supplementation, particularly when optimized for timing, dose, and route of administration, can safely reduce OS-induced myocardial injury without compromising oncological efficacy. The oncological studies are reassuring because they suggest that vitamin C potentially can enhance DOX cytotoxicity in some tumor models, but this remains context-dependent and requires confirmation in vivo and in clinical trials.

Direct evidence for vitamin C alone in the prevention of radiation-induced myocardial injury remains limited. Nevertheless, early clinical evidence suggests that antioxidant combinations containing vitamin C may warrant further investigation. In a small prospective, randomized, double-blind, placebo-controlled pilot study of patients receiving high-dose chemotherapy or radiotherapy, Wagdi et al. [290] evaluated a combined regimen of vitamins C and E together with N-acetylcysteine. Among patients undergoing radiotherapy, LVEF remained unchanged in the antioxidant-treated group, whereas a significant decline was observed in the placebo group. Importantly, because the intervention combined three antioxidants and included only a small number of patients, the contribution of vitamin C itself cannot be determined and the findings should be regarded as hypothesis-generating rather than confirmatory [290].

#### 8.5.2. Vitamin E

Vitamin E is a group of lipid-soluble compounds that includes tocopherols and tocotrienols, with α-tocopherol being the most biologically active and most extensively investigated form in humans. In contrast to vitamin C, which primarily acts in aqueous compartments, vitamin E is localized within lipid membranes, where it functions as a chain-breaking antioxidant. By donating a hydrogen atom to lipid peroxyl radicals, α-tocopherol interrupts the propagation phase of lipid peroxidation and helps preserve the integrity of polyunsaturated fatty acid-rich cellular and mitochondrial membranes [291]. This mechanism is highly relevant to DOX-induced cardiotoxicity, in which mitochondrial ROS generation, cardiolipin oxidation, lipid peroxidation, and membrane damage contribute to cardiomyocyte dysfunction and death.

The cardioprotective rationale for vitamin E is therefore based mainly on its capacity to limit lipid peroxidation and membrane oxidative damage. Vitamin E may also interact with other antioxidant systems, particularly vitamin C, which can regenerate α-tocopherol from the tocopheroxyl radical and thereby restore its chain-breaking antioxidant activity [292,293]. This antioxidant network could be useful during anthracycline exposure, where OS affects both aqueous and lipid compartments. However, the translation of this mechanistic rationale into consistent cardioprotection has been challenging.

Several preclinical studies have reported beneficial effects of vitamin E in DOX-induced toxicity. Geetha et al. showed that α-tocopherol reduced DOX-induced histological injury and lipid peroxidation in multiple organs, including the heart, while normalizing serum enzyme markers of tissue damage [294]. Puri et al. demonstrated that vitamin E pretreatment improved electrocardiographic abnormalities induced by DOX, including PR, QT, and ST segment changes, and reduced biochemical markers of myocardial injury such as CK-MB and LDH [295]. Hadi et al. also reported that vitamin E, alone or in combination with telmisartan, attenuated acute DOX-induced cardiac injury in rats through antioxidant and anti-inflammatory mechanisms [296]. These findings support the concept that vitamin E can reduce acute oxidative myocardial injury in selected experimental models. The strongest mechanistic rationale comes from the ability of α-tocopherol to interrupt lipid peroxidation within cellular and mitochondrial membranes. However, Berthiaume et al. showed that dietary α-tocopherol succinate enriched cardiac mitochondrial membranes and reduced oxidative protein damage but failed to prevent DOX-induced mitochondrial dysfunction and cardiac histopathological injury [297]. This is an important limitation because it suggests that reducing oxidative markers alone may be insufficient to prevent chronic AIC. It also supports the broader view that DOX cardiotoxicity is not mediated exclusively by nonspecific OS but also involves mitochondrial iron accumulation, TOP2β injury, impaired mitochondrial biogenesis, calcium dysregulation, ferroptosis, apoptosis, inflammation, and fibrotic remodeling.

Combination chemotherapy may further modify the effects of vitamin E. Bjelogrlic et al. reported that a single oral dose of dl-α-tocopherol failed to inhibit acute DOX cardiotoxicity but delayed progression of cardiac injury; however, vitamin E did not protect against cardiotoxicity induced by the combination of DOX and cyclophosphamide [298]. This finding is particularly relevant for clinical oncology because anthracyclines are commonly administered as part of multidrug regimens rather than as isolated agents. Therefore, antioxidant efficacy observed in simplified DOX-only models may not necessarily translate to combination chemotherapy protocols.

Clinical evidence remains limited and inconclusive. A scoping review by Moustafa et al. found that vitamin E trials in adult cancer patients receiving DOX were inconsistent and inconclusive, whereas levocarnitine trials showed more consistent signals of cardioprotection; importantly, no previous human trials had evaluated the combination of vitamin E and levocarnitine before their subsequent prospective study [299]. More recently, the same group conducted a prospective randomized controlled study in adult women with breast cancer receiving DOX and cyclophosphamide. Combined prophylaxis with vitamin E and levocarnitine was associated with lower BNP and CK changes and fewer cardiac events compared with control treatment [300]. However, because vitamin E was administered together with levocarnitine, the independent contribution of vitamin E cannot be determined. In addition, longer follow-up and modern cardio-oncology endpoints, including high-sensitivity troponins, GLS, cardiac MRI, and long-term heart failure outcomes, are still needed. Recent work has shifted attention from conventional vitamin E supplementation toward vitamin E-derived drug-delivery systems. Pandurangi et al. [301] synthesized targeted and enzyme-cleavable vitamin E analogs intended to sensitize tumor cells and reduce the cardiotoxic burden of DOX. In preclinical models, these analogs synergized with DOX, improved the therapeutic index, and preserved ventricular function compared with cardiotoxic-dose DOX alone [301]. This study reframes vitamin E derivatives not merely as antioxidants but as tumor-directed adjuncts designed to improve anticancer efficacy while reducing cardiac exposure or toxicity. Similarly, several tocopheryl succinate-based nanocarriers have been developed to improve DOX pharmacokinetics, tumor delivery, and safety. Anbharasi et al. conjugated DOX to D-α-tocopheryl polyethylene glycol succinate and folic acid, showing increased cellular uptake, improved cytotoxicity in MCF-7 cells, prolonged systemic exposure, and reduced cardiac drug accumulation in vivo [302]. Boratto et al. showed that α-tocopheryl succinate improved encapsulation, pH-sensitive release, and antitumor activity of DOX-loaded nanostructured lipid carriers [303]. Lages et al. further developed a pH-sensitive DOX-tocopherol succinate prodrug encapsulated in DHA-based nanostructured lipid carriers, which improved pharmacokinetics, reduced QT prolongation and left ventricular systolic dysfunction, and decreased cardiac and hepatic toxicity while maintaining antitumor efficacy in 4T1 tumor-bearing mice [304]. Together, these studies suggest that tocopherol-based structures may be more useful as components of targeted drug-delivery systems than as conventional antioxidant supplements.

Other nanomedicine studies support the same translational concept. Cagel et al. used mixed micelles including D-α-tocopheryl polyethylene glycol succinate for DOX encapsulation and reported enhanced in vitro cytotoxicity against breast and ovarian cancer cells compared with Doxil^®^ [305]. Metwally et al. developed cationic D-α-tocopheryl polyethylene glycol 1000 succinate (TPGS) mixed micelles of berberine and reported mitigation of DOX-induced cardiotoxicity through attenuation of mitochondrial dysfunction and apoptosis [306]. These studies are useful for the broader argument that vitamin E derivatives and TPGS can function as pharmacological excipients or bioactive delivery platforms that influence drug distribution, mitochondrial injury, and apoptotic signaling.

Another key issue is oncological safety. Because part of antitumor activity of DOX may involve OS and redox-dependent signaling, high-dose antioxidant supplementation could theoretically interfere with chemotherapy efficacy. The available evidence is mixed. Ahmadi et al. reported that vitamin E enhanced the cytotoxic effect of DOX in human breast cancer cell lines while showing lower toxicity toward normal fibroblasts, suggesting that vitamin E may not necessarily impair antitumor activity in all contexts [307]. Similarly, de Oliveira et al. found that α-tocopherol reduced genotoxic damage induced by DOX and cyclophosphamide in non-tumor models while showing selective cytotoxicity toward tumor cells [308]. In contrast, Diao et al. reported that vitamin E promoted breast cancer cell proliferation by reducing ROS production and p53 expression [309]. These conflicting findings indicate that the effect of vitamin E on tumor biology may depend on cancer type, p53 status, dose, formulation, redox state, and treatment context.

Overall, vitamin E is a biologically plausible cardioprotective antioxidant because it directly targets lipid peroxidation within cellular and mitochondrial membranes. Preclinical studies show that α-tocopherol can reduce DOX-induced lipid peroxidation, improve antioxidant status, attenuate electrocardiographic abnormalities, and reduce biochemical markers of myocardial injury. However, other studies demonstrate that vitamin E may reduce oxidative damage without preventing mitochondrial dysfunction or chronic cardiac remodeling. Clinical evidence is limited, heterogeneous, and insufficient to support routine vitamin E supplementation for prevention of AIC. Future trials should evaluate vitamin E as part of a clearly defined antioxidant strategy, with standardized doses and formulations, careful assessment of baseline nutritional status, modern cardiac imaging and biomarker endpoints, and parallel monitoring of oncological outcomes. Vitamin E derivatives such as TPGS and tocopheryl succinate appear more promising as components of targeted prodrugs and nanocarriers designed to reduce cardiac exposure to DOX, improve tumor delivery, and preserve or enhance antitumor efficacy. Therefore, vitamin E should be presented cautiously: not as an established cardioprotective supplement but as a mechanistically plausible antioxidant whose greatest translational potential may lie in tocopherol-based drug-delivery systems.

Vitamin E has also been investigated in experimental radiation-induced cardiac injury. Atasoy et al. [310] evaluated α-tocopherol pretreatment in rats exposed to 20 Gy irradiation and assessed early oxidative and inflammatory changes in cardiac and pulmonary tissues. Irradiation increased cardiac myeloperoxidase activity, whereas vitamin E pretreatment was associated with reduced neutrophil infiltration; however, changes in myocardial malondialdehyde and chemiluminescence-based oxidative stress indices were not significantly improved. These findings suggest a potential early anti-inflammatory effect but provide only limited evidence for direct myocardial radioprotection [310]. Supportive clinical evidence is provided by the small randomized pilot study of Wagdi et al. [290], in which a combined antioxidant regimen containing vitamins E and C together with N-acetylcysteine was associated with preservation of LVEF in patients undergoing radiotherapy. Nevertheless, the small sample size and combined intervention preclude attribution of the observed effect specifically to vitamin E [290].

#### 8.5.3. Vitamin D

Vitamin D is a lipid-soluble secosteroid that functions primarily as a hormone-like regulator of calcium-phosphate metabolism, immune responses, inflammation, endothelial function, and cardiovascular homeostasis. Although it is often discussed together with antioxidant vitamins, vitamin D should not be regarded as a classical chain-breaking antioxidant comparable to vitamin E or as a direct aqueous radical scavenger comparable to vitamin C. Instead, its potential cardioprotective effects are mediated mainly through vitamin D receptor (VDR)-dependent transcriptional regulation; modulation of inflammatory signaling; preservation of mitochondrial function; attenuation of OS; and inhibition of profibrotic remodeling pathways including NF-κB, Nrf2, PI3K/AKT, AMPK, and TGF-β/Smad [311,312].

This mechanism is highly relevant to AIC, where OS, mitochondrial dysfunction, inflammation, endothelial injury, and fibrosis collectively drive myocardial damage. Experimental studies have demonstrated that vitamin D and calcitriol attenuate DOX-induced ROS production, preserve mitochondrial integrity, suppress inflammatory cytokine signaling, inhibit endothelial-to-mesenchymal transition and myocardial fibrosis, and improve cardiac function [313,314,315]. Preclinical evidence provides important mechanistic support for this concept. Lee et al. [313] showed in a triple-negative breast cancer mouse model that vitamin D supplementation reduced DOX-induced cardiotoxicity by decreasing ROS production and mitochondrial damage. Importantly, vitamin D did not reduce the anticancer efficacy of DOX against triple-negative breast cancer, which is highly relevant for cardio-oncology because any cardioprotective intervention must preserve oncological efficacy [313]. This study supports the view that vitamin D may improve the cardiac tolerance of anthracycline therapy through mitochondrial and redox-dependent mechanisms without necessarily compromising antitumor activity.

Calcitriol has also been shown to attenuate DOX-induced cardiac dysfunction and remodeling. Tsai et al. [316] demonstrated that calcitriol improved cardiac dysfunction and inhibited endothelial-to-mesenchymal transition in a mouse model of DOX cardiotoxicity. This is particularly important because endothelial-to-mesenchymal transition may contribute to myocardial fibrosis, microvascular dysfunction, and chronic ventricular remodeling after anthracycline exposure.

More recent mechanistic evidence links active vitamin D to the Nrf2–NLRP3 axis. Gu et al. [317] reported that 1,25(OH)_2_D_3_ ameliorated DOX-induced cardiomyopathy by improving cardiac function, reducing BNP and cTnT levels, suppressing myocardial fibrosis and inflammatory cytokine expression, and attenuating OS. Mechanistically, 1,25(OH)_2_D_3_ inhibited NLRP3 inflammasome activation and modulated Keap1–Nrf2-related OS signaling through changes in histone modifications, including H3K4me3, H3K27me3 and H2AK119Ub, at the NLRP3 and Nrf2 promoter regions [317]. This finding is relevant to the broader framework of OS-induced cardiotoxicity because Nrf2 activation supports endogenous antioxidant defense, whereas NLRP3 activation promotes inflammatory cytokine release, pyroptosis, and adverse remodeling. Thus, vitamin D may act as an epigenetically active regulator of antioxidant and inflammatory pathways rather than as a simple antioxidant molecule.

Additional animal studies further support the antioxidant and anti-inflammatory role of vitamin D in DOX cardiac injury. Koroglu et al. [318] showed that both paricalcitol, a vitamin D receptor agonist, and vitamin D_3_ protected against acute DOX cardiotoxicity in rats, based on electrocardiographic, biochemical, and scintigraphic findings, with antioxidant and anti-inflammatory mechanisms proposed as central contributors. Azizian et al. [319] further demonstrated that vitamin D ameliorated celecoxib-aggravated cardiotoxicity in a DOX heart failure rat model by enhancing antioxidant defenses and minimizing mitochondrial dysfunction. Awad et al. [320] showed that vitamin D attenuated DOX-induced cardiotoxicity in rats, with particular emphasis on calcium homeostasis. Since disruption of intracellular calcium handling contributes to contractile dysfunction, mitochondrial injury, and cardiomyocyte death during anthracycline exposure, this study supports the concept that vitamin D may protect the myocardium through mechanisms extending beyond antioxidant activity alone.

The clinical oncology literature also supports the broader relevance of vitamin D status, especially in lymphoid malignancies treated with immunochemotherapy regimens that may include DOX. In patients with diffuse large B-cell lymphoma (DLBCL), vitamin D deficiency has been associated with inferior clinical outcomes, including shorter progression-free and overall survival [321,322]. Wang et al. further reported that 25(OH)D deficiency was an independent prognostic factor for inferior progression-free and overall survival in newly diagnosed DLBCL and that c-MYC positivity was more frequent among patients with vitamin D deficiency [321]. Similarly, low serum vitamin D levels were associated with inferior survival in follicular lymphoma in prospective SWOG and LYSA cohorts [323]. These studies do not directly evaluate cardiotoxicity, but they indicate that vitamin D deficiency is clinically meaningful in oncology and may reflect a state of impaired immune, metabolic, and inflammatory regulation. Additional studies suggest that not only total 25(OH)D, but also bioavailable vitamin D, may be relevant. Chen et al. [322] reported that bioavailable 25(OH)D levels were associated with clinical outcomes in patients with DLBCL. Hohaus et al. [324] also investigated vitamin D deficiency and supplementation in patients with aggressive B-cell lymphomas treated with immunochemotherapy, further supporting the concept that vitamin D status should be considered in patients receiving intensive systemic therapy. From a cardio-oncology perspective, these data are important because lymphoma patients treated with R-CHOP-like regimens are exposed to both DOX and systemic inflammatory/metabolic stressors. Nevertheless, these studies should not be overinterpreted as proof that hypovitaminosis D directly increases susceptibility to AIC.

Clinical evidence remains limited but promising. El-Bassiouny et al. [315] investigated vitamin D in women with early breast cancer receiving adjuvant DOX-based chemotherapy. In this randomized clinical study, patients receiving vitamin D in addition to DOX/cyclophosphamide showed lower serum markers related to cardiotoxicity and inflammation, including cardiac troponin T, LDH, and interleukin-6 (IL-6), compared with controls [315]. This study is important because it provides direct clinical evidence that vitamin D may attenuate anthracycline-associated cardiac injury in breast cancer patients, possibly through suppression of IL-6-mediated inflammatory signaling. However, the study was relatively small and short-term, and more robust trials using high-sensitivity troponins, NT-proBNP, GLS, cardiac MRI, and long-term heart failure outcomes are still required.

A particularly important translational issue is whether vitamin D supplementation could interfere with, preserve, or enhance anticancer efficacy. Several studies suggest that vitamin D metabolites may exert antitumor or chemosensitizing effects in selected models. Zhang et al. [325] showed that calcitriol enhanced DOX-induced apoptosis in papillary thyroid carcinoma cells through regulation of the VDR/PTPN2/p-STAT3 pathway. Earlier experimental evidence also suggested that 1,25-dihydroxyvitamin D_3_ can increase the susceptibility of breast cancer cells to DOX [326]. Khriesha et al. [327] reported that different vitamin D metabolites showed potential anticancer activity in colorectal and breast cancer cell lines, either alone or in combination with chemotherapeutic agents. Attia et al. [328] linked vitamin D3 to mechanisms of chemoresistance in breast cancer involving aldehyde dehydrogenase ALDH1 and multidrug resistance pathways, whereas Tan et al. [329] showed that calcitriol and calcipotriol modulated the activity of ABC transporters, including P-glycoprotein, MRP-1, and BCRP, and inhibited MRP-1-mediated DOX efflux. These findings are relevant because they suggest that vitamin D analogues may influence intracellular drug accumulation and multidrug resistance. However, they also raise the possibility of altered pharmacokinetics, tissue distribution, or toxicity depending on tumor type, transporter expression, dose, sex, and vitamin D formulation. This concern is reinforced by experimental pharmacokinetic data. Corum and Uney [330] reported that calcitriol did not substantially alter plasma DOX pharmacokinetics but affected tissue distribution and excretion in a sex-dependent manner [330]. This study is important because it highlights a key distinction between physiological correction of vitamin D deficiency and pharmacological administration of active vitamin D metabolites. While correction of deficiency is clinically reasonable, high-dose calcitriol or vitamin D analogues may have complex effects on drug disposition and should be evaluated carefully in cardio-oncology settings.

Vitamin D has also been explored as part of drug-delivery strategies. Sabzichi et al. [331] developed vitamin D-loaded nanostructured lipid carriers to improve the effectiveness of vitamin D3 in breast cancer cells. Kutlehria et al. [332] designed cholecalciferol-PEG conjugate-based nanomicelles for DOX delivery in triple-negative breast cancer, suggesting that vitamin D-derived structures may improve anticancer delivery and potentially reduce systemic toxicity. Gao et al. [333] similarly developed hyaluronic acid–cholecalciferol conjugate-based nanomicelles for DOX delivery against MCF-7 breast cancer cells. These studies illustrate the broader translational relevance of vitamin D biology and cholecalciferol-based platforms in DOX therapy. Recent in vitro data also support the concept that vitamin D3 may have dual pharmacological potential as both a cardioprotective and anticancer agent [334]. Such findings are conceptually important because any nutritional intervention proposed for cardio-oncology must satisfy two requirements: protection of non-malignant cardiovascular tissue and preservation, or ideally enhancement, of anticancer efficacy. However, in vitro dual-action studies remain hypothesis-generating and require validation in animal models and clinical trials.

The available evidence suggests that hypovitaminosis D may contribute to increased biological vulnerability in cancer patients, including potential susceptibility to cardiotoxicity, but this relationship remains insufficiently proven clinically. The mechanistic rationale is strong: vitamin D signaling may attenuate DOX-induced OS, mitochondrial dysfunction, calcium dysregulation, endothelial injury, EndMT, inflammation, and fibrosis. In parallel, clinical studies in lymphoma indicate that low vitamin D status is associated with inferior treatment outcomes, supporting the broader relevance of vitamin D status in oncology. However, the current evidence does not yet establish low 25(OH)D as an independent predictor of anthracycline-related cardiac dysfunction. Therefore, baseline assessment and correction of vitamin D deficiency may be considered as part of supportive care in oncology patients, particularly in those receiving potentially cardiotoxic chemotherapy. Nevertheless, dedicated cardio-oncology trials are needed to determine whether correction of hypovitaminosis D reduces troponin release, preserves GLS, prevents decline in LVEF, limits myocardial fibrosis, or improves long-term cardiovascular outcomes. Such trials should also monitor anticancer efficacy, recurrence, survival, calcium metabolism, renal function, and possible pharmacokinetic interactions with DOX.

Overall, these findings are reassuring from the perspective of oncological safety, but they cannot be generalized across all tumor types, doses, vitamin D metabolites, or treatment schedules. Compared with vitamins C and E, vitamin D may be particularly relevant as an immunomodulatory and anti-inflammatory regulator rather than as a direct antioxidant. However, current evidence remains insufficient to recommend routine vitamin D supplementation specifically for prevention of DOX cardiotoxicity in all patients. Future studies should define the role of baseline vitamin D deficiency, optimal 25(OH)D target ranges, vitamin D form and dose, treatment timing, interaction with calcium metabolism, and effects on long-term cardiovascular and oncological outcomes.

### 8.6. Emerging Nutritional Interventions

In addition to polyphenols, omega-3 fatty acids, coenzyme Q10, selenium, and antioxidant vitamins, several emerging nutritional or nutrition-related interventions have been investigated for prevention of OS-induced cardiotoxicity. These include N-acetylcysteine, melatonin, dietary nitrate, and functional foods or food-derived bioactive preparations. Although these interventions differ substantially in chemical structure and biological targets, they share several mechanistic features relevant to AIC, including modulation of redox balance, mitochondrial function, nitric oxide signaling, inflammation, apoptosis, fibrosis, and cellular stress responses. Importantly, the evidence base remains heterogeneous. Most data are preclinical, while clinical evidence is limited and often underpowered. Therefore, these interventions should be discussed as promising but not yet established cardioprotective strategies.

#### 8.6.1. N-Acetylcysteine

N-acetylcysteine (NAC) is a thiol-containing compound and a precursor of intracellular cysteine and glutathione. Its cardioprotective rationale in DOX-induced cardiotoxicity is based on its ability to replenish glutathione, increase cellular sulfhydryl reserves, reduce oxidative and nitrosative stress, and modulate redox-sensitive inflammatory and apoptotic signaling. Since DOX causes mitochondrial ROS generation, lipid peroxidation, depletion of antioxidant defenses, and activation of cardiomyocyte death pathways, NAC has been investigated as a potential redox-modulating intervention.

Early experimental evidence showed that NAC could reduce DOX-induced cardiac injury in mice. Doroshow et al. [335] demonstrated that NAC pretreatment improved survival, reduced long-term mortality after repeated DOX exposure, attenuated loss of body and heart weight, and prevented ultrastructural evidence of DOX cardiomyopathy without altering DOX uptake or metabolism in the heart or liver. The protective effect was associated with increased cardiac non-protein sulfhydryl content, supporting the concept that myocardial thiol redox status is relevant to AIC [335]. Subsequent animal studies also reported biochemical and histological protection. Arica et al. showed that NAC reduced DOX-induced oxidative injury and preserved myocardial architecture in rats, supporting antioxidant and tissue-protective mechanisms [336]. In recent experimental work, Tola and Kılıç [337] evaluated NAC in a rat model of DOX-induced cardiotoxicity and showed that DOX increased MDA levels and decreased antioxidant enzyme activities, including SOD, catalase, and GPx. NAC-treated animals showed substantial histological protection, with attenuation of edema, inflammation, vacuolization, hemorrhage, necrosis, and myofibrillar disorganization. However, the authors noted that NAC did not produce a clearly significant biochemical improvement across all OS parameters, suggesting that histological protection may not fully correlate with conventional redox biomarkers [337]. This study supports NAC as a promising adjunctive agent but also reinforces the need for more detailed molecular and functional endpoints before NAC can be considered a clinically established cardioprotective strategy. The relevance of thiol redox biology is further supported by studies on metallothioneins and NAC. Fu et al. [338] showed that cardiomyocytes from metallothionein-I/II null mice were more sensitive to DOX-induced cytotoxicity, LDH leakage, apoptosis, and ROS generation than wild-type cardiomyocytes. NAC and glutathione significantly rescued wild-type cardiomyocytes from DOX-induced cell death and ROS generation but did not provide the same protection in metallothionein-deficient cardiomyocytes [338]. This finding is important because it suggests that the efficacy of thiol-based interventions depends on intact endogenous metal- and redox-buffering systems. Therefore, NAC should not be viewed simply as a universal ROS scavenger but as part of a broader thiol–glutathione–metallothionein antioxidant network.

However, the effects of NAC are not uniformly protective. Shi et al. [339] showed that NAC amide decreased OS in DOX-treated H9c2 cardiomyocytes but had minimal effect on DOX-induced cell death. This finding is important because it illustrates a recurring theme in anthracycline cardioprotection: reducing ROS markers alone may not be sufficient to prevent cell death when other mechanisms, such as mitochondrial dysfunction, TOP2β-mediated DNA damage, calcium dysregulation, ferroptosis, and apoptotic signaling, are already activated. In a murine model of combined DOX and trastuzumab cardiotoxicity, NAC amide attenuated cardiac dysfunction by reducing OS and apoptosis, suggesting that NAC derivatives with improved bioavailability may have greater translational potential than conventional NAC [340]. Recent studies by Tiwari and colleagues broaden the mechanistic framework beyond mitochondrial ROS by implicating nuclear lamina disruption in DOX cardiotoxicity. In one study, DOX induced phosphorylation of lamin (Lmn) A/C at serine 22, reduced total *Lmna* expression, altered nuclear shape, caused nuclear membrane thinning and perforation, and promoted cell death in H9c2 cardiomyoblasts and rat hearts. Overexpression of *Lmna* protected cells from DOX-induced injury, while treatment with the CDK1 inhibitor purvalanol A or NAC improved LmnA/C levels [341]. This study is particularly relevant because it suggests that redox stress may destabilize nuclear architecture and that antioxidant strategies such as NAC may exert protection partly by preserving nuclear membrane integrity. The follow-up study extended this mechanism by showing that DOX-induced LmnA/C phosphorylation enhances DNMT1 expression and DNA methylation, leading to reduced expression of cardioprotective genes such as *Gata-4* and *Bcl-xL* and activation of apoptotic cell death [342]. This provides a mechanistic bridge between OS, nuclear structural injury, epigenetic remodeling, and cardiomyocyte death. These studies are useful because they show that nutritional cardioprotection should focus not only on neutralizing ROS but also on preventing downstream nuclear, transcriptional, and epigenetic consequences of redox injury.

Clinical evidence for NAC in AIC is limited and not clearly positive. The EPOCH trial evaluated short-term high-dose NAC as a strategy to prevent anthracycline-induced cardiomyopathy in a prospective randomized study. The trial did not demonstrate significant prevention of anthracycline-induced cardiomyopathy, with comparable changes in left ventricular function, cardiac enzymes, and clinical event rates between NAC and control groups [343]. Thus, although NAC is mechanistically plausible and supported by several preclinical studies, current clinical evidence does not justify routine use for anthracycline cardioprotection. Future studies would need to define optimal timing, dose, formulation, patient selection, and whether NAC derivatives with better cellular penetration provide superior protection.

#### 8.6.2. Melatonin

Melatonin is an indoleamine produced mainly by the pineal gland, but it is also synthesized in extrapineal tissues, including mitochondria. Beyond its role in circadian regulation, melatonin has potent antioxidant, anti-inflammatory, mitochondria-protective, anti-apoptotic, and anti-ferroptotic properties. These characteristics are highly relevant to DOX cardiotoxicity, where mitochondrial ROS generation, loss of mitochondrial membrane potential, impaired mitochondrial biogenesis, lipid peroxidation, calcium dysregulation, and cardiomyocyte death are central mechanisms.

A systematic review concluded that co-administration of melatonin ameliorates DOX-induced cardiotoxicity across experimental models [344]. A key mechanistic axis involves AMPK-dependent regulation of mitochondrial homeostasis. Melatonin-mediated activation of AMPK/PGC-1α has been shown to attenuate acute DOX cardiotoxicity by reducing mitochondrial oxidative damage and apoptosis. However, this pathway is context-dependent. A separate study demonstrated that DOX promoted AMPKα2 mitochondrial translocation and AMPKα2-dependent mitochondrial injury, whereas melatonin suppressed this maladaptive response [345,346]. These apparently divergent findings suggest that the cardioprotective effect of melatonin depends not simply on “AMPK activation” but on the timing, isoform specificity, and subcellular localization of AMPK signaling. Importantly, melatonin may also have favorable oncological interactions. Tran et al. showed that melatonin and DOX synergistically induced apoptosis in human breast cancer cells through autophagy-dependent downregulation of AMPKα1 transcription, a survival-related signaling component in cancer cells [347]. In the context of cardiotoxicity, modulation of AMPKα1 signaling may represent a potential adjunctive strategy to increase tumor cell sensitivity to anticancer drugs while simultaneously limiting their adverse effects on non-malignant cardiac tissue.

The Hippo/YAP pathway represents another mechanistic node through which melatonin may preserve cardiomyocyte viability. Sun et al. [348] further demonstrated that DOX reduces YAP expression, thereby promoting OS, mitochondrial dysfunction, and apoptosis. Melatonin-mediated preservation of YAP signaling has been associated with reduced cardiomyocyte apoptosis and improved resistance to DOX-induced injury. A related study further connected YAP preservation with attenuation of mitochondrial oxidative damage and ferroptosis, indicating that YAP may integrate survival signaling, mitochondrial homeostasis, and iron-dependent lipid peroxidation, linking melatonin to iron-dependent lipid peroxidation and mitochondrial quality control [348]. These findings suggest that melatonin is not simply a direct free radical scavenger, but a pleiotropic regulator of mitochondrial adaptation, redox signaling, apoptosis, autophagy, and ferroptosis. Recent work has further expanded this mechanistic framework. Zhang et al. [26] showed that melatonin alleviated DOX-induced cardiotoxicity through activation of the Sirt1/Nrf2 pathway pathways involving mitochondrial function and inflammatory-redox regulation. Activation of the SIRT1/Nrf2 axis has been implicated in the attenuation of OS, pyroptosis, and apoptosis in DOX-exposed myocardium. This mechanism is conceptually important because it links melatonin not only to direct antioxidant activity but also to endogenous cytoprotective transcriptional programs. By promoting Nrf2-dependent antioxidant defense and SIRT1-associated mitochondrial and anti-inflammatory responses, melatonin may shift cardiomyocytes away from redox collapse, inflammatory cell death, and mitochondrial failure. Indeed, melatonin has been shown to preserve mitochondrial membrane potential, reduce mitochondrial ROS production, improve mitochondrial dynamics, inhibit apoptosis, suppress inflammation, and potentially modulate mitophagy and ferroptosis in DOX-exposed myocardium [349].

Autophagy represents another mechanistically important but complex target. DOX may either stimulate maladaptive autophagy or impair autophagic flux and mitophagy, depending on the experimental model and phase of injury. Therefore, melatonin should not be simplistically described as either an autophagy inducer or inhibitor. Rather, current evidence suggests that melatonin may restore mitochondrial quality control and proteostasis-related balance under DOX-induced stress. Recent work also indicates that melatonin can improve cardiac metabolic reprogramming in DOX-induced heart failure and may act synergistically with non-pharmacological interventions such as therapeutic exercise, further supporting its role as a mitochondrial and metabolic modulator [350,351,352].

Ferroptosis and ferritinophagy have emerged as particularly relevant mechanisms in AIC. DOX promotes iron dysregulation, lipid peroxidation, and ferroptotic cardiomyocyte death, while melatonin appears to counteract this injury by preserving GPx4 activity, reducing pro-ferroptotic lipid remodeling and limiting mitochondrial oxidative damage. The study evaluating combined melatonin and deferoxamine is especially relevant because it links melatonin to iron-handling pathways and suggests that combined targeting of OS and iron-dependent injury may produce synergistic cardioprotection. Nevertheless, this concept remains preclinical and requires careful translational validation before being extrapolated to patients receiving anthracyclines [348,353].

The protective effects of melatonin have also been examined in combination models involving other cardiotoxic cancer therapies. In DOX- and trastuzumab-induced cardiotoxicity, melatonin reduced oxidative injury and improved biochemical and functional indices of myocardial damage, suggesting potential relevance in breast cancer regimens where anthracycline exposure and HER2-targeted therapy may produce additive or sequential cardiac risk. In other animal studies, melatonin showed cardioprotective effects comparable or complementary to adrenomedullin and metformin, with improvement in OS, inflammation, mitochondrial dynamics, apoptosis, and left ventricular function [354,355,356].

The most clinically relevant direct evidence comes from a triple-blind, placebo-controlled randomized trial in non-metastatic breast cancer patients receiving DOX plus cyclophosphamide. In this study, 63 patients were randomized to melatonin or placebo; melatonin was administered at 10 mg at bedtime from the first chemotherapy cycle until one week after the last cycle. The investigators assessed echocardiographic parameters and cardiac biomarkers, including LVEF, GLS, cardiac troponin I, and CK-MB. Melatonin did not produce a statistically significant improvement in LVEF, which is unsurprising given the relatively short follow-up and the limited sensitivity of LVEF for early myocardial injury. However, GLS was significantly better preserved in the melatonin group, and cardiac troponin I levels were significantly lower compared with placebo. The incidence of mild asymptomatic cancer therapy-related cardiac dysfunction was also markedly lower in the melatonin group [357]. Although promising, this evidence remains preliminary and should be interpreted cautiously until larger randomized trials confirm efficacy, optimal dose, timing, and long-term cardiac outcomes. An older randomized clinical study by Lissoni and colleagues [358] evaluated melatonin in patients with metastatic solid tumors and poor clinical status receiving chemotherapy. In that study, 250 patients with metastatic lung, breast, gastrointestinal, or head and neck cancers were randomized to chemotherapy alone or chemotherapy plus oral melatonin at 20 mg/day. The authors reported reduced chemotherapy-related toxicity and improved efficacy-related outcomes, including survival signals, in the melatonin-treated group. This study is notable because it suggests that melatonin may reduce systemic chemotherapy toxicity without impairing anticancer activity. However, its relevance to contemporary cardio-oncology is limited by the heterogeneity of tumor types, chemotherapy regimens, and toxicity endpoints, as well as the absence of modern cardiac imaging, troponin-based surveillance, standardized cancer therapy-related cardiac dysfunction definitions, and long-term cardiovascular follow-up [358].

#### 8.6.3. Functional Foods

Functional foods and food-derived bioactive preparations represent a broad and heterogeneous category that includes polyphenol-rich fruits, berries, vegetables, herbs, spices, fermented foods, marine polysaccharides, garlic preparations, olive-derived compounds, and other edible plant extracts. Their potential cardioprotective effects are generally attributed to combinations of antioxidant, anti-inflammatory, mitochondrial, anti-apoptotic, anti-fibrotic, and endothelial-protective mechanisms. In contrast to isolated nutraceuticals, functional foods contain complex mixtures of bioactive compounds that may act synergistically, but this complexity also makes standardization, dose definition, and clinical translation difficult.

A comprehensive review of edible plant extracts and foodborne phytochemicals in DOX cardiotoxicity concluded that appropriately designed diets or supplements based on antioxidant-rich edible plants may reduce chemotherapy-associated cardiac injury, although the evidence remains mostly preclinical [359]. Earlier reviews also emphasized that phytochemicals and herbal products can counteract DOX cardiotoxicity through modulation of OS; inflammation; mitochondrial dysfunction; apoptosis; calcium handling; and signaling pathways such as Nrf2, NF-κB, AMPK, SIRT1, and MAPK [360,361].

Several functional food-derived preparations have shown cardioprotective activity in experimental DOX models. Aged garlic extract is notable because it has been evaluated as a whole-food-derived intervention rather than a single isolated molecule. Experimental studies showed that aged garlic extract attenuated DOX-induced ECG abnormalities, oxidative injury, and cardiomyocyte damage [362]. Later work suggested that aged garlic extract protected cardiac myocytes against p53-mediated apoptotic signaling and did not interfere with DOX antitumor activity; in tumor-bearing mice, it increased DOX uptake into tumor cells and improved survival [363]. In addition, aged garlic extract protected against DOX-induced free radical production and cardiotoxicity in rats, supporting the relevance of organosulfur-rich foods and thiol-related redox modulation [364]. Although these findings are intriguing, aged garlic extract has not been validated in modern clinical trials using cardiac biomarkers, GLS, LVEF, and long-term heart failure outcomes. Cranberry extract attenuated DOX-induced cardiotoxicity in rats, likely through antioxidant and anti-inflammatory mechanisms linked to its polyphenol content [365]. Fucoidan, a sulfated polysaccharide derived from brown seaweed, protected against DOX-induced cardiotoxicity by reducing OS and preventing mitochondrial ferroptosis of cardiomyocytes [366]. These examples illustrate the diversity of functional food candidates, ranging from sulfur-containing foods to berry polyphenols and marine polysaccharides. Green tea catechins, particularly epigallocatechin-3-gallate (EGCG), represent another relevant functional-food class. EGCG has been shown to attenuate DOX cardiotoxicity by suppressing OS, inflammation, calcium overload, and apoptotic signaling. In experimental models, EGCG improved ECG abnormalities; reduced CK-MB and LDH leakage; restored glutathione and antioxidant enzyme activity; reduced lipid peroxidation; modulated ErbB2 pro-survival signaling; and suppressed NF-κB, p53, calpain, caspase-3, and caspase-12 activation [367]. In tumor-bearing mice, EGCG reduced DOX-induced ROS generation, preserved mitochondrial membrane potential, increased MnSOD expression, reduced myocardial calcium overload, and did not compromise antitumor activity; indeed, the combination enhanced tumor growth inhibition compared with DOX alone [368]. These results are promising, but clinical validation is lacking and high-dose green tea extracts require caution because of potential hepatotoxicity and pharmacokinetic interactions. Grape-derived polyphenols, including grape seed proanthocyanidins, catechins, and procyanidins, have also shown consistent preclinical cardioprotection. These compounds act as potent radical scavengers, preserve mitochondrial integrity, reduce DNA fragmentation, suppress caspase activation, and improve antioxidant enzyme activity. Importantly, grape seed proanthocyanidins protected cardiomyocytes from DOX-induced oxidative injury without reducing the antiproliferative effect of DOX in MCF-7 breast cancer cells [369]. Experimental animal studies also showed that grape polyphenol concentrates and grape seed extracts improved cardiac biomarkers, OS parameters, ECG abnormalities, and myocardial histology following DOX exposure [370,371]. These data support grape polyphenols as promising functional-food candidates, although their heterogeneous composition and variable bioavailability complicate clinical translation. Lycopene, a carotenoid abundant in tomatoes and tomato-based foods, has shown mixed effects. Early studies reported that tomato extract and lycopene attenuated DOX-induced OS and cardiomyocyte injury [372,373]. However, one echocardiographic rat study found that lycopene did not prevent DOX-induced left ventricular systolic dysfunction, although it reduced morphological myocyte injury [374]. More recent work using lycopene-loaded liposomes showed enhanced antitumor efficacy in vivo in B16 melanoma-bearing mice and reduced cardiotoxicity when combined with DOX, indicating that liposomal formulation improved bioavailability and biological efficacy [375].

Sulforaphane, an isothiocyanate derived from cruciferous vegetables, is mechanistically important because it targets the Nrf2/ARE pathway rather than acting only as a direct antioxidant. Sulforaphane protected against DOX-induced OS and H9c2 rat myoblast death through the induction of HO-1 and broader Nrf2-dependent antioxidant defense [376]. In vivo evidence further supports the cardioprotective role of sulforaphane in anthracycline-related myocardial injury. In a DOX-induced chronic heart failure model, sulforaphane prevented the development of cardiac dysfunction, and this effect was associated with the upregulation of Nrf2 expression and transcriptional activity, supporting Nrf2-dependent reinforcement of endogenous antioxidant defense as a key mechanism of protection [377,378].

Citrus flavanones such as naringenin and naringin also demonstrate cardioprotective activity in DOX models. Naringenin reduced troponin T, LDH, MDA, inflammatory cytokines, and histological cardiac injury, while restoring SOD, catalase, and GPx activity [379]. Naringenin-7-O-glucoside activated ERK1/2–Nrf2 signaling and induced endogenous antioxidant enzymes such as NQO1, GCLC, and GCLM in cardiomyocytes [380]. In addition, naringin protected H9c2 cells by suppressing DOX-induced p38 MAPK activation and OS [381]. These results support a mechanistic role for citrus flavonoids in strengthening endogenous redox defense, but clinical data are absent.

However, several limitations must be emphasized. Many functional food studies use extracts at doses that may not be achievable through normal diet. Also, extract composition varies according to plant species, cultivar, processing, storage, extraction method, and batch standardization. Some functional foods or herbal preparations may alter drug metabolism, platelet function, bleeding risk, or chemotherapy pharmacokinetics. Finally, few studies simultaneously assess cardioprotection and antitumor efficacy. Therefore, functional foods should be discussed as a source of promising bioactive compounds and dietary patterns rather than as established therapeutic agents. Future studies should prioritize standardized preparations, clinically relevant dosing, pharmacokinetic and pharmacodynamic profiling, biomarker-guided patient selection, and simultaneous assessment of cardiac safety and oncological efficacy.

### 8.7. Whole-Dietary Strategies and Mediterranean-Style Dietary Patterns in Pediatric Cancer Survivorship

While individual nutrients and nutraceuticals provide important mechanistic insights into redox regulation and cardioprotection, their translation into long-term survivorship care presents several challenges, including uncertain optimal dosing, bioavailability, potential nutrient–drug interactions, and the oncological safety of sustained high-dose supplementation. Whole-dietary strategies provide a complementary and potentially more readily implementable approach because they modify multiple cardiovascular risk pathways simultaneously rather than targeting a single molecular mechanism. In pediatric cancer survivors, this distinction is particularly relevant because cardiovascular prevention may need to continue for decades after completion of anticancer therapy.

Among whole-dietary approaches, the Mediterranean dietary pattern is of particular interest because it emphasizes vegetables, fruits, legumes, whole grains, nuts, fish, and unsaturated fats, while limiting highly processed foods and excessive intake of saturated fat and refined carbohydrates. Rather than providing pharmacological doses of individual antioxidants, such a dietary pattern delivers a complex matrix of polyphenols, carotenoids, vitamins, minerals, omega-3 fatty acids, fiber, and other bioactive compounds that may collectively influence oxidative stress, inflammation, endothelial function, lipid metabolism, insulin sensitivity, body composition, and gut microbial metabolism. However, its potential relevance to cancer therapy-related cardiotoxicity should presently be considered primarily in terms of long-term cardiovascular risk modification rather than direct prevention of myocardial injury.

This distinction is supported by observations in childhood leukemia survivors. In the PETALE cohort of 241 survivors of childhood ALL, Bérard et al. [382] demonstrated generally poor dietary quality, with particularly low adherence to the Mediterranean diet and substantial consumption of ultra-processed foods. A more pro-inflammatory dietary pattern was associated with lower HDL cholesterol and greater insulin resistance, whereas higher consumption of ultra-processed foods was associated with low HDL cholesterol and elevated triglycerides. Diet quality was also associated with circulating inflammatory and metabolic biomarkers, including TNF-α, IL-6, and adiponectin. These findings provide clinically relevant evidence that dietary patterns may contribute to the cardiometabolic phenotype of childhood leukemia survivors, although the observational design does not establish causality [382]. Similar concerns have been observed across broader childhood cancer survivor populations. In the Cardiac Risk Factors in Childhood Cancer Survivors Study, overall diet quality was only moderate, and better Healthy Eating Index scores were associated with lower percentage body fat [383]. More recently, early childhood cancer survivors have also been shown to have a high prevalence of suboptimal dietary quality together with multiple cardiovascular risk factors, emphasizing the importance of addressing nutrition as part of survivorship care [384]. Importantly, whole-dietary interventions appear feasible in survivorship settings. In the multicenter CARE for CAYA program, structured dietary counseling in adolescent and young adult cancer survivors improved Mediterranean dietary adherence and overall dietary quality over 52 weeks, although the intensified intervention did not produce a significant between-group advantage in overall diet-quality improvement compared with basic counseling. These findings support the feasibility of diet-based survivorship interventions but also highlight the need for more intensive and specifically targeted trials with cardiovascular endpoints [385].

Accordingly, Mediterranean-style and other high-quality dietary patterns should not currently be presented as substitutes for established cardio-oncology surveillance or pharmacological cardioprotection. Rather, they represent a potentially sustainable component of life-course cardiovascular risk reduction, particularly through prevention or modification of obesity, dyslipidemia, insulin resistance, hypertension, systemic inflammation, and other cardiometabolic factors that may amplify treatment-related myocardial vulnerability. Future prospective studies should determine whether sustained improvement in dietary quality can modify long-term cardiovascular events in survivors exposed to anthracyclines and/or radiotherapy.

### 8.8. The Antioxidant Paradox and Oncological Safety of Nutritional Cardioprotection

A major translational challenge in nutritional cardioprotection is the so-called antioxidant paradox. Interventions that reduce oxidative injury in cardiomyocytes may theoretically also interfere with ROS-dependent mechanisms contributing to antineoplastic activity [386]. This concern is biologically plausible because modulation of intracellular antioxidant capacity can alter the susceptibility of malignant cells to redox-active anticancer agents. Experimental studies have shown, for example, that increasing glutathione peroxidase activity can reduce DOX-induced oxidative injury and cytotoxicity in tumor cells, whereas lowering antioxidant capacity can enhance DOX-mediated apoptosis and antineoplastic activity [279,387]. However, the biological consequences of redox-active nutritional interventions cannot be reduced to a simple antioxidant-versus-prooxidant dichotomy. Rather, their effects depend on the specific compound, dose and achieved tissue concentration, formulation, route of administration, tumor phenotype, intracellular and extracellular redox environment, iron availability, and the mechanism of action of the concomitant anticancer therapy.

The contrasting findings reported for selenium and vitamin C illustrate this complexity particularly well. Selenium-dependent enhancement of antioxidant defenses may protect normal tissues from oxidative injury; however, Gencheva et al. recently demonstrated that selenium supplementation increased selenoprotein-dependent antioxidant capacity and protected glioblastoma cells from the H_2_O_2_- and iron-dependent cytotoxicity induced by the prooxidant ascorbate/menadione combination [280]. Similarly, modulation of selenium-dependent GPx activity has been shown to influence DOX-induced ROS generation, apoptosis, and tumor-cell cytotoxicity, further indicating that selenium status may modify the response of malignant cells to redox-active treatment [279]. These observations support the possibility that enhancement of antioxidant capacity may be disadvantageous when the therapeutic strategy itself depends substantially on oxidative stress.

Conversely, vitamin C exhibits strongly concentration- and route-dependent redox behavior. At physiological concentrations achieved through oral intake, ascorbate predominantly functions as a reducing antioxidant, whereas pharmacological concentrations achievable by parenteral administration can promote extracellular H_2_O_2_ generation and exert prooxidant cytotoxicity against susceptible malignant cells. Chen et al. demonstrated that pharmacological ascorbate selectively generated H_2_O_2_ and induced cancer-cell death, while sparing several non-malignant cell types; subsequent in vivo work confirmed preferential generation of ascorbate radical and H_2_O_2_ in extracellular tissue fluid following parenteral administration [388,389]. Additional studies have confirmed that pharmacological ascorbate can sustain H_2_O_2_-dependent oxidative stress preferentially in tumor cells [390]. In the specific context of anthracyclines, Kurbacher et al. reported increased antineoplastic activity of DOX in the presence of ascorbic acid, while Bober et al. showed that vitamin C potentiated the antiproliferative effect of DOX in MCF-7 breast cancer cells [286,287]. Perveen et al. likewise demonstrated enhanced anthracycline-DNA interactions and greater inhibition of non-small-cell lung cancer cell lines in the presence of ascorbic acid [288]. Therefore, vitamin C cannot be considered uniformly protective against oxidative cytotoxicity; depending on concentration and biological context, it may function either as an antioxidant or as a pharmacological prooxidant.

These apparently divergent observations emphasize that cardioprotection and tumor protection are not necessarily equivalent consequences of redox modulation. Malignant and non-malignant cells differ in basal oxidative stress, mitochondrial metabolism, iron handling, antioxidant capacity, and vulnerability to oxidative perturbation, creating the possibility of differential tissue responses to the same intervention. Experimental models provide direct support for this context dependence: pharmacological ascorbate can generate oxidative stress preferentially in malignant cells, whereas supplementation with conventional antioxidants such as N-acetylcysteine or vitamin E has accelerated tumor progression in selected genetically engineered mouse models of lung cancer by suppressing ROS-dependent DNA damage and p53 signaling [391]. These findings should not be extrapolated indiscriminately across tumor types or antioxidant compounds, but they demonstrate why the oncological consequences of antioxidant supplementation cannot be predicted solely from its cardioprotective redox effects.

Clinical evidence also supports a cautious and individualized approach, although it remains insufficient to establish causality. In the prospective ancillary study of the SWOG S0221 breast cancer trial, concurrent use of antioxidant supplements, including vitamins A, C, and E; carotenoids; and CoQ10, before and during chemotherapy showed a non-significant trend toward a higher risk of recurrence and mortality, leading the investigators to recommend caution regarding supplement use during chemotherapy [392]. Similarly, an observational cohort of postmenopausal women with breast cancer reported that antioxidant supplementation concurrent with chemotherapy or radiotherapy was associated with poorer recurrence-free survival and higher total mortality [393]. Importantly, these observational associations do not establish that antioxidant supplementation caused the adverse outcomes, but they underscore the need for prospective trials specifically designed to assess both cardiovascular and oncological endpoints.

Accordingly, current evidence is insufficient to exclude clinically relevant interactions between nutritional or nutraceutical cardioprotective strategies and the antitumor efficacy of concurrent cancer therapy. This consideration is particularly important for high-dose supplementation during active chemotherapy or radiotherapy. Future cardio-oncology studies should therefore evaluate cardiovascular efficacy and oncological safety simultaneously, including tumor response, treatment completion and dose intensity, recurrence, progression-free survival, and long-term oncological outcomes. Until such evidence becomes available, nutritional interventions should be regarded as individualized complementary strategies integrated with oncological and cardio-oncological care rather than as universally safe approaches based solely on their antioxidant properties.

When the available evidence is considered specifically from a pediatric cardio-oncology perspective, an important distinction emerges between biological plausibility and direct clinical validation. Among the nutritional strategies discussed in this review, omega-3 PUFAs currently have the strongest contemporary direct evidence in children with ALL receiving DOX, with randomized data demonstrating favorable effects on redox status, circulating markers of myocardial injury, and strain-based indices of left ventricular function. CoQ10 has also been evaluated directly in children receiving anthracyclines, but the available controlled study was small and predates contemporary cardio-oncology endpoints. Selenium also has limited direct pediatric clinical evidence, although this is based on a small study and does not constitute robust contemporary evidence of cardioprotective efficacy. In contrast, evidence supporting vitamins C and E, microbiota-directed interventions, one-carbon nutrients, and NAD^+^-restoring strategies remains predominantly derived from adult studies or preclinical models. Therefore, these approaches should not currently be considered equivalent in terms of clinical readiness. This evidence hierarchy is particularly important in pediatric oncology, where developmental physiology, nutritional requirements, treatment protocols, cumulative anthracycline exposure, and decades of subsequent survivorship preclude simple extrapolation from adult populations.

## 9. Nutrition-Based Epigenetic Cardioprotective Interventions

Environmental exposures, psychosocial stressors, and nutrition are all potentially important influences that may impact health outcomes directly or via interactions with the genome or epigenome over generations [394]. DOX-induced cardiotoxicity may occur due to multiple interconnected mechanisms, such as OS, mitochondrial dysfunction, lipid peroxidation, and DNA damage, further aggravated by the vulnerability of cardiomyocytes due to their high mitochondrial content and limited antioxidant capacity [395]. Nutrition, being one of the most powerful mechanisms for influencing gene expression, can reverse or change epigenetic phenomena, such as DNA methylation and histone modifications, thereby modifying the mechanisms involved in DOX-induced cardiac toxicity [21].

DNA methylation is a central epigenetic mechanism involved in the interaction between nutritional exposures and gene regulation [396]. Aberrant DNA methylation patterns induced in DOX-treated cardiomyocytes are the subject of research regarding the nutritional intervention in the prevention and/or reversal of DOX-induced cardiotoxicity. Human methylome is significantly shaped by the nutrients involved in one-carbon metabolism, such as folate, vitamin B12, vitamin B6, choline, betaine, and methionine, that supply the methyl groups required to produce SAM, the universal methyl donor for DNA, RNA, and histone methylation reactions [396].

Polyphenols, such as epigallocatechin gallate, curcumin, resveratrol, and genistein, as well as isothiocyanates, short-chain fatty acids (SCFAs), and carotenoids have been shown to influence DNA methylation. Polyphenol intake has been associated with minimizing OS and inflammation through the epigenetic reversal of hypermethylated NRF2 states, thus restoring the expression of NRF2, as a transcription factor crucial in regulating cellular homeostasis and apoptosis [397]. Epicatechin phase II metabolites were demonstrated to induce modifications in DNA methylation patterns in inflammatory-stimulated HUVEC cells, likely through interactions with DNMT1 and DNMT3A, further underscoring their epigenetic influence [398].

Polyphenols can affect DNA methylation through reduction, the modification of the expression of TFs and also the silencing of chromatin, the inhibition of DNA methyltransferases, and the activation of HDAC III (Sirtuins). Activated Sirtuins effectively attenuate DOX-induced myocardial injury by suppressing inflammation and oxidative damage [399,400]. Sirtuin-activation strategies as a potential intervention for DOX-induced cardiotoxicity are numerous, and one of the examples is resveratrol, which increases SIRT1 expression to attenuate DOX-induced cardiac dysfunction and DNA damage. These effects are associated with modifications of epigenetic PTMs, namely the reduced acetylation of lysine residue at position 5 of the H2AX histone tail (H2AX is the master DNA damage repair histone protein), as well as preserved levels of phosphorylation of H2AX on serine 139 [401].

In mouse models of DOX-induced cardiotoxicity, mitochondrial biogenesis and mitophagy were enhanced by ellagic acid, a bioactive polyphenolic compound naturally occurring as a secondary metabolite in many plants (such as pomegranate, persimmon, raspberry, and black raspberry). The molecular mechanisms of alleviated DOX-induced cardiotoxicity by ellagic acid comprise the activation of SIRT6 by deacetylating and inhibiting SGK1 (serum/glucocorticoid regulated kinase 1). Interestingly, in the same in vivo murine model, ellagic acid induced SIRT6 overexpression and potentiated the antitumor efficacy of DOX [402].

The impact of diet on miRNA regulation and its implications for health is further expanded by studies suggesting that miRNAs contained in foods can also be absorbed during the digestive process and consequently interact with host gene expression [403]. Phenolic compounds, such as ferulic acid, abundantly present in grains, fruits, vegetables, and herbs, attenuate ROS-induced H9c2 cell apoptosis, promoting miR-499-5p expression and inhibiting p21 expression [404].

An important pathway that promotes mitochondrial production of ROS is the p66Shc pathway, overexpressed during DOX treatment, leading to reduced antioxidant enzyme activity and exacerbating oxidative damage. p66Shc, a member of the Src collagen homologue A family, is an important redox-sensitive and pro-apoptotic adaptor protein, that responds to cell stress by translocating to mitochondria, where p66Shc oxidoreductase produces pro-apoptotic ROS. In DOX-treated rodents, the p66shc pathway mediates DOX-induced apoptosis of cardiomyocytes [405]. However, SIRT1 suppresses p66Shc expression, mitigating OS. Investigations of this important pathway revealed that molecules such as berberine (a plant alkaloid) and miR-124 have been suggested to attenuate DOX-related myocardial damage [406].

Multi-level regulation of cellular homeostasis exerted by miRNAs, mainly via the post-transcriptional control of gene networks, can be further modified by nutrition and natural compounds, such as resveratrol. Activation of SIRT1 can mitigate DOX-induced cardiotoxicity by preventing oxidative damage and apoptosis, where the resveratrol and the sirtuin–miRNA axis create a bidirectional, protective regulatory loop. The importance of the crosstalk between microRNAs and sirtuins in DOX-induced cardiotoxicity is further emphasized by data showing that resveratrol inhibits the miR-217/SIRT1 axis, ultimately restoring SIRT1 levels. In addition, resveratrol as a potent agonist of SIRT1, exerts cardioprotective effects through upregulation of cardioprotective miR-21 and SIRT1 deacetylase [406].

### 9.1. One-Carbon Metabolism, Folate, Methionine, and S-Adenosylmethionine

One-carbon metabolism provides an important biochemical interface between nutrition, redox homeostasis, mitochondrial function, and epigenetic regulation. Folate, vitamins B6 and B12, choline, betaine, and methionine participate in interconnected metabolic reactions that regulate homocysteine remethylation and the generation of SAM, the principal methyl donor for DNA, RNA, and histone methylation. Consequently, disturbances in one-carbon metabolism may influence both the availability of methyl groups required for epigenetic regulation and cellular antioxidant capacity through their interaction with methionine and transsulfuration pathways. This relationship is particularly relevant to DOX-induced cardiotoxicity, in which oxidative stress, mitochondrial injury, altered DNA methylation, and dysregulation of stress-response genes occur simultaneously.

Among one-carbon nutrients, folate has the most direct experimental evidence for cardioprotection against DOX. In a murine model of acute DOX-induced cardiomyopathy, folic acid pretreatment reduced mortality; preserved stroke volume; and attenuated cardiomyocyte atrophy, apoptosis, interstitial fibrosis, and mitochondrial dysfunction [407]. Mechanistically, folic acid reduced superoxide generation by improving endothelial nitric oxide synthase (eNOS) coupling, decreasing eNOS S-glutathionylation, preserving eNOS phosphorylation and nitric oxide availability, and maintaining endogenous antioxidant defense. These findings are relevant because uncoupled eNOS shifts from nitric oxide production toward superoxide generation and may thereby amplify oxidative and nitrosative stress during anthracycline exposure. Thus, folate-dependent cardioprotection may extend beyond its classical role as a methyl-group donor and include direct effects on redox signaling, endothelial function, and mitochondrial integrity.

The relationship between folate, methionine metabolism, and DOX cardiotoxicity is further supported by experimental evidence involving homocysteine. Dietary methionine-induced mild hyperhomocysteinemia aggravated DOX-induced cardiac dysfunction, myocardial atrophy, myofibrillar disorganization, fibrosis, and systemic and myocardial oxidative stress in mice [408]. Additional folic acid supplementation almost completely prevented the rise in circulating homocysteine and attenuated the associated deterioration in cardiac function and oxidative injury. These findings suggest that disturbances in one-carbon metabolism may modify susceptibility to anthracycline cardiotoxicity and raise the possibility that folate status and circulating homocysteine could represent relevant components of nutritional risk assessment. However, the protective effect observed in this model should not be interpreted as evidence that high-dose folic acid supplementation is beneficial in unselected patients because the intervention primarily corrected experimentally induced hyperhomocysteinemia.

Recent evidence also indicates that the relationship between methionine availability and cardiotoxicity is more complex than a simple linear association. Methionine deficiency was shown to exacerbate DOX-induced cardiac injury by increasing oxidative stress, impairing mitochondrial integrity, and disrupting autophagy and mitophagy, whereas appropriate methionine supplementation partially restored mitochondrial quality control and attenuated cardiotoxicity through general control nonderepressible 2 (GCN2)-dependent signaling [409]. When considered together with the detrimental effects of excessive methionine intake and hyperhomocysteinemia, these observations suggest that maintenance of methionine homeostasis, rather than indiscriminate supplementation or restriction, may be particularly important during anthracycline exposure. This concept is consistent with a precision-nutrition approach in which nutritional deficiencies and metabolic abnormalities are corrected according to individual biochemical status rather than by universal high-dose supplementation.

The most recent experimental data further broaden the mechanisms through which folic acid may influence DOX-related cardiac injury. In a 2026 rat study, folic acid attenuated DOX-induced deterioration of echocardiographic and biochemical cardiac parameters and reduced oxidative and inflammatory injury [410]. Folic acid decreased lipid peroxidation and inflammatory mediators, including IL-6, TNF-α, and NLRP3; preserved myocardial architecture; and reduced NOX4 expression. Interestingly, the intervention also affected SCFA profiles and was investigated in parallel with a postbiotic preparation, suggesting potential crosstalk between one-carbon metabolism, intestinal microbial metabolism, inflammatory signaling, and myocardial redox homeostasis. These observations provide an additional link between the nutritional epigenetic framework and the gut–heart axis discussed in this review.

SAM provides a further mechanistic link between nutrient availability and epigenetic regulation because the cellular SAM/S-adenosylhomocysteine ratio is a major determinant of methylation potential. Direct evidence for SAM supplementation in DOX-induced cardiotoxicity is limited and substantially older than that available for several other nutritional interventions. In a rat model, pretreatment with exogenous SAMe attenuated DOX-induced electrocardiographic and myocardial morphological abnormalities and reduced lethality [411]. The reported protection was attributed primarily to enhanced glutathione availability and antioxidant activity, rather than to experimentally demonstrated changes in DNA or histone methylation. Therefore, although SAM is biologically central to epigenetic regulation, current evidence does not establish that SAM supplementation prevents DOX cardiotoxicity through direct epigenetic reprogramming.

Overall, one-carbon metabolism represents a biologically plausible component of nutritional cardioprotection, linking methyl-donor availability with redox status, mitochondrial function, homocysteine metabolism, and potentially epigenetic regulation. However, an important distinction should be maintained between mechanistic plausibility and therapeutic evidence. Most data supporting folate, methionine, or SAM manipulation in DOX cardiotoxicity remain preclinical, and no adequately powered clinical trials have demonstrated that targeted modulation of one-carbon metabolism prevents anthracycline-related cardiac dysfunction. This limitation is particularly important in pediatric oncology, where folate and methionine requirements are influenced by growth, nutritional status, concurrent chemotherapy, and other metabolic demands. Accordingly, correction of documented deficiencies or metabolic abnormalities appears more defensible than empirical high-dose methyl-donor supplementation until prospective clinical studies establish efficacy and oncological safety.

### 9.2. NAD^+^ Precursors and Sirtuin-Dependent Metabolic–Epigenetic Cardioprotection

NAD^+^ serves as a key interface between cellular metabolism, redox homeostasis, mitochondrial function, and epigenetic regulation. In addition to its classical role as a redox coenzyme, NAD^+^ is an obligatory substrate for sirtuins, a family of NAD^+^-dependent deacetylases and deacylases that regulate mitochondrial biogenesis, antioxidant defenses, inflammatory signaling, chromatin structure, DNA repair, and cellular survival. Maintenance of intracellular NAD^+^ availability may therefore influence DOX cardiotoxicity at the intersection of cellular metabolism and epigenetic control.

Initial experimental evidence demonstrated a direct cardioprotective effect of the NAD^+^ precursor nicotinamide riboside (NR). In DOX-treated mice and cultured cardiomyocytes, NR supplementation increased intracellular and myocardial NAD^+^ levels, reduced cardiac injury and myocardial dysfunction, and attenuated oxidative stress [412]. Mechanistically, NR restored lysosomal acidification, prevented accumulation of dysfunctional autolysosomes, and improved autophagic flux. Importantly, inhibition of SIRT1 abolished these protective effects, supporting a mechanistic NAD^+^/SIRT1 axis rather than a nonspecific antioxidant action of NR. These findings are particularly relevant to DOX-induced cardiotoxicity because impairment of autophagic flux and accumulation of damaged mitochondria amplify ROS production, energetic failure, and regulated cardiomyocyte death.

Nicotinamide mononucleotide (NMN), another immediate NAD^+^ precursor, has subsequently shown protective effects in both acute and chronic experimental models. NMN administration increased tissue NAD^+^ concentrations and reduced DOX-induced cardiotoxicity, while also preserving physical function during prolonged follow-up [413]. Transcriptomic analyses indicated that NMN counteracted DOX-associated alterations in genes involved in mitochondrial function, oxidative stress, inflammation, apoptosis, ferroptosis, and p53-dependent signaling. Particularly noteworthy was its broad attenuation of the DOX-induced *p53* transcriptional response, including genes involved in cell-cycle arrest, cellular stress, DNA repair, senescence, and programmed cell death. These data suggest that restoration of NAD^+^ availability influences not only mitochondrial bioenergetics but also transcriptional stress programs involved in the progression from acute molecular injury to chronic myocardial dysfunction.

Subsequent studies have expanded this concept beyond acute myocardial injury. In a murine model of DOX-induced multiorgan toxicity, DOX markedly decreased NAD^+^ concentrations in the heart and other affected tissues, whereas NMN supplementation restored NAD^+^ availability; improved survival; and attenuated cardiac, hepatic, and pulmonary fibrosis [414]. The intervention reduced macrophage infiltration and suppressed profibrotic TGF-β/Smad signaling, indicating that NAD^+^ restoration may influence chronic inflammatory and fibrotic remodeling in addition to its effects on oxidative stress and mitochondrial function. These findings are relevant to long-term cancer survivorship, where myocardial fibrosis and progressive remodeling may continue after completion of anthracycline therapy.

Recent mechanistic studies provide further support for NAD^+^ homeostasis as an integral component of mitochondrial–lysosomal quality control. In 2025, histidine triad nucleotide binding protein-2 (HINT2) was identified as a regulator of mitochondrial complex I activity, the NAD^+^/NADH ratio, lysosomal function, and autophagic flux during DOX exposure [415]. Cardiac-specific HINT2 deficiency aggravated DOX-induced myocardial dysfunction and lysosomal impairment, whereas NMN restored lysosomal function in vitro. This work further integrates mitochondrial electron transport with NAD^+^ redox balance and autophagic clearance, suggesting that NAD^+^ depletion may represent a mechanistic bridge between impaired mitochondrial bioenergetics and defective cellular quality control.

The importance of NAD^+^ homeostasis has been strengthened further by a 2026 study demonstrating that DOX reprograms the kynurenine pathway, the principal pathway of de novo NAD^+^ synthesis [416]. DOX increased α-amino-β-carboxy-muconate-semialdehyde decarboxylase (ACMSD) while reducing quinolinate phosphoribosyltransferase (QPRT), thereby decreasing quinolinic acid availability and intracellular NAD^+^ levels. Genetic loss of indoleamine 2,3-dioxygenase 1 (IDO1) aggravated DOX-induced cardiac injury, whereas pharmacological targeting of the ACMSD/QPRT metabolic switch restored NAD^+^ homeostasis and attenuated cardiac oxidative injury. Importantly, this approach did not diminish DOX-induced cancer-cell death in the experimental setting, addressing, at least preclinically, the critical concern that cardioprotection might compromise anticancer efficacy. These findings extend the concept of nutritional NAD^+^ repletion beyond exogenous precursor supplementation and identify endogenous NAD^+^ biosynthesis as a potentially modifiable determinant of anthracycline cardiotoxicity.

Taken together, NR and NMN currently provide some of the strongest mechanistic evidence linking nutrient-sensitive metabolism to epigenetic and mitochondrial cardioprotection during DOX exposure. Nevertheless, this field remains preclinical. No randomized clinical trials have established that NAD^+^ precursor supplementation prevents anthracycline-induced cardiac dysfunction in cancer patients, and there are currently no direct data in children with leukemia. Moreover, NAD^+^ metabolism is involved in both normal and malignant cell survival, DNA repair, energy metabolism, and stress adaptation; consequently, effects on tumor biology, treatment efficacy, pharmacokinetics, dose, and timing require careful evaluation before NAD^+^-enhancing interventions can be translated into cardio-oncology practice. At present, NAD^+^ precursors should therefore be regarded as emerging metabolic–epigenetic cardioprotective candidates rather than clinically established nutritional interventions.

## 10. Functional Foods Targeting the Gut–Heart Axis in Doxorubicin Cardiotoxicity

Recent evidence substantially expands the concept of DOX-induced cardiotoxicity beyond a direct cardiomyocyte-centered toxicological model. Emerging evidence supports a broader gut–heart axis framework, in which DOX induces intestinal dysbiosis, disrupts epithelial barrier integrity, increases endotoxemia, modifies microbial metabolic outputs, and amplifies systemic inflammatory and OS responses that subsequently contribute to myocardial injury. This concept is particularly relevant for functional foods and dietary bioactive because many food-derived compounds have limited systemic bioavailability but profound effects on gut microbiota composition, intestinal barrier function, microbial metabolite production, and mucosal immune tone [417].

Experimental evidence from An et al. [418] first provided direct support for the causal involvement of microbiota in DOX-induced cardiac dysfunction in C57BL/6 mice. DOX produced dose-dependent cardiac injury; reduced LVEF and fractional shortening; increased myocardial fibrosis, inflammation, and OS; and was accompanied by intestinal damage characterized by reduced colorectal length, goblet cell loss, ulceration, lymphocytic infiltration, reduced tight-junction protein ZO-1, and elevated plasma endotoxin. 16S rRNA microbiota analysis revealed DOX-induced microbiota dysbiosis associated with decreased community richness compared with healthy control mice. Importantly, fecal microbiota transplantation (FMT) from healthy donors improved cardiac function and attenuated intestinal injury, suggesting that gut dysbiosis is not merely an epiphenomenon but may actively modulate DOX cardiotoxicity [418]. Consistent with this concept, Huang et al. [419] demonstrated that DOX alters both the composition and predicted functional capacity of the gut microbiome in mice. DOX-induced cardiotoxicity was associated with changes in bacterial taxa and microbial metabolic functions related to amino acid metabolism, glycan biosynthesis and metabolism, lipid metabolism, and secondary metabolite pathways. Notably, antibiotic-mediated depletion of gut microbiota alleviated DOX-induced myocardial injury and cardiomyocyte apoptosis, indicating that the dysbiosis can contribute to cardiotoxic signaling [419]. The relevance of gut microbiota is further supported by studies of DOX-induced heart failure. Fan et al. [420] described significant alterations in gut microbiota structure and function in rats with DOX-induced heart failure, reinforcing the idea that chronic anthracycline myocardial injury is accompanied by intestinal microbial remodeling. The results of the study suggest that microbiota changes are not restricted to acute toxicity models but may also accompany the transition toward chronic cardiac dysfunction [420].

Within this framework, functional foods and food-derived bioactives may exert cardioprotection through two complementary mechanisms: direct myocardial cytoprotection and indirect gut-mediated modulation of systemic toxicity. Glabridin, an isoflavone from licorice root, is a representative example. In mice, glabridin attenuated DOX-induced leakage of myocardial injury enzymes, reduced cardiomyocyte apoptosis, downregulated Bax, cleaved caspase-9 and cleaved caspase-3, and increased anti-apoptotic proteins such as Bcl-2 and HAX-1. Mechanistically, its effect was linked to gut microbiota remodeling, decreased lipopolysaccharide (LPS) levels, increased butyrate, and polarization of colonic macrophages away from a pro-inflammatory M1 phenotype toward an anti-inflammatory M2 profile. The involvement of LPS–NF-κB and butyrate–STAT6 signaling provides mechanistic bridge between intestinal dysbiosis, mucosal immunity, systemic inflammation, and myocardial apoptosis [421]. Yellow wine polyphenolic compounds provide another highly relevant functional-food model. Lin et al. [422] showed that these polyphenols protected against DOX-induced cardiotoxicity by modulating both the composition and metabolic function of gut microbiota. This study is particularly important because it links a complex dietary polyphenol mixture to improved cardiac and mitochondrial function, reduced inflammation, and microbiota-dependent metabolic remodeling. Together with earlier work showing that yellow wine polyphenols activate Nrf2 signaling, these findings suggest that polyphenol-rich functional foods may protect the myocardium through a dual mechanism: reinforcement of endogenous antioxidant defense and restoration of microbiota-derived metabolic homeostasis [422]. Zn(II)-curcumin supplementation provides a further example of a multi-target functional-food-derived intervention. In rats receiving repeated low-dose DOX, daily oral Zn(II)-curcumin attenuated gut dysbiosis; preserved the abundance of beneficial bacteria including *Clostridium* clusters, *Roseburia*, *Butyricicoccus*, and *Akkermansia*; maintained intestinal barrier integrity; and reduced fecal and plasma LPS levels. In parallel, Zn(II)-curcumin corrected DOX-induced zinc dyshomeostasis, preserved zinc transporter expression, improved cardiac function, and reduced cardiomyocyte apoptosis and myocardial injury [242]. The *Apocynum venetum* leaf extract studies are also relevant because this plant-derived intervention appears to combine classical anti-apoptotic cardioprotection with gut–metabolite modulation. Earlier work showed that *Apocynum venetum* leaf extract alleviated DOX cardiotoxicity through the AKT/Bcl-2 signaling pathway, thereby reducing cardiomyocyte apoptosis [423]. More recent evidence demonstrated that *Apocynum venetum* leaf extract regulated organic acid metabolism in gut microbiota and increased metabolites such as indole-3-propionic acid and acetic acid, both of which are biologically plausible mediators of antioxidant, anti-inflammatory, and mitochondrial protection [424].

FMT studies provide strong mechanistic support for microbiota-dependent cardioprotection. Zhou et al. [425] demonstrated that FMT protected mice against DOX-induced cardiac toxicity by regulating Nrf2-mediated mitochondrial dynamics. FMT altered gut microbiota and serum metabolites, with indole-3-propionic acid emerging as a key metabolite associated with improved cardiac function. Mechanistically, FMT and indole-3-propionic acid facilitated nuclear translocation of Nrf2, activated antioxidant gene expression, reduced ROS production, inhibited excessive mitochondrial fission, and preserved mitochondrial function. These findings are highly relevant because mitochondrial fragmentation, impaired mitochondrial fusion, OS, and ATP depletion are central events in AIC [425].

The study on phenylacetylglutamine further refines the metabolite-centered view of the gut–heart axis. Phenylacetylglutamine (PAGln), a gut microbiota-derived metabolite, attenuated DOX-induced myocardial injury and apoptosis in vitro and in vivo. Transcriptomic analysis implicated lipid metabolism, calcium-mediated signaling, store-operated calcium channel activity, and hypertrophic cardiomyopathy-related pathways. Candidate genes such as *Klb*, *Ece2*, *Nmnat2*, *Casq1*, *Pak1*, and *Apob* were reversed by PAGln treatment, with *Ece2* highlighted as a potential regulatory node related to endothelial dysfunction. This study is important because it suggests that microbiota-derived metabolites may modulate not only redox injury but also calcium handling, endothelial signaling, and metabolic remodeling during anthracycline exposure [426].

SCFA biology is further supported by the study of phenylalanine-butyramide, a butyrate derivative that protected against DOX-induced cardiotoxicity. Butyrate and related derivatives are mechanistically attractive because they may act through anti-inflammatory, mitochondrial, epigenetic, and barrier-protective mechanisms. Phenylalanine-butyramide attenuated DOX-induced cardiac dysfunction and oxidative/nitrosative stress, supporting the concept that SCFA-based or SCFA-mimetic interventions may represent a rational nutraceutical approach to anthracycline cardioprotection [427]. Ferroptosis has emerged as a major mechanism linking DOX cardiotoxicity with lipid peroxidation, mitochondrial damage, iron dysregulation, and glutathione-dependent antioxidant failure. Emodin, a naturally occurring anthraquinone found in several medicinal plants, ameliorated DOX-induced cardiotoxicity by inhibiting ferroptosis through remodeling of gut microbiota composition. This study is particularly important because it connects two rapidly developing fields: ferroptosis-targeted cardioprotection and microbiota-mediated modulation of chemotherapy toxicity. By reducing myocardial fibrosis, cardiomyocyte hypertrophy, and myocardial disorganization, emodin supports the hypothesis that gut microbiota remodeling may influence ferroptotic susceptibility in the myocardium [428].

Inflammasome signaling represents another critical mechanistic link between intestinal dysbiosis and myocardial injury. Yin et al. [62] showed that DOX activated the NLRP3 inflammasome, increased inflammatory signaling, and upregulated autophagy-associated LC3I/II proteins. NLRP3 knockout attenuated DOX-induced cardiac damage, modulated the blood immune microenvironment, and altered gut microbiota abundance. The study also suggested a relationship between NLRP3 signaling and *Akkermansia muciniphila* abundance. These findings indicate that NLRP3 may function as a nodal mediator connecting DOX-induced inflammation, gut microbiota remodeling, immune activation, and cardiac injury [62]. This is highly relevant for functional-food research because many dietary bioactive compounds, including polyphenols, flavonoids, SCFAs, and Nrf2 activators, can modulate NLRP3 activity either directly or indirectly through reduced OS, improved mitochondrial quality control, restoration of gut barrier function, and decreased LPS translocation. Therefore, the NLRP3–gut–heart axis may represent one of the most promising mechanistic frameworks for interpreting cardioprotection by microbiota-active functional foods.

Taken together, these studies suggest that functional foods and dietary bioactive compounds may protect against DOX-induced cardiotoxicity through a coordinated gut–heart mechanism. The most consistent protective processes include preservation of intestinal barrier integrity, reduction in LPS-mediated endotoxemia, restoration of beneficial microbial taxa, increased production of protective metabolites such as butyrate and indole-3-propionic acid, activation of Nrf2-dependent antioxidant defense, improvement in mitochondrial fission–fusion balance, attenuation of cardiomyocyte apoptosis, suppression of NLRP3-mediated inflammation, and inhibition of ferroptosis. These mechanisms are highly complementary to the established cardiomyocyte-centered pathways of DOX toxicity, including mitochondrial ROS generation, impaired oxidative phosphorylation, calcium dysregulation, iron-dependent lipid peroxidation, and regulated cell death. However, the translational maturity of this field remains limited. The studies discussed above are predominantly preclinical, using rodent models, H9c2 cells, fecal microbiota transfer, multi-omics profiling, and mechanistic knockout approaches. To date, there is no robust clinical trial evidence demonstrating that interventions aimed at modulation of intestinal microbiota prevent anthracycline-related cardiac dysfunction in cancer patients. Future studies should adopt a precision cardio-oncology design. An integrated approach would allow functional foods to be evaluated not simply as nonspecific antioxidants, but as microbiota-active, metabolically targeted, and mechanistically stratified adjuncts in anthracycline cardio-oncology. Taken together, the nutritional and nutraceutical strategies discussed above differ substantially in their degree of translational maturity and in the availability of direct evidence from pediatric oncology. The current evidence for the principal cardioprotective interventions, with particular emphasis on their relevance to pediatric cancer survivors, is summarized in Table 1.

## 11. Future Perspectives

Nutritional and nutraceutical strategies for prevention of OS-induced cardiotoxicity have strong biological plausibility; however, their translation into routine cardio-oncology practice remains incomplete. Most interventions discussed in this review act on overlapping mechanisms, including mitochondrial ROS production, lipid peroxidation, inflammation, endothelial dysfunction, regulated cell death pathways, fibrosis, and impaired cardiac remodeling. However, the available evidence is heterogeneous, and many promising findings are derived from cell culture or animal models rather than adequately powered clinical trials. Future research should, therefore, move beyond isolated antioxidant supplementation and toward integrated, mechanism-based, risk-adapted, and clinically validated prevention strategies. A major future direction is the development of rational combination strategies. AIC is not mediated by a single pathway but by a complex interaction between mitochondrial dysfunction, iron dysregulation, TOP2β-mediated DNA damage, oxidative/nitrosative stress, inflammation, ferroptosis, apoptosis, endothelial injury, and fibrotic remodeling. Therefore, single-agent nutritional interventions may be insufficient, particularly in high-risk patients. Combination approaches could include nutritional antioxidants together with established pharmacological cardioprotective agents, such as dexrazoxane, renin–angiotensin system inhibitors, beta-blockers, mineralocorticoid receptor antagonists, or statins, depending on baseline cardiovascular risk and treatment protocol [36,93]. Nutritional combinations should be designed according to complementary mechanisms rather than empirical supplementation. For example, omega-3 fatty acids may reduce inflammation, lipid peroxidation, and mitochondrial ROS generation, while selenium may support GPx/TXNRD-dependent antioxidant defense; CoQ10 may improve mitochondrial electron transport and membrane redox stability; and vitamin D may attenuate inflammation, endothelial-to-mesenchymal transition, calcium dysregulation, and fibrosis. Such combinations may theoretically provide broader protection than any single intervention. However, they also increase the risk of pharmacokinetic interactions, excessive antioxidant exposure, and unpredictable effects on tumor response. Thus, future studies must evaluate not only cardiac endpoints but also chemotherapy efficacy, recurrence, survival, and treatment tolerance. Early clinical data support the feasibility of nutritional cardioprotection, but the field remains underdeveloped. Omega-3 supplementation reduced early DOX-induced cardiotoxicity and improved OS markers in children with ALL [258]. Nanocurcumin supplementation showed protective effects on echocardiographic indices in breast cancer patients receiving DOX [244]. These studies provide important proof-of-concept, but they should be followed by larger trials using standardized formulations, biomarker-guided inclusion criteria, and clinically meaningful cardiac and oncological endpoints. Pediatric cardio-oncology deserves particular attention because children exposed to anthracyclines have a long lifetime during which subclinical myocardial injury may progress to overt cardiovascular disease. The developing myocardium may be especially vulnerable to OS, mitochondrial injury, and disruption of cardiomyocyte growth and maturation. In addition, cardiotoxic injury acquired during childhood may interact with somatic growth, puberty, lifestyle factors, obesity, insulin resistance, hypertension, and pregnancy later in life. Therefore, nutritional prevention strategies may have especially high long-term value in pediatric oncology, but they also require rigorous safety evaluation. Children with ALL represent a particularly relevant population because anthracyclines remain a key component of therapy and survival rates are high. The randomized trial by El Amrousy et al. is important because omega-3 fatty acids improved redox status, reduced cardiac injury biomarkers, and preserved strain-based echocardiographic parameters in children receiving DOX [258]. However, pediatric nutritional cardioprotection cannot be extrapolated directly from adult studies. Children differ in body composition, growth requirements, micronutrient needs, pharmacokinetics, chemotherapy protocols, and long-term risk profiles. Future pediatric trials should therefore include age-specific dosing, careful monitoring of growth and development, nutritional status, inflammatory and OS biomarkers, cardiac troponins, natriuretic peptides, GLS, and long-term follow-up. Importantly, nutritional interventions should be evaluated alongside established pediatric cardioprotective strategies, including risk-adapted dexrazoxane use in children expected to receive high cumulative anthracycline exposure [429]. Long-term survivorship programs are essential for translating early cardioprotection into durable clinical benefit. AIC may remain clinically silent for years or decades before manifesting as left ventricular dysfunction, heart failure, arrhythmias, ischemic heart disease, or reduced exercise capacity. International recommendations emphasize cardiomyopathy surveillance in survivors of childhood, adolescent, and young adult cancer exposed to anthracyclines or chest-directed radiotherapy [83,430]. These programs provide an ideal framework for integrating nutritional assessment, cardiometabolic risk reduction, and personalized prevention. Future survivorship care should move beyond periodic imaging alone. It should include systematic assessment of dietary quality, body composition, physical activity, blood pressure, glucose metabolism, lipid profile, vitamin D status, selenium status where relevant, omega-3 index, and markers of inflammation and OS. Such assessment is particularly important because conventional cardiovascular risk factors can amplify treatment-related cardiac vulnerability. Nutritional interventions may be most useful when they are embedded into broader survivorship programs that include exercise; weight management; smoking avoidance; sleep optimization; psychosocial support; and management of hypertension, dyslipidemia, diabetes, and obesity. Risk prediction should also become more precise. Future programs should combine imaging, biomarkers, treatment exposure, genetic susceptibility, epigenetic markers, metabolomics, microbiome profiles, and nutritional status to identify survivors most likely to benefit from preventive interventions. This approach would allow nutritional cardioprotection to be used in a targeted rather than indiscriminate manner.

The most important unmet need is the absence of large, well-designed randomized clinical trials evaluating nutritional cardioprotection during cardiotoxic cancer therapy. Many currently available studies are small; single-center; short-term; and heterogeneous in intervention type, dose, formulation, duration, population, and cardiac endpoints. In addition, many trials rely on conventional LVEF, which may remain normal until substantial myocardial injury has occurred. Future trials should therefore incorporate sensitive early endpoints such as high-sensitivity cardiac troponins, NT-proBNP, GLS, cardiac magnetic resonance imaging (MRI), OS biomarkers, inflammatory markers, and mitochondrial or lipidomic signatures. Trial design should also address several key translational questions. The interventions should be tested in clearly defined high-risk populations, including patients receiving high cumulative anthracycline doses, children, older adults, patients with pre-existing cardiovascular risk factors, and survivors with abnormal baseline biomarkers or strain. Also, trials should distinguish between correction of deficiency states and pharmacological supplementation. For example, correcting vitamin D deficiency or selenium insufficiency is conceptually different from administering high-dose active vitamin D metabolites or selenium nanoparticles. In addition, trials should standardize formulations, since bioavailability differs markedly between native compounds and nanoformulations. Moreover, future studies must simultaneously assess cardiac and oncological outcomes. This is essential because interventions that attenuate OS could theoretically reduce chemotherapy-induced tumor cell injury, although some nutraceuticals may instead enhance antitumor efficacy in selected contexts. Therefore, trials should include tumor response, recurrence, event-free survival, overall survival, chemotherapy dose intensity, treatment interruptions, and adverse events. Finally, long-term follow-up is required because prevention of early biomarker changes or strain abnormalities is clinically meaningful only if it translates into lower rates of persistent cardiac dysfunction, heart failure, cardiovascular events, and impaired quality of life.

Overall, the future of nutritional cardioprotection in cardio-oncology will likely depend on precision-based integration rather than universal supplementation. The most promising approach is to combine baseline nutritional and cardiometabolic assessment with individualized risk stratification, evidence-based dietary counseling, correction of deficiencies, targeted use of selected nutraceuticals, and close cardiac surveillance. Such a strategy may be particularly valuable in pediatric cancer survivors and other high-risk populations. However, until robust randomized clinical evidence becomes available, nutritional interventions should be considered complementary supportive strategies rather than replacements for established cardio-oncology surveillance and pharmacological cardioprotection.

## 12. Conclusions

OS represents a central, although not isolated, mechanism linking cancer therapy to progressive cardiac remodeling and development of cardiomyopathy. Current evidence indicates that selected dietary compounds and nutraceuticals may modulate several of these interconnected pathways by enhancing endogenous antioxidant defenses, preserving mitochondrial quality control and bioenergetics, limiting inflammatory and cell-death signaling, and influencing epigenetic regulation and gut microbiota composition.

However, the evidence supporting nutritional cardioprotection remains predominantly derived from in vitro and animal studies. Available clinical investigations are limited by small sample sizes, heterogeneous populations, variable formulations and doses, short follow-up periods, and inconsistent cardiovascular endpoints. Consequently, the existing evidence does not support indiscriminate antioxidant or nutraceutical supplementation during anticancer treatment. Clinical translation will require standardized interventions; assessment of baseline nutritional status; improved bioavailability; appropriate timing relative to cancer therapy; and simultaneous evaluation of cardiovascular safety, cardioprotective efficacy, and potential effects on antitumor activity. Therefore, nutritional interventions should currently be regarded as individualized complementary strategies integrated with established cardio-oncology surveillance and pharmacological cardioprotection, rather than as substitutes for evidence-based clinical care.

## Figures and Tables

**Table 1:** Translational evidence for nutritional cardioprotection with specific relevance to pediatric cancer survivors.

Intervention	Principal Cardioprotective Mechanisms	Highest Available Evidence	Evidence in Pediatric Oncology	Translational Interpretation	Ref.
Polyphenols	Activation of Nrf2/ARE and SIRT1/AMPK/PGC-1α signaling; enhancement of endogenous antioxidant defenses; preservation of mitochondrial function and bioenergetics; inhibition of NF-κB-mediated inflammation; modulation of apoptosis, ferroptosis, pyroptosis, autophagy.	Extensive in vitro and in vivo preclinical evidence; limited and heterogeneous human clinical evidence.	No robust direct pediatric clinical evidence demonstrating prevention of AIC.	Among the most extensively investigated nutritional cardioprotective classes mechanistically, but clinical translation remains limited by low/variable bioavailability, heterogeneous formulations and doses, and insufficient randomized cardio-oncology trials; pediatric efficacy remains unestablished.	[198,199,200,201,202,203,204,205,206,207,212,213,214,215,216,226,227,228,229,230]
Omega-3 PUFAs (EPA/DHA)	Reduction in mitochondrial ROS and lipid peroxidation; modulation of inflammatory signaling and pro-resolving lipid mediators; preservation of myocardial function.	Randomized clinical evidence.	Direct RCT evidence in children with ALL receiving DOX; additional randomized pediatric data on cardiometabolic outcomes and body composition.	Currently among the nutritionally based strategies with the strongest direct pediatric evidence; larger multicenter trials with long-term cardiac outcomes are required.	[258,259,260]
Coenzyme Q10	Support of mitochondrial electron transport; preservation of ATP production; membrane antioxidant activity; reduction in lipid peroxidation and mitochondrial ROS.	Small controlled pediatric study; adult phase I randomized pharmacokinetic/safety study.	Direct pediatric evidence in children with ALL and non-Hodgkin lymphoma, but based on a small historical controlled study.	Biologically plausible with a pediatric clinical signal, but efficacy remains insufficiently established using contemporary cardio-oncology endpoints.	[262,263,264,265]
Selenium	GPx/GPX4- and TXNRD-dependent antioxidant defense; Nrf2 activation; NLRP3 suppression; modulation of lipid peroxidation and ferroptotic susceptibility.	Preclinical studies plus small pediatric clinical study.	Direct pediatric clinical evidence: low selenium status was associated with cardiotoxicity; supplementation in a small subgroup was followed by improvement in NT-proBNP and/or echocardiographic findings.	Promising particularly in selenium-deficient patients, but evidence does not support indiscriminate supplementation; dose, baseline status, formulation, and oncological safety require consideration.	[271,272,273,274,275,276,277,278]
Vitamin C	Aqueous ROS scavenging; regeneration of vitamin E; reduction in oxidative/nitrosative stress; modulation of p38/JNK/p53/NF-κB signaling; preservation of endogenous antioxidant systems.	Predominantly in vitro and animal evidence; limited heterogeneous human data.	No robust direct pediatric cardioprotection trial.	Mechanistically plausible but not clinically established for prevention of AIC.	[281,282,283,284,285]
Vitamin E	Inhibition of membrane lipid peroxidation; protection of PUFA-rich cellular and mitochondrial membranes; interaction with vitamin C-dependent antioxidant recycling.	Preclinical evidence; limited adult clinical evidence, including a randomized study using vitamin E plus levocarnitine.	No robust direct pediatric evidence.	Human evidence remains difficult to interpret because vitamin E has often been combined with other interventions; the independent cardioprotective effect is uncertain.	[294,295,296,297,298,299,300]
Folate, one-carbon nutrients, and SAM	Regulation of one-carbon metabolism and methyl-donor availability; DNA and histone methylation; glutathione metabolism; eNOS coupling; mitochondrial and redox regulation.	Preclinical animal and mechanistic evidence.	No direct pediatric cardioprotection trial.	Emerging metabolic–epigenetic strategy. Biological plausibility is supported by experimental DOX models, but there is no evidence for routine clinical supplementation specifically for cardioprotection.	[396,407,408,411]
NAD^+^ precursors	Restoration of cellular NAD^+^ pools; SIRT1-dependent signaling; preservation of mitochondrial bioenergetics; improvement in lysosomal function and autophagic flux; attenuation of oxidative stress, inflammation, apoptosis, and fibrosis; modulation of de novo NAD^+^ synthesis through the kynurenine pathway.	In vivo and in vitro preclinical evidence.	No direct pediatric evidence.	Strong mechanistic rationale, particularly for mitochondrial quality control and nutrient-sensitive epigenetic regulation, but currently experimental in cardio-oncology.	[412,413,414,415,416]
Gut microbiota modulation and microbiota-active nutritional interventions	Restoration of gut-barrier integrity; reduced LPS-mediated endotoxemia; modulation of SCFAs and indole metabolites; Nrf2 activation; NLRP3 suppression; improved mitochondrial dynamics; attenuation of ferroptosis.	Predominantly animal and mechanistic evidence.	No clinical pediatric cardioprotection trials.	Promising gut–heart–axis approach, but clinical translation remains at an early stage.	[418,419,421,422,425,428]

Abbreviations: AIC—anthracycline-induced cardiotoxicity; ALL—acute lymphoblastic leukemia; AMPK—AMP-activated protein kinase; ARE—antioxidant response element; ATP—adenosine triphosphate; DHA—docosahexaenoic acid; DOX—doxorubicin; eNOS—endothelial nitric oxide synthase; EPA—eicosapentaenoic acid; GPx—glutathione peroxidase; GPX4—glutathione peroxidase 4; JNK—c-Jun N-terminal kinase; LPS—lipopolysaccharide; NAD^+^—nicotinamide adenine dinucleotide; NF-κB—nuclear factor kappa B; NLRP3—NLR family pyrin domain containing 3; Nrf2—nuclear factor erythroid 2-related factor 2; NT-proBNP—N-terminal pro–B-type natriuretic peptide; p38—p38 mitogen-activated protein kinase; p53—tumor protein p53; PGC-1α—peroxisome proliferator-activated receptor gamma coactivator 1-alpha; PUFA(s)—polyunsaturated fatty acid(s); RCT—randomized controlled trial; ROS—reactive oxygen species; SAM—S-adenosylmethionine; SCFA(s)—short-chain fatty acid(s); SIRT1—sirtuin 1; TXNRD—thioredoxin reductase.

## Data Availability

Data are available from the corresponding author upon request.

## Abbreviations

The following abbreviations are used in this manuscript:

**1,25(OH)_2_D_3_**	1,25-dihydroxyvitamin D_3_ (calcitriol)
**2-HG**	2-hydroxyglutarate
**25(OH)D**	25-hydroxyvitamin D
**5hmC**	5-hydroxymethylcytosine
**5mC**	5-methylcytosine
**ABC**	ATP-binding cassette
**ACE**	angiotensin-converting enzyme
**AKT**	protein kinase B
**ALL**	acute lymphoblastic leukemia
**ALOX5**	arachidonate 5-lipoxygenase
**α-KG**	alpha-ketoglutarate
**ALT**	alanine aminotransferase
**AMPK**	AMP-activated protein kinase
**Apaf-1**	apoptotic protease-activating factor 1
**ARE**	antioxidant response element
**ASC**	apoptosis-associated speck-like protein containing a CARD
**ACMSD**	α-amino-β-carboxy-muconate-semialdehyde decarboxylase
**AST**	aspartate aminotransferase
**ATG5**	autophagy-related protein 5
**ATM**	ataxia–telangiectasia mutated
**ATP**	adenosine triphosphate
**BAK**	BCL-2 antagonist/killer 1
**BAX**	BCL-2-associated X protein
**BCL-2**	B-cell lymphoma 2
**Bcl-xL**	B-cell lymphoma-extra large
**BCRP**	breast cancer resistance protein
**Bmi-1**	BMI1 proto-oncogene, polycomb ring finger
**BNP**	B-type natriuretic peptide
**BTG2**	B-cell translocation gene 2
**CARD**	caspase activation and recruitment domain
**CCSS**	Childhood Cancer Survivor Study
**CDK1**	cyclin-dependent kinase 1
**CGI(s)**	CpG island(s)
**CHKB-DT**	choline kinase beta divergent transcript
**circRNA(s)**	circular RNA(s)
**CK**	creatine kinase
**CK-MB**	creatine kinase-MB isoenzyme
**CoA**	coenzyme A
**CoQ10**	coenzyme Q10
**CpG**	cytosine–phosphate–guanine dinucleotide
**CpH**	non-CpG cytosine context in which H denotes A, C, or T
**cTnT**	cardiac troponin T
**Cyt c**	cytochrome c
**DHA**	docosahexaenoic acid
**DLBCL**	diffuse large B-cell lymphoma
**DMS**	differentially methylated sites
**DNA**	deoxyribonucleic acid
**DNMT(s)**	DNA methyltransferase(s)
**DNMT1**	DNA methyltransferase 1
**DNMT3A**	DNA methyltransferase 3 alpha
**DNMT3B**	DNA methyltransferase 3 beta
**DOX**	doxorubicin
**Drp1**	dynamin-related protein 1
**EDTA**	ethylenediaminetetraacetic acid
**EGCG**	epigallocatechin-3-gallate
**EGFR**	epidermal growth factor receptor
**EndMT**	endothelial-to-mesenchymal transition
**EPA**	eicosapentaenoic acid
**ERK1/2**	extracellular signal-regulated kinases 1 and 2
**ETC**	electron transport chain
**ETS**	electron transport system
**Fas**	Fas cell surface death receptor
**FasL**	Fas ligand
**FDA**	US Food and Drug Administration
**Fis1**	mitochondrial fission protein 1
**FMT**	fecal microbiota transplantation
**Fn14**	fibroblast growth factor-inducible 14
**γH2AX**	phosphorylated histone H2AX
**GCLC**	glutamate–cysteine ligase catalytic subunit
**GCLM**	glutamate–cysteine ligase modifier subunit
**GLS**	global longitudinal strain
**GPx**	glutathione peroxidase
**GPX4**	glutathione peroxidase 4
**GSDMD**	gasdermin D
**GSH**	glutathione
**H2AK119Ub**	monoubiquitination of histone H2A at lysine 119
**H2AX**	histone H2A variant X
**H3K27ac**	acetylation of histone H3 lysine 27
**H3K27me3**	trimethylation of histone H3 lysine 27
**H3K4**	histone H3 lysine 4
**H3K4me1**	monomethylation of histone H3 lysine 4
**H3K4me3**	trimethylation of histone H3 lysine 4
**H9c2**	rat embryonic cardiomyoblast cell line
**HAT(s)**	histone acetyltransferase(s)
**HAX-1**	HCLS1-associated protein X-1
**HCM-ls**	human cardiomyocyte-like cells
**HDAC(s)**	histone deacetylase(s)
**HDM(s)**	histone demethylase(s)
**HER2**	human epidermal growth factor receptor 2
**HF**	heart failure
**HINT2**	histidine triad nucleotide-binding protein 2
**HM(s)**	histone modification(s)
**HMT(s)**	histone methyltransferase(s)
**HO-1**	heme oxygenase-1
**HUVEC(s)**	human umbilical vein endothelial cell(s)
**ICAM-1**	intercellular adhesion molecule-1
**IDH**	isocitrate dehydrogenase(s)
**IDO1**	indoleamine 2,3-dioxygenase 1
**IGF-1**	insulin-like growth factor 1
**IL**	interleukin
**iNOS**	inducible nitric oxide synthase
**JMJD3**	Jumonji domain-containing protein 3
**JNK**	c-Jun N-terminal kinase
**Keap1**	Kelch-like ECH-associated protein 1
**LC3I/II**	microtubule-associated protein 1 light chain 3 I/II
**LDH**	lactate dehydrogenase
**LGE**	late gadolinium enhancement
**lncRNA(s)**	long non-coding RNA(s)
**LPS**	lipopolysaccharide
**LVEF**	left ventricular ejection fraction
**MAPK(s)**	mitogen-activated protein kinase(s)
**MCM-41**	Mobil Composition of Matter No. 41
**MCP-1**	monocyte chemoattractant protein-1
**mCpH**	methylated CpH
**MDA**	malondialdehyde
**MeCP2**	methyl-CpG-binding protein 2
**Mfn2**	mitofusin 2
**miR/miRNA(s)**	microRNA(s)
**MitoQ**	mitoquinone
**MnSOD**	manganese superoxide dismutase
**mPTP**	mitochondrial permeability transition pore
**MRI**	magnetic resonance imaging
**mRNA(s)**	messenger RNA(s)
**MRP1**	multidrug resistance-associated protein 1
**mtDNA**	mitochondrial DNA
**mTOR**	mechanistic target of rapamycin
**mTORC1**	mechanistic target of rapamycin complex 1
**NAC**	N-acetylcysteine
**NAD^+^**	nicotinamide adenine dinucleotide
**NADPH**	reduced nicotinamide adenine dinucleotide phosphate
**ncRNA(s)**	non-coding RNA(s)
**NF-κB**	nuclear factor kappa B
**NLRP3**	NLR family pyrin domain-containing 3
**NO**	nitric oxide
**NORAD**	non-coding RNA activated by DNA damage
**NOX4**	NADPH oxidase 4
**NQO1**	NAD(P)H quinone oxidoreductase 1
**NRF1/2**	nuclear respiratory factors 1 and 2
**Nrf2**	nuclear factor erythroid 2-related factor 2
**NT-proBNP**	N-terminal pro-B-type natriuretic peptide
**OPA1**	optic atrophy 1
**OS**	oxidative stress
**p21**	cyclin-dependent kinase inhibitor 1A
**p53**	tumor protein p53
**PAGln**	phenylacetylglutamine
**PGC-1α**	peroxisome proliferator-activated receptor gamma coactivator 1-alpha
**PI3K**	phosphoinositide 3-kinase
**PINK1**	PTEN-induced kinase 1
**piRNA(s)**	PIWI-interacting RNA(s)
**PTM(s)**	post-translational modification(s)
**PTPN2**	protein tyrosine phosphatase non-receptor type 2
**PUFA(s)**	polyunsaturated fatty acid(s)
**QPRT**	quinolinate phosphoribosyltransferase
**Rac1**	Ras-related C3 botulinum toxin substrate 1
**Res-SLN**	resveratrol-loaded solid lipid nanoparticles
**RNA**	ribonucleic acid
**RNS**	reactive nitrogen species
**ROS**	reactive oxygen species
**rRNA(s)**	ribosomal RNA(s)
**SAM**	S-adenosylmethionine
**SCFA(s)**	short-chain fatty acid(s)
**Se@CM**	selenium-enriched *Cordyceps militaris*-based oral microcarrier
**SERCA**	sarco/endoplasmic reticulum Ca^2+^-ATPase
**SESN2**	sestrin 2
**SGK1**	serum/glucocorticoid-regulated kinase 1
**siRNA(s)**	small interfering RNA(s)
**SIRT(s)**	sirtuin(s)
**SJLIFE**	St. Jude Lifetime Cohort Study
**SKQ1**	mitochondria-targeted plastoquinone derivative
**sncRNA(s)**	small non-coding RNA(s)
**snoRNA(s)**	small nucleolar RNA(s)
**snRNA(s)**	small nuclear RNA(s)
**SOD**	superoxide dismutase
**STAT**	signal transducer and activator of transcription
**TET(s)**	ten-eleven translocation enzyme(s)
**TET1**	ten-eleven translocation methylcytosine dioxygenase 1
**TET2**	ten-eleven translocation methylcytosine dioxygenase 2
**TET3**	ten-eleven translocation methylcytosine dioxygenase 3
**TF(s)**	transcription factor(s)
**TFAM**	mitochondrial transcription factor A
**TFEB**	transcription factor EB
**TGF-β**	transforming growth factor beta
**TINCR**	terminal differentiation-induced non-coding RNA
**TNF-α**	tumor necrosis factor alpha
**TOP2α**	topoisomerase II alpha
**TOP2β**	topoisomerase II beta
**TPGS**	D-α-tocopheryl polyethylene glycol 1000 succinate
**tRNA(s)**	transfer RNA(s)
**TUNEL**	terminal deoxynucleotidyl transferase dUTP nick-end labeling
**TWEAK**	TNF-like weak inducer of apoptosis
**TXNRD**	thioredoxin reductase
**VC/VK3**	ascorbate and menadione sodium bisulfite
**VCAM-1**	vascular cell adhesion molecule-1
**VDR**	vitamin D receptor
**Xc^−^**	cystine/glutamate antiporter system Xc^−^
**YAP**	Yes-associated protein
**ZO-1**	zonula occludens-1
